# Supplementary material for: Work-related psychosocial risk factors for stress-related mental disorders: an updated systematic review and meta-analysis
Source: BMJ Open. 2020 Jul 5;10(7):e034849. doi: 10.1136/bmjopen-2019-034849 (PMC7337889; doi:10.1136/bmjopen-2019-034849)
Supplement: Supplementary data [file bmjopen-2019-034849supp001.pdf]

Appendix Search strategy Psychosocial risk factors and SRDs 12-08-2019
===
Medline
===
1. exp Occupations/ or Workload/ or exp Work/ or Workplace/ or exp Occupational Diseases/ or Rehabilitation, Vocational/ or Occupational Health/ or Sick Leave/ or Absenteeism/ or Retirement/ or workers' compensation/ or exp Employment/ or exp Occupational Exposure/ or Volunteers/
2. (worka* or worke* or workg* or worki* or workl* or workp* or work capacity or work disabilit* or work abilit* or at work or work exposure or work related or workers or job* or employee or staff or personnel or occupation or occupations or occupational or outdoor work* or day shift* or night shift* or shift work* or vocational rehabilitation or sick leave or absenteeism or sickness absen* or absente* or presente* or return to work or vocational reintegration or retirement or pension or employment or unemployed or unemployment or work status or industries or volunteer* or voluntary worker*).ab,kw,ti.
3. or/1-2 [working population]
4. risk/ or risk assessment/ or risk factors/ or cohort studies/ or Follow-Up Studies/
5. (risk* or Case control or cohort stud* or Cohort analy* or follow up stud* or observational stud* or Longitudinal).ab,kw,ti.
6. or/4-5 [aetiology]
7. Burnout, Professional/ or neurasthenia/ or adjustment disorders/
8. (burnout or burn out or neurasthenia or adjustment disorder? or emotional exhaustion or job stress or work stress or occupational stress or job strain or work strain).ab,kw,ti.
9. (occupational health/ or occupational disease/) and (psychiatric disorder? or psychiatric morbidity or psychosocial or psycho social or occupational stress or distress).ab,kw,ti.
10. or/7-9 [burnout]
11. (stress symptom* or psychologic* stress or psychologic* distress or psychosocial risk* or psycho social risk* or psychosocial factor* or psycho social factor* or psychiatric morbidit* or psychiatric disorder*).ab,kw,ti. [stress related symptoms and disorders]
12. 3 and 6 and 10
13. ((worka* or worke* or workg* or worki* or workl* or workp* or work capacity or work disabilit* or work abilit* or at work or work exposure or work related or workers or job* or employee or staff or personnel or occupation or occupations or occupational or outdoor work* or day shift* or night shift* or shift work* or vocational rehabilitation or sick leave or absenteeism or sickness absen* or absente* or presente* or return to work or vocational reintegration or retirement or pension or employment or unemployed or unemployment or work status or industries or volunteer* or voluntary worker*) adj1 (stress symptom* or psychologic* stress or psychologic* distress or psychosocial risk* or psycho social risk* or psychosocial factor* or psycho social factor* or psychiatric morbidit* or psychiatric disorder*)).ab,kw,ti.
14. 6 and 13
15. ((risk* or Case control or cohort stud* or Cohort analy* or follow up stud* or observational stud* or Longitudinal) adj1 (stress symptom* or psychologic* stress or psychologic* distress or psychosocial risk* or psycho social risk* or psychosocial factor* or psycho social factor* or psychiatric morbidit* or psychiatric disorder*)).ab,kw,ti.
16. 3 and 15
17. 12 or 14 or 16
18. limit 17 to (dutch or english or german)

===
PsycINFO
===
1. (occupational employment testing or occupational interests guidance or occupational vocational rehabilitation or working conditions industrial safety).cc.
2. exp occupations/ or career change/ or career development/ or exp "division of labor"/ or employment history/ or exp job characteristics/ or occupational adjustment/ or occupational aspirations/ or occupational attitudes/ or occupational choice/ or occupational exposure/ or occupational guidance/ or occupational interests/ or occupational mobility/ or occupational preference/ or occupational safety/ or occupational status/ or occupational success/ or exp occupational tenure/ or exp personnel/ or exp professional personnel/ or working women/ or "work (attitudes toward)"/ or Workplace Violence/ or Diversity in the Workplace/ or exp working conditions/ or exp vocational rehabilitation/ or Occupational Health/ or employee absenteeism/ or Retirement/ or exp employment status/ or reemployment/ or Volunteers/
3. (worka* or worke* or workg* or worki* or workl* or workp* or work capacity or work disabilit* or work abilit* or at work or work exposure or work related or workers or job* or employee or staff or personnel or occupation or occupations or occupational or outdoor work* or day shift* or night shift* or shift work* or vocational rehabilitation or sick leave or absenteeism or sickness absen* or absente* or presente* or return to work or vocational reintegration or retirement or pension or employment or unemployed or unemployment or work status or industries or volunteer* or voluntary worker*).ab,id,ti.
4. or/1-3 [working population]

van der Molen HF, *et al. BMJ Open* 2020; 10:e034849. doi: 10.1136/bmjopen-2019-034849

5. Risk Assessment/ or At Risk Populations/ or Risk Factors/ or clinical trials/ or cohort analysis/ or followup studies/ or exp longitudinal studies/
6. (risk* or Case control or cohort stud* or Cohort analy* or follow up stud* or observational stud* or Longitudinal).ab,id,ti.
7. or/5-6 [aetiology]
8. occupational stress/ or work related illnesses/ or neurastenia/ or adjustment disorders/
9. (neurasthenia or burnout or burn out or emotional exhaustion or job stress or work stress or occupational stress or job strain or work strain or adjustment disorder?).ab,id,ti.
10. 8 or 9 [burnout]
11. (stress or distress).ab,id,ti.
12. 4 and 7 and 10
13. ((risk* or Case control or cohort stud* or Cohort analy* or follow up stud* or observational stud* or Longitudinal) adj3 (stress or distress)).ab,id,ti.
14. 4 and 13
15. 12 or 14
16. limit 15 to ("0100 journal" or "0110 peer-reviewed journal" or "0120 non-peer-reviewed journal" or "0130 peer-reviewed status unknown" or "0400 dissertation abstract")
17. limit 16 to (dutch or english or german)

==
Embase
==
1. exp *occupation/ or exp *occupational health/ or *work/ or *workplace/ or *"employment of women"/ or exp *income/ or *workman compensation/ or exp *employment/ or exp *"named groups by occupation"/
2. (worka* or worke* or workg* or worki* or workl* or workp* or work capacity or work disabilit* or work abilit* or at work or work exposure or work related or workers or job* or employee or staff or personnel or occupation or occupations or occupational or outdoor work* or day shift* or night shift* or shift work* or vocational rehabilitation or sick leave or absenteeism or sickness absen* or absente* or presente* or return to work or vocational reintegration or retirement or pension or employment or unemployed or unemployment or work status or industries or volunteer* or voluntary worker*).ab,kw,ti.
3. or/1-2 [working population]
4. *risk/ or *risk assessment/ or *risk factor/ or *cohort analysis/ or *Follow-Up/
5. (risk* or Case control or cohort stud* or Cohort analy* or follow up stud* or observational stud* or Longitudinal).ab,kw,ti.
6. or/4-5 [etiology]
7. *Burnout/ or *neurasthenia/ or *adjustment disorder/ or *emotional stress/ or *job stress/
8. (burnout or burn out or neurasthenia or adjustment disorder? or emotional exhaustion or job stress or work stress or occupational stress or job strain or work strain).ab,kw,ti.
9. (occupational health/ or occupational disease/) and (psychiatric disorder? or psychiatric morbidity or psychosocial or psycho social or occupational stress or distress).ab,kw,ti.
10. or/7-9 [burnout]
11. (stress symptom* or psychologic* stress or psychologic* distress or psychosocial risk* or psycho social risk* or psychosocial factor* or psycho social factor* or psychiatric morbidit* or psychiatric disorder*).ab,kw,ti. [stress related symptoms and disorders]
12. 3 and 6 and 10
13. ((worka* or worke* or workg* or worki* or workl* or workp* or work capacity or work disabilit* or work abilit* or at work or work exposure or work related or workers or job* or employee or staff or personnel or occupation or occupations or occupational or outdoor work* or day shift* or night shift* or shift work* or vocational rehabilitation or sick leave or absenteeism or sickness absen* or absente* or presente* or return to work or vocational reintegration or retirement or pension or employment or unemployed or unemployment or work status or industries or volunteer* or voluntary worker*) adj1 (stress symptom* or psychologic* stress or psychologic* distress or psychosocial risk* or psycho social risk* or psychosocial factor* or psycho social factor* or psychiatric morbidit* or psychiatric disorder*)).ab,kw,ti.
14. 6 and 11 and 13
15. ((risk* or Case control or cohort stud* or Cohort analy* or follow up stud* or observational stud* or Longitudinal) adj1 (stress symptom* or psychologic* stress or psychologic* distress or psychosocial risk* or psycho social risk* or psychosocial factor* or psycho social factor* or psychiatric morbidit* or psychiatric disorder*)).ab,kw,ti.
16. 3 and 15
17. 12 or 14 or 16
18. limit 17 to (dutch or english or german)

van der Molen HF, *et al. BMJ Open* 2020; 10:e034849. doi: 10.1136/bmjopen-2019-034849
