# Supplementary material for: Work-related psychosocial risk factors for stress-related mental disorders: an updated systematic review and meta-analysis
Source: BMJ Open. 2020 Jul 5;10(7):e034849. doi: 10.1136/bmjopen-2019-034849 (PMC7337889; doi:10.1136/bmjopen-2019-034849)
Supplement: Author's manuscript [file bmjopen-2019-034849.draft_revisions.pdf]

BMJ Open is committed to open peer review. As part of this commitment we make the peer review history of every article we publish publicly available.

When an article is published we post the peer reviewers' comments and the authors' responses online. We also post the versions of the paper that were used during peer review. These are the versions that the peer review comments apply to.

The versions of the paper that follow are the versions that were submitted during the peer review process. They are not the versions of record or the final published versions. They should not be cited or distributed as the published version of this manuscript.

BMJ Open is an open access journal and the full, final, typeset and author-corrected version of record of the manuscript is available on our site with no access controls, subscription charges or pay-per-view fees (http://bmjopen.bmj.com).

If you have any questions on BMJ Open's open peer review process please email info.bmjopen@bmj.com

Work-related psychosocial risk factors for stress-related disorders – a systematic review and meta-analysis

Journal:	*BMJ Open*
Manuscript ID	bmjopen-2019-034849
Article Type:	Original research
Date Submitted by the Author:	08-Oct-2019

Complete List of Authors:	van der Molen, Henk; Amsterdam UMC - Locatie AMC, Coronel Institute of Occupational Health<br>Nieuwenhuijsen, Karen; Amsterdam UMC - Locatie AMC, Coronel Institute of Occupational Health<br>Frings-Dresen, Monique; Amsterdam UMC - Locatie AMC, Coronel Institute of Occupational Health<br>de Groene, Gerda; Amsterdam UMC - Locatie AMC, Coronel Institute of Occupational Health

Keywords:	OCCUPATIONAL & INDUSTRIAL MEDICINE, EPIDEMIOLOGY, PREVENTIVE MEDICINE

SCHOLARONE™
Manuscripts

I, the Submitting Author has the right to grant and does grant on behalf of all authors of the Work (as defined in the below author licence), an exclusive licence and/or a non-exclusive licence for contributions from authors who are: i) UK Crown employees; ii) where BMJ has agreed a CC-BY licence shall apply, and/or iii) in accordance with the terms applicable for US Federal Government officers or employees acting as part of their official duties; on a worldwide, perpetual, irrevocable, royalty-free basis to BMJ Publishing Group Ltd ("BMJ") its licensees and where the relevant Journal is co-owned by BMJ to the co-owners of the Journal, to publish the Work in this journal and any other BMJ products and to exploit all rights, as set out in our licence.

The Submitting Author accepts and understands that any supply made under these terms is made by BMJ to the Submitting Author unless you are acting as an employee on behalf of your employer or a postgraduate student of an affiliated institution which is paying any applicable article publishing charge ("APC") for Open Access articles. Where the Submitting Author wishes to make the Work available on an Open Access basis (and intends to pay the relevant APC), the terms of reuse of such Open Access shall be governed by a Creative Commons licence – details of these licences and which Creative Commons licence will apply to this Work are set out in our licence referred to above.

Other than as permitted in any relevant BMJ Author's Self Archiving Policies, I confirm this Work has not been accepted for publication elsewhere, is not being considered for publication elsewhere and does not duplicate material already published. I confirm all authors consent to publication of this Work and authorise the granting of this licence.

For peer review only

Work-related psychosocial risk factors for stress-related disorders – a systematic review and meta-analysis

Henk F. van der Molen[1], Karen Nieuwenhuijsen[1], Monique H.W. Frings-Dresen[1], Gerda de Groene[1]

[1]Amsterdam UMC, University of Amsterdam, Department: Coronel Institute of Occupational Health, University of Amsterdam, Netherlands Center for Occupational Diseases, Amsterdam Public Health research institute, PO Box 22660, 1100 DD Amsterdam, the Netherlands

[2] Corresponding author

h.f.vandermolen@amsterdamumc.nl

For peer review only

For peer review only - http://bmjopen.bmj.com/site/about/guidelines.xhtml

Abstract

Objectives

The objective of this systematic review and meta-analysis is to examine which work-related psychosocial risk factors are associated with stress-related disorders (SRD).

Methods

We searched the electronic databases of Medline, Embase and PsycINFO for articles published between 2008 and 12 August 2019 and included the references of a systematic review performed for the period before 2008. Primary longitudinal studies were included when outcome data were described in terms of SRD assessment or a dichotomous outcome, based on a validated questionnaire, and at least two levels of work-related exposure were reported (exposed versus less or non-exposed). Two authors independently selected studies, extracted data and assessed study quality. We performed meta-analyses and used GRADE to assess the evidence for the associations between risk factors and the onset of SRD.

Results

Seventeen studies met the inclusion criteria. In total, a population of 73,874 workers from Belgium, Denmark, England, Finland, Japan, the Netherlands and Sweden were included in the meta-analysis of fourteen prospective cohort studies. This meta-analysis revealed moderate evidence for associations between SRD and effort reward imbalance (OR=1.9, 95% CI 1.70-2.15), high job demands (OR=1.6, 95% CI 1.41-1.72), organisational justice (OR's 1.6 to 1.7, CI's 1.44-1.86), social support (OR's 1.3 to 1.4, CI's 1.16-1.69), high emotional demands (OR=1.6, 95% CI 1.35-1.84), and decision authority (OR=1.3, CI 1.20-1.49). No significant or inconsistent associations were found for job insecurity, decision latitude, skill discretion and bullying.

Conclusions

Moderate evidence was found that work-related psychosocial risk factors are associated with a higher risk of SRD. Effort-reward imbalance, low organisational justice and high job demands exhibited the largest increased risk of SRD, varying from 60 to 90%.

Keywords

Mental, Stress Related Disorders, Work, Risk Factors, Meta-analysis, Aetiology

For peer review only - http://bmjopen.bmj.com/site/about/guidelines.xhtml

Strengths and limitations of this study

► Stress related disorders are frequently reported in the working population, however the work attribution is often not assessed.

► This systematic review, including a meta-analysis of prospective cohort studies, gives a valuable overview which work-related psychosocial risk factors increase the risk of stress related disorders.

► The identified psychosocial work-related risk factors are the starting point for evidence based universal and selective preventive measures to reduce work-related stress disorders.

► Limitations are the lack of harmonized assessments of stress related disorders and detailed exposure assessment in the identified observational cohort studies.

For peer review only

Introduction

Stress related disorders (SRD) are frequently reported in the working population, with varying incidence rates of 13% for psychological distress [1], 22% for emotional exhaustion [2], and a prevalence of distress symptoms of up to 50% in specific professions and countries [3]. SRD represent a significant part of work-related common mental disorders in both self-report surveys and notification systems for occupational diseases [4]. The World Health Organization has recently announced [5] that burn-out is included as 'an occupational phenomenon' in the 11th revision of the International Classification of Diseases (ICD-11).

In many countries, SRD are not notified as occupational diseases [6], mainly because of their multifactorial origin [7] or discussion about the medical status (as with burn-out) [5,8,9]. Evidence-based knowledge about work-related factors could be used for facilitating a decision on the 'work-relatedness' of an individual case of SRD in a reporting scheme or concerning the selection and implementation of preventive workplace interventions around factors with the strongest effect on SRD [7].

In the Netherlands, SRD cover diagnoses of adjustment disorders (ICD-10: F43.2) and burn-out as a state of exhaustion (ICD-10: Z730) [4]. These diagnoses differ from just stress symptoms since they are clinically assessed and have in common that they reflect a status where subjective and emotional disturbance interfere with social functioning and performance, arising from a period of adaptation to stressful conditions. Symptoms are what a worker experiences, and these symptoms might negatively affect normal or regular functioning of a person – a so called disorder. To distinguish between symptoms and disorders, we considered a SRD as diagnosis following a clinical anamnesis, stress complaints as assessed in a score above a cut-off point on a validated questionnaire or absenteeism from work due to stress problems. Work-related psychosocial risk factors can contribute to the onset of SRD [10].

For peer review only

The high and increasing burden of SRD strengthens the urgency of up-to-date insight into work-related psychosocial risk factors associated with SRD and based on longitudinal study designs in order to initiate relevant primary and secondary preventive interventions at worksites. Previous systematic reviews aiming to determine work-related risk factors for SRD have either not been updated since 2008 [10], combined various common mental disorders, such as anxiety, depression and stress-related disorders, as outcome measures [11] or used other outcomes, such as suicide [12]. The aim of our updated [10] review was to identify psychosocial risk factors at work which are associated with the onset of SRD and to examine: (1) which work-related psychosocial risk factors contribute to the onset of SRD and (2) to what extent these risk factors are associated with SRD.

Methods

This review followed the PRISMA Statement (see appendix). This review was not registered in Prospero, but an update of a previous review by Nieuwenhuijsen et al. [10].

Patient and public involvement

We analysed published cohort studies and did not seek information from individuals. In other words, there was no patients or public involvement in the study.

Study selection

Eligibility criteria

Primary prospective cohort studies were included where outcome data were described in terms of clinically or questionnaire-assessed stress-related disorder (present or not) and at least two levels of work-related exposure (exposed versus less or non-exposed) among a working population were reported in order to be able to retrieve or calculate a risk estimate.

For peer review only - http://bmjopen.bmj.com/site/about/guidelines.xhtml

This updated systematic review [10] considered studies to have included SRD if the outcome was either i) a SRD diagnosis following a clinical anamnesis, ii) a high level of stress complaints as assessed in a score above a cut-off point on a validated questionnaire for fatigue, stress or non-specific mental ill-health, or iii) absenteeism from work due to stress problems. Based on findings of Nieuwenhuijsen et al [10] and identified factors stemming from theoretical stress models, we defined seven types of exposure (i) job demands, (ii) job control (decision latitude, decision authority, skill discretion), (iii) social support (co-worker, supervisor, both), (iv) emotional demands, v) organisational justice (procedural and relational), (vi) effort-reward imbalance and (vii) other psychosocial risk factors, e.g. bullying.

Studies that described work-related risk factors in terms of psychosocial risk factors were eligible for inclusion. The studies had to describe workers in a real workplace setting. All types of exposure assessment were eligible for inclusion: self-reports or researcher observations or direct measurements. No additional criteria were formulated regarding latency between exposure and the presence or onset of the disorder or adjustment for confounders.

Data sources and search terms

We searched the electronic databases of Medline, Embase and PsychInfo for studies between 2008 and 12 August 2019 as described in supplement Appendix 1. Our PICO can be stated as: P = working population, I/C exposed / less or none exposed to a-priori defined exposure categories, O = stress related disorder. Eligible studies before 2008 were retrieved from the systematic review by Nieuwenhuijsen et al. [10].

For peer review only - http://bmjopen.bmj.com/site/about/guidelines.xhtml

Data collection and analyses

Study selection process

Titles and abstracts were independently screened by two reviewers to identify potentially relevant studies. We used a free online software tool to screen and assess references (https://rayyan.qcri.org/welcome). The full texts of potentially relevant articles were assessed for eligibility against the inclusion criteria. Disagreement between review authors on the selection of studies for inclusion occurred in less than 5% of the references screened and was resolved by discussion.

Data extraction and management

Data were extracted by one review author (GG) and checked by another review author (HM). Data on the following was extracted from each article: author; country of study; study design; case definition of stress-related disorder; sources and number of participants; exposure definition; exposure assessment; exposure categories; risk estimate; and adjustment for confounders.

Methodological quality assessment

For the assessment of the risk of bias within studies, the quality criteria from the systematic review by Nieuwenhuijsen et al. [10] were used for all study designs, with nine items covering the selection of participants, measurement of variables and control for confounding. The quality of the studies was independently assessed and agreed by two review authors (GG, HM). In total, nine items across five categories were evaluated for quality assessment: (1) the main features of the study population were stated e.g. sex, age and work context; (2) the participation rate at baseline was at least 50%; (3) the response at follow-up was at least 70% or the non-response

For peer review only - http://bmjopen.bmj.com/site/about/guidelines.xhtml

was not selective; (4) the data on psychosocial work factors was collected using standardised instruments; (5) the data on outcome were collected at least at three different time points; (6) the statistical model used was appropriate for the outcome studied; (7) the measures of association with the statistical model were presented; (8) there were study controls for confounding with rationale; (9) the number of cases was at least 10 times the number of independent variables in the analysis. The criteria for each item were scored with ‘positive’, ‘negative’ or ‘not clear’. A higher quality of a study was defined as ≥5 items scored as ‘positive’ out of the nine quality criteria.

Data synthesis

A descriptive analysis of all studies was performed, summarised, classified in categories of psychosocial risk factors, and assessed for methodological quality. Risk estimates and the corresponding 95% confidence intervals (CI) of the association between work-related factors and SRD were extracted and summarised. After assessing probable heterogeneity of risk factor and outcome assessment, all authors discussed and decided on the risk estimates to be included in the meta-analysis or should be analysed separately. We used the rule at least two studies when no statistical heterogeneity and no heterogeneity in measures of risk factors and stress related disorders was found.

Meta-analyses and quality of evidence

The selection of the work-related risk factors in the meta-analyses was based on: (1) sufficient contrast between reported exposure categories, i.e. low versus high exposure; (2) effect estimates controlled for other non-work-related factors, as reported in the primary studies; 3) homogeneity in definition or measurement of risk factors.

For peer review only

For peer review only - http://bmjopen.bmj.com/site/about/guidelines.xhtml

The meta-analysis was performed in line with the procedure described in Watenabe et al. [13]: by using a log-transformed OR and its SE in Review Manager (Cochrane Review Manager version 5.3). For the main analysis, the main ORs and the SEs from selected studies were subjected to a random-effects model meta-analysis to estimate a pooled OR and its 95% CI.

The quality of evidence - also taken into account the risk of bias across studies - was assessed using the GRADE (Grades of Recommendations, Assessment, Development and Evaluation) framework for prognostic studies developed by Huguet et al. [14]. Our starting point for the quality of the evidence was 'high' for longitudinal studies that sought to confirm independent associations between the prognostic factor and the outcome ('Phase 2' explanatory studies). The evidence could decrease on the basis of five factors: study limitations, inconsistency, indirectness, imprecision and publication bias. Moreover, two factors: (1) study findings with moderate or large effect sizes (i.e. lower limit of 95% CI OR>2.0) or (2) an exposure-response gradient, could lead to an upgrade of the quality of evidence. Four levels of quality were used: high, moderate, low and very low.

Results

Selected studies

A PRISMA flow diagram of the study selection process is shown in Figure 1. After excluding duplicates, 10,801 references were retrieved from the databases and the systematic review by Nieuwenhuijsen et al. [10], and assessed based on title and abstract. The full texts of 139 potentially eligible articles were then examined, of which 17 studies from 16 articles met the inclusion criteria.

Psychosocial risk factors and SRD

For peer review only - http://bmjopen.bmj.com/site/about/guidelines.xhtml

In total, seventeen longitudinal studies in sixteen articles[1-3, 15-27] described the association between exposure to psychosocial risk factors and the occurrence of clinically assessed SRD. The characteristics of the outcome definition (SRD), exposure definition and reported associations are presented in table 1.

For peer review only

For peer review only - http://bmjopen.bmj.com/site/about/guidelines.xhtml

Table 1. Associations between psychosocial risk factors at work and the occurrence of stress related disorders (17 studies)

Author, Country	Outcome	Exposure		
**Population**	**Definition & assessment**	**Definition & assessment**	**OR**	**(95%CI)**
Mino 1999 [15]	General Health Questionnaire (GHQ-30)	Job demands: one item	♂ RR 1.39	0.92-2.10
Japan, machine	GHQ-30 ≥ 8	*(always/sometimes present vs absent)*	♀ RR 1.14	0.81-1.59
production, N=310		Supervisor support: one item	♂ RR 1.10	0.69-1.74
		(absent vs always/sometimes present)	♀ RR 2.21	1.25-3.89
Stansfeld 1999 [16]	General Health Questionnaire (GHQ-30)	Job demands: adapted JCQ	♂ 1.33	1.1-1.6
England, civil servants,	GHQ-30 > 4	*(highest tertile vs lowest)*	♀ 1.24	1.0-1.6
N=10,308		Decision authority: adapted JCQ	♂ 1.29	1.1-1.5
		(*lowest vs highest tertile*)	♀ 1.37	1.1-1.8
		Co-worker support: adapted JCQ	♂ 1.29	1.1-1.5
		(*lowest vs highest tertile*)	♀ 1.12	0.9-1.4
		Supervisor support: adapted JCQ	♂ 1.31	1.1-1.5
		(*lowest vs highest tertile*)	♀ 1.11	0.9-1.3
		Effort-reward imbalance: indicator of high effort and low rewards	♂ 2.57	1.8-3.6
		(high efforts/low rewards vs no high efforts/low rewards)	♀ 1.67	1.0-2.9
		Skill discretion: adapted JCQ	♂ 1.11	0.9-1.3
		(*lowest vs highest tertile*)	♀ 1.09	0.8-1.4
Bültmann 2002 [1]	General Health Questionnaire	Job demands Questionnaire (JCQ)		
Netherlands, 45	GHQ-12 ≥ 4	Job demands: JCQ	♂ 1.51	1.23-1.85
companies, N=8,833		(*highest vs lowest tertile*)	♀ 1.44	1.03-2.01
		Decision latitude: JCQ	♂ 1.14	0.9-1.43
		(*lowest vs highest tertile*)	♀ 0.88	0.62-1.24
		Co-worker support: JCQ	♂ 1.25	1.04-1.49
		(low vs high)	♀ 1.31	0.97-1.78
		Supervisor support: JCQ	♂ 1.25	1.05-1.49
		(low vs high)	♀ 1.12	0.85-1.47
		Emotional demands: Dutch Questionnaires; Perception and Judgement of	♂ 1.73	1.40-2.14
		Work (QPJW), Work and Health, self-formulated *(high (2-5) vs no (0,1)*	♀ 1.39	1.01-1.91
		Job insecurity: QPJW, one-item	♂ 1.63	1.18-2.27
		(*yes vs no*)	♀ 0.94	0.56-1.59
Bonde 2005 [17]	Setterlind Stress Profile Inventory (SPI)	Skill discretion: Repetitive work	1.3	0.6-2.2

For peer review only

Denmark, various workplaces, N=2,846	SSPI ≥ 4	*(yes vs no)*		
Godin 2005 [18]<br>Belgium, privat and public sector, N=1,521	Short Fatigue Inventory (SHI)<br>SHI: upper quartile	Effort-Reward Imbalance Questionnaire<br>*(highest quartile vs rest)*	♂ 3.4<br>♀ 2.0	1.7-6.7<br>0.9-4.1
Kivimaki 2007 [19]<br>Finland, government N=21,271	General Health Questionnaire<br>GHQ-12 ≥ 4	Effort-Reward Imbalance Questionnaire<br>*(highest vs lowest quartile)*<br>Procedural & organisational injustice: Organizational Justice Scale<br>*(highest vs lowest quartile)*<br>Relational injustice: Organizational Justice Scale<br>*(highest vs lowest quartile)*	2.04<br><br>1.81<br><br>1.50	1.80-2.32<br><br>1.60-2.06<br><br>1.32-1.70
Kivimaki 2007 [19]<br>Finland, hospital N=10,736	General Health Questionnaire<br>GHQ-12 ≥ 4	Effort-Reward Imbalance Questionnaire<br>*(highest vs lowest quartile)*<br>Procedural injustice: Organizational Justice Questionnaire (OJQ)<br>*(highest vs lowest quartile)*<br>Relational injustice: OJQ *(highest vs lowest quartile)*	1.59<br><br>1.67<br><br>1.56	1.24-2.05<br><br>1.29-2.15<br><br>1.21-2.02
M- Hanson 2008 [2]<br>Sweden, various workers, N=3,004	Maslach Burnout Inventory-general Survey MBI-GS ≥ 75th percentile	Job demands: Swedish Work Environment Survey (SWES)<br>*(≥2 positive vs < 2 out of four items)*<br>Decision authority: SWES<br>*(≥2 positive vs < 2 out of four items)*<br>Co-worker support: SWES; one item<br>*(4 point Likert scale, dichotomized: low (1,2) vs high (3.4))*<br>Supervisor support: SWES; one item<br>*(4 point Likert scale dichotomized: low (1,2) vs high (3.4))*	♂ 2.09<br>♀ 1.79<br>♂ 1.36<br>♀ 1.41<br>♂ 1.45<br>♀ 1.92<br>♂ 1.65<br>♀ 1.22	1.52-2.88<br>1.36-2.35<br>0.98-1.88<br>1.07-1.86<br>0.97-2.17<br>1.25-2.93<br>1.19-2.31<br>0.91-1.65
Devereux 2011 [20]<br>England, 11 industrial types, N=1,463	General Health Questionnaire (GHQ-12)<br>GHQ-12 ≥ 3	Job demands questionnaire: 4 items<br>(*highest vs lowest tertile*)<br>Decision latitude: 15 items<br>(*lowest vs highest tertile*)<br>Co-worker and supervisor support: 7 items<br>(*lowest vs highest tertile*)	RR 1.62<br><br>RR 1.11<br><br>RR 1.47	1.26-2.09<br><br>0.86-1.42<br><br>1.18-1.84
Sundin 2011 [21]<br>Sweden, nurses, N=555	Maslach Burnout Inventory (MBI)<br>Swedish version, Emotional Exhaustion (EE)	Job demands: Swedish Work Environmental Survey (SWES), one item, 5 point Likert scale *(at least once a week or more vs ≤1 day out of 10)*<br>Co-worker support: SWES; one item, 4 point Likert scale	4.33<br><br>2.21	1.98-9.45<br><br>0.88-5.56

For peer review only

	EE ≥ 27	*(less (1,2,3) vs always receiving support (4))*<br>Supervisor support: SWES; one item, 4 point Likert scale<br>*(less (1,2,3) vs always receiving support (4))*	 2.17	 0.65-7.26
Inoue 2013 [3]<br>Japan, manufacturing, N=569	Kessler Psychological Distress Scale (K6)<br>≥5 K6 scale	Procedural injustice: Organizational Justice Questionnaire (OJQ): 7 items, 5 point scale; Permanent (P) non-permanent worker (NP)<br>*(highest vs lowest tertile)*<br>Relational injustice: OJQ interactional justice 6-items, 5 point scale<br>*(highest vs lowest tertile)*	♂ 1.37 (P)<br>♀ 5.25 (P)<br>♀ 2.84 (NP)<br>♂ 2.39 (P)<br>♀ 2.35 (P)<br>♀ 1.61 (NP)	0.43-4.34<br>0.82-33.6<br>1.19-6.75<br>0.61-9.39<br>0.51-10.9<br>0.78-3.30
Laine 2014 [22]<br>Finland, public sector, N=3,298	General Health Questionnaire GHQ-12<br>≥3 GHQ-12	Procedural injustice: 4 items Moorman's inventory<br>*(highest vs lowest quartile)*<br>Relational injustice: 4 items Moorman's inventory<br>*(highest vs lowest quartile)*<br>Bullying: item about mental violence or workplace bullying *(yes vs no)*	1.65<br><br>1.29<br><br>1.57	1.21-2.24<br><br>0.97-1.72<br><br>0.97-2.53
Taniguchi 2015 [23]<br>Japan, elderly facilities, N=543	Brief Job Stress Questionnaire (BJSQ)<br>BJSQ > 13 (♂) or >12 (♀)	Bullying: Japanese Negative Acts Questionnaire (NAQ), person-related bullying (PR; 6 items), work-related bullying (WR; 3 items), sexual harassment (SH; 3 items), five point scale. *(≥1 item positive versus none)*	3.46 (PR)<br>2.85 (WR)<br>1.73 (SH)	1.49-8.05<br>0.61-13.26<br>0.98-3.08
Andersen 2017 [24]<br>Denmark, prison personnel, N=1741	Copenhagen Burnout Inventory (COPSOQ-CBI)<br>COPSOQ-CBI highest quartile	Copenhagen Psychosocial Questionnaire (COPSOQ), 5-point Likert-scale (*most exposed quartile vs least exposed three quartiles)*<br>Job demands: 4 items<br>Co-worker and supervisor support: 6 items<br>Emotional demands: 4 items<br>Effort-reward imbalance (low recognition): 3 items	 <br>1.61<br>1.31<br>1.46<br>1.33	 <br>1.21-2.16<br>0.98-1.76<br>1.06-2.01<br>0.98-1.80
Oshio 2017 [25]<br>Japan, 12 industrial types, N=7419	Kessler Psychological Distress Scale (K6)<br>K6 ≥ 5	Job demands: 5 items Japanese Job Content Questionnaire (JCQ), 4-point scale *(median-split method classifying high vs low)*<br>Procedural injustice: Japanese version of Organisational Justice Questionnaire (OJQ), 7 items, 5-point Likert scale<br>Relational injustice: Japanese version of Organisational Justice Questionnaire (OJQ), 6 items, 5-point Likert scale<br>Effort reward imbalance: Japanese version Effort-Reward Imbalance Questionnaire, effort (3 items), reward (7 items), 4-point scale	♂ 1.62<br>♀ 1.71<br>♂ 1.68<br>♀ 1.80<br>♂ 1.58<br>♀ 1.71<br>♂ 2.05<br>♀ 1.84	1.45-1.81<br>1.38-2.13<br>1.51-1.87<br>1.45-2.23<br>1.42-1.77<br>1.37-2.13<br>1.84-2.29<br>1.48-2.28
Kind 2018 [26]	Burnout screening scales (BOSS)	Workplace aggression: exposure to verbal and physical threats		

For peer review only - http://bmjopen.bmj.com/site/about/guidelines.xhtml

Switzerland, youth welfare, N=121	BOSS T-score ≥60	*Verbal aggression vs no aggression*	HR 1.67	1.09-2.58
P-Thingvad 2019 [27] Denmark, social educators, N=1823	Copenhagen Burnout Inventory (CBI) CBI ≥ 75th percentile	Workplace violence: Scandinavian checklist, 4 items, 4-point Likert-scale *(high exposed (≥1 incidents per month) vs non- exposed)*	1.4	0.9-2.3

For peer review only

**Table 2. Methodological quality scores of 9 items for studies regarding risk factors***										
**Author**	**1**	**2**	**3**	**4**	**5**	**6**	**7**	**8**	**9**	**Summary score**
Mino 1999	-	+	-	-	+	+	+	- [a]	+	5
Stansfeld 1999	-	+	+	-	+	+	+	- [b]	+	6
Bϋltmann 2002	+	-	+	+	-	+	+	- [c]	+	6
Bonde 2005	+	+	+	-	+	+	+	+[d]	+	8
Godin 2005	+	-	+	+	-	+	+	- [e]	+	6
Kivimaki 10-town study 2007	+	+	+	-	-	+	+	- [f]	+	6
Kivimaki hospital study 2007	+	+	+	+	-	+	+	- [f]	+	7
Magnusson Hanson 2008	+	+	-	-	-	+	+	- [g]	+	5
Devereux 2011	+	?	-	+	-	+	+	- [h]	+	5
Sundin 2011	+	+	-	+	-	+	+	+[i]	+	7
Inoue 2013	+	+	+	+	-	+	+	+[j]	+	8
Laine 2014	-	+	+	+	-	+	+	+[k]	+	7
Taniguchi 2015	+	+	-	+	-	+	+	+[l]	+	7
Andersen 2017	+	+	-	+	-	+	+	+[m]	+	7
Oshio 2017	+	+	+	+	-	+	+	+[n]	+	8
Kind 2018	+	-	+	+	+	+	+	+[0]	+	8
Pihl-Thingvad 2019	+	+	+	+	-	+	+	+[p]	+	8
**Total item score**	14	13	11	12	4	17	17	9	17	

* Criteria and scoring options for quality score for these 9 items are reported in the methods section

[a]Adjusted for age, sex, family life satisfaction, perceived physical health

[b]Adjusted for age, sex, employment grade and baseline GHQ score

[c]Adjusted for age, sex, education, living alone, employment status, presence of disease, baseline fatigue score

[d]Adjusted for age, sex, body mass index, leisure time activity, pain threshold, marital status, psychiatric disorder

[e]Adjusted for age, sex, education, threat from global economy, job dissatisfaction, workplace instability

[f]Adjusted for age, sex, occupational status

[g]Adjusted for age, sex, marital status, country of birth, social class, physical exhaustion

[h]Adjusted for age, sex, shift work

[i]Adjusted for age, sex, marital status, years of (current) employment (only for job demands)

[j]Adjusted for age, sex, education, marital status, chronic physical diseases, occupation, life events, neuroticism

[k]Adjusted for age, sex, socioeconomic position, marital status, employment, health behaviour, limiting longstanding illness, physical work

[l]Adjusted for age, sex, job carrier, occupation, marital status, employment, work shift, smoking status

[m]Adjusted for age, sex, marital status, employment years, occupational characteristics and exposures

[n]Adjusted for age, sex, education, occupation, hours worked per week, household income, family member to share living expenses, firm codes

[o]Adjusted for age, sex, work experience, employment years, private stressors

[p]Adjusted for age, sex, somatic and mental health at baseline, lifestyle factors, work-related factors

For peer review only

Table 3. Quality of the evidence for the relationship between risk factors and stress related disorders according to the GRADE framework

	Number of participants	Number of cases	Number of studies	Study phase<br><br>Phase 1: ↓	Study limitations<br><br>Study quality majority of studies < 11/16: ↓	Inconsistency<br><br>I^2 >50% or 1 study or heterogeneity in definitions: ↓	Indirectness<br><br>Yes: ↓	Imprecision<br><br>Confidence interval (CI) effect size (<1 and >2, range >2) Yes: ↓	Publication Bias<br><br>Yes or unclear: ↓	Effect size OR (95% CI)<br><br>Lower Limit OR ≥ 2.0 : ↑	Exposure-response gradient (dose-effect)<br><br>Majority of studies: ↑	Overall Quality of Evidence
Effort-reward imbalance	76,760		6	2	no	48%	no	no	unclear↓	1.91 (1.70-2.15)	2/6	Moderate
Low procedural justice	64,676		5	2	no	0%	no	no	unclear↓	1.74 (1.62-1.86)	2/5	Moderate
Low relational justice	64,676		5	2	no	0%	no	no	unclear↓	1.55 (1.44-1.67)	2/5	Moderate
High job demands	41,397		8	2	no	49%	no	no	unclear↓	1.56 (1.41-1.72)	0/8	Moderate
Low co-worker support	22,920		4	2	no	10%	no	no	unclear↓	1.29 (1.17-1.43)	0/4	Moderate
Low supervisor support	23,382		5	2	no	0%	no	no	unclear↓	1.27 (1.16-1.38)	0/5	Moderate
Low co-worker and supervisor support	7,262		2	2	no	0%	no	no	unclear↓	1.41 (1.18-1.69)	0/2	Moderate
High emotional demands	13,641		2	2	no	0%	no	no	unclear↓	1.58 (1.35-1.84)	0/2	Moderate
Low decision authority	13,312		2	2	no	0%	no	no	unclear↓	1.34 (1.20-1.49)	0/2	Moderate
Job insecurity	8,833		1	2	no	1 study ↓	no	no	unclear↓	Men 1.63 (1.18-2.27) Women 0.94 (0.56-1.59)	0/1	Low
Decision latitude	11,287		2	2	no	0%	no	no	unclear↓	1.07 (0.92-1.25)	0/2	Moderate
Low skill discretion	3,123		2	2	no	0%	no	no	unclear↓	1.11 (0.94-1.32)	0/2	Moderate
Bullying & violence	9,694		4	2	no	Heterogeneity in definitions ↓	no	yes: ↓	unclear↓	1.42 – 3.64 (0.42-15.92)	0/4	Very low

For peer review only - http://bmjopen.bmj.com/site/about/guidelines.xhtml

Methodological quality

The methodological quality of the studies of risk factors varied from scoring 5 out of 9 items to 8 out of 9 items (see table 2). The most frequently missing quality items were: response at follow-up less 70%, data on outcome were collected at least at three different time points, and study controls for confounding with rationale.

Meta-analyses and assessment of evidence

In total, twelve psychosocial risk factors from 14 studies [1-3, 15-22, 24,25] from a population of 73,874 workers from Belgium, Denmark, England, Finland, Japan, Netherlands and Sweden were used in the meta-analysis. Reported distress prevalence varied from 4.9% [17] to 45.8% [3]. The follow-up time varied from one to five years. Table 3 summarises the assessment of evidence concerning the psychosocial risk factors for stress-related disorders.

Effort-reward balance

Six cohort studies [16,18,19,24,25] demonstrate that there is moderate-quality evidence that effort-reward imbalance increases the incidence of SRD, with a pooled OR of 1.91 (95% CI 1.70-2.15) (see table 3 and figure 2.1). No statistically significant difference between subgroups of men and women were found [16,18,25].

Procedural and relational justice

Five cohort studies [3,19,22,25] demonstrate that there is moderate-quality evidence that low procedural justice with a pooled OR of 1.73 (95% CI 1.61-1.86) and low relational justice with a pooled OR of 1.55 (95%CI: 1.44-1.67) increase the incidence of SRD (see table 3 and figure 2.2). No statistically significant difference between subgroups of men and women were found [3,25].

For peer review only - http://bmjopen.bmj.com/site/about/guidelines.xhtml

Job demands

Eight cohort studies [1,2,15,16,20,21,24,25] demonstrate that there is moderate-quality evidence that high job demands increases the incidence of SRD, with a pooled OR of 1.55 (95% CI 1.41-1.71) (see table 3 and figure 2.3). No statistically significant difference between subgroups of men and women were found [1,2,15,16,25].

Co-worker and supervisor support

In total, five cohort studies demonstrate that there is moderate-quality evidence that low co-workers support [1,2,16,21] with a pooled OR 1.29 (95%CI: 1.17-1.43) and low supervisor support [1,2,15,16,21] with a pooled OR 1.27 (95%CI: 1.14-1.40) increase the incidence of SRD. No statistically significant difference between subgroups of men and women were found [1,215,16]. The combination of low co-worker and supervisor support [20,24] resulted in a pooled OR 1.41 (95%CI 1.18-1.69). (see table 3).

Emotional demands

Two cohort studies [1,24] demonstrate that there is moderate-quality evidence that high emotional demands increase the incidence of SRD, with a pooled OR of 1.58 (95% CI 1.35-1.84) (see table 3). No statistically significant difference between men and women were found in the study of Bültmann et al. 2002 [1].

Decision authority

Two cohort studies [2,16] demonstrate that there is moderate-quality evidence that low decision autonomy increases the incidence of SRD with a pooled OR of 1.34 (95% CI 1.20-

For peer review only - http://bmjopen.bmj.com/site/about/guidelines.xhtml

1.49) (see table 3). No statistically significant difference between subgroups of men and women were found.

Job security, decision latitude, skill discretion and bullying

One cohort study [1] demonstrates that there is low-quality evidence that job insecurity increases the incidence of SRD, with a pooled OR of 1.63 (95% CI 1.18-2.27) for male and no increase of the incidence for female with a pooled OR of 0.94 (95% CI 0.56-1.59). Two cohort studies [1,20] demonstrate that there is moderate-quality evidence for no increased incidence of SRD due to low decision latitude, with an OR of 1.07 (95% CI 0.92-1.25). One cohort study [17] demonstrates that there is moderate-quality evidence for no increased incidence of SRD due to low skill discretion, with an OR of 1.11 (95% CI 0.94-1.32). Four cohort studies [22,23,26,27] demonstrate very low-quality evidence that there is inconsistent evidence for increased incidence of SRD with different types of bullying and workplace violence with ORs varying from 1.42 (95%CI 0.42-4.79) to 3.64 (95%CI 0.83-15.92). (see table 3).

Figures 2.1 -2.3 about here

For peer review only - http://bmjopen.bmj.com/site/about/guidelines.xhtml

For peer review only

DISCUSSION

Main findings

This systematic review, including a meta-analysis of prospective cohort studies revealed moderate evidence that effort-reward imbalance, low procedural and relational justice, high job demands, low co-worker and supervisor support, high emotional demands, and low decision authority increase the incidence of stress-related disorders, varying from 20% to 90%. Low-quality evidence was found for an association between job insecurity and stress-related disorder among men. Moderate to very low-quality evidence suggesting no associations were found for decision latitude, skill discretion and bullying.

Psychosocial risk factors and SRD

All longitudinal studies were controlled for the personal factors of age and sex, and non-work-related factors such as education and marital status. Six studies also controlled for other work-related psychosocial risk factors in various models [3,19,20,22,27]. Regarding these studies, we decided not to take into account the fully adjusted model in the meta-analyses in order to avoid overcorrection, and strengthen transparency and comparability between studies. However, these models yielded small and non-statistical significant differences between the risk estimates.

All studies defined SRD based on self-report with validated and standardised questionnaires, i.e. (shortened) GHQ [1,15,16,19,20,22], Kessler 6 [3,25], MBI [2,21], COPSOQ-CBI [24,27], Setterlind Stress Inventory Profile [17], fatigue inventory [18], Brief job Stress Questionnaire (BJSQ) [27], burnout screening scale (BOSS) [26].

The pooled ORs of the meta-analyses of psychosocial risk factors were equal or slightly raised compared with the prior results of Nieuwenhuijsen et al. [10] and in line with the findings of a

For peer review only - http://bmjopen.bmj.com/site/about/guidelines.xhtml

recent meta-review of work-related risk factors for common mental-health problems including depression and anxiety [11].

Methodological considerations

The exposure categories for the risk factors and the case definitions of SRD varied in the included studies. We have explicitly described all definitions and assessments of self-reported exposures and outcomes (see table 1). In general, psychological distress can stem from private life as well as from work. Therefore we only included high quality prospective studies aimed at identifying work-related risk factors, also controlling for other factors outside the work context. Although there is debate about the definition of burnout [5,7, 28], we regarded distress and burnout as outcomes of the same continuum. We found that similar work-related psychosocial risk factors were associated with distress and the one-dimensional measures of burn-out ('exhaustion'). Should more studies using the multiple dimension definition of burnout become available, then subgroup analyses of these studies can be performed to assess whether the work-related risk factors are similar in this diagnostic subgroup.

We used the risk estimates as reported in the original longitudinal studies (see also table 1; i.e. OR, RR and HR). Mostly they reported ORs, possibly because of the mathematical advantages of using OR. Meta-analyses were performed to estimate pooled associations, although the variety in measurement instruments and dichotomization rules introduced heterogeneity in the outcome measures (case definitions) and exposure categories of psychosocial risk factors. However, the exposure categories for the psychosocial risk factors included in the meta-analyses were already established in theoretical models of effort-reward imbalance [29], job demands-control [30] and organisational justice [31]. Furthermore, the case definitions of SRD used validated questionnaires and the methodological quality of the included studies was high and accompanied by a low to moderate statistical heterogeneity across the pooled studies (I^2

For peer review only - http://bmjopen.bmj.com/site/about/guidelines.xhtml

varying from 0% to 50%) which justify the meta-analyses. With the low number of studies for each risk factor we feel that more subgroup analyses – besides the meta-analyses for separate risk factors and sexes – are not informative.

The GRADE framework made it possible to assess to the quality of evidence in reviews of prognostic factor research. Only publication bias was difficult to assess because of the small number of studies included on each risk factor; however, we treated the assessment of publication bias conservatively by downgrading the evidence in the case of lack of clarity. For the risk factor, bullying and violence, it was decided not to perform a meta-analysis because of the different concepts of the psychosocial risk factor, i.e. person-related bullying, work-related bullying, sexual harassment, workplace bullying, threatening or violence.

Eventually, further research in harmonizing the assessment of SRD in cohort studies [7] and the clinical assessment with signs and symptoms of SRD [32], combined with more detailed or objective task-based exposure assessment [33] will contribute to a better insight into the work-related psychosocial risk factors and their mediating or moderating factors.

Prevention

To manage and prevent stress disorders it is necessary to identify which psychosocial risk factors at work contribute to the onset of SRD. West et al. [34] describe the importance of organisational strategies in their review on interventions to prevent and reduce SRD among physicians. Kalani et al. [35] report in their meta-review that various studies of the effectiveness of individually and organisationally directed interventions have led to different results on reducing physician burnout. Ultimately, to develop individually and organisationally directed interventions it is necessary that the relevant psychosocial risk factors in organisations are known [34,35].

For peer review only

For peer review only - http://bmjopen.bmj.com/site/about/guidelines.xhtml

Recently, Fan et al. [36] has suggested that some psychosocial factors such as job control, job security and social support are also associated with a greater likelihood of workers' experiencing positive mental well-being in terms of satisfaction and purpose in life, personal growth, social contribution and integration. This study implicates the double value of workplace policies and practices that improve psychosocial working conditions, reduce work-related SRD and improve mental well-being in general, e.g. by giving workers greater job control or social support [36].

In conclusion, several psychosocial work-related risk factors for SRD were established, confirming the multifactorial aetiology of SRD. Effort-reward imbalance, low organisational justice and high job demands were associated with the largest increased risk of SRD, varying from 60 to 90%. Awareness of these risk factors could be the starting point for the selection of preventive interventions to reduce work-related SRD.

For peer review only

Acknowledgement

We are very grateful to Joost Daams for his contribution to the literature search and Nicolaas Kylstra and Kim van der Molen for their help with screening activities.

Competing interests

The authors declare that they have no competing interests.

Contributors

HM and GG initiated the study, performed data extraction and statistical analyses, and drafted the manuscript. All authors performed the study selection and interpretation of the data. All

For peer review only - http://bmjopen.bmj.com/site/about/guidelines.xhtml

authors made substantial contributions to the conception of the study and manuscript. HM and GG equally contributed.

Funding

The work was sponsored by the Ministry of Social Affairs and Employment, the Netherlands. Grant number: 5100-24108

Competing interests None declared.

Patient consent for publication Not required.

Provenance and peer review Not commissioned; externally peer reviewed.

Data sharing statement No additional data available.

For peer review only

References

1. Bultmann U, Kant IJ, van den Brandt PA et al. Psychosocial work characteristics as risk factors for the onset of fatigue and psychological distress: prospective results from the Maastricht Cohort Study. *Psychol Med* 2002;32:333-345.
2. Magnusson Hanson LL, Theorell T, Oxenstierna G, Hyde M, Westerlund G. Demand, control and social climate as predictors of emotional exhaustion symptoms in working Swedish men and women. *Scand J Public Health* 2008; 36: 737-743.
3. Inoue A, Kawakami N, Tsuno K, Tomioka K, Nakanishi M. Organizational justice and psychological distress among permanent and non-permanent employees in Japan: a prospective cohort study. *Int J Behav Med* 2013; 20: 265-276.
4. van der Molen HF, de Vries S, Sluiter JK. Occupational diseases among workers in lower and higher socioeconomic positions. *Int J Environ Res Public Health* 2018;15(12):2849.
5. WHO. Burn-out an "occupational phenomenon": International Classification of DiseasesWhat are social determinants of health? 2019 https://www.who.int/mental_health/evidence/burn-out/en/?mc_cid=c8878361fa&mc_eid=8d4606394a (accessed 6 Aug 2019).
6. Lastovkova A, Cardner M, Rasmussen HM et al. Burnout syndrome as an occupational disease in the European Union: an exploratory study. *Ind Health* 2018;56(2):160-165.
7. Canu I, Bianchi R, Bubas M et al. Which factors contribute to the onset of burnout among workers? A systematic review. PROSPERO 2018 CRD42018105901 Available from: http://www.crd.york.ac.uk/PROSPERO/display_record.php?ID=CRD42018105901
8. Chirico F. Is burnout a syndrome or an occupational disease? Instructions for occupational physicians *Epidemiol Prev* 2017;41 (5-6):294-298.
9. Chirico F. The forgotten realm of the new and emerging psychosocial risk factors. *J Occup Health* 2017; 59(5):433-435.
10. Nieuwenhuijsen K, Bruinvels D, Frings-Dresen M. Psychosocial work environment and stress-related disorders, a systematic review. *Occup Med* (Lond) 2010;60:277-286
11. Harvey SB, Modini M, Joyce S et al. Can work make you mentally ill? A systematic meta-review of work-related risk factor for common mental health problems. *Occup Environ Med* 2017;74:301-310.
12. Milner A, Witt K, LaMontagne AD et al. Psychosocial job stressors and suicidality: a meta-analysis and systematic review. *Occup Environ Med* 2018;75:245-253.
13. Watanabe K, Imamura K, Kawakami N. Working hours and the onset of depressive disorder: a systematic review and meta-analysis. *Occup Environ Med* 2016;73(12):877-884.
14. Huguet A, Hayden J, Stinson J, McGrath P, Chambers C, Tougas M, Wozney L. Judging the quality of evidence in reviews of prognostic factor research: adapting the GRADE framework. *Syst Rev* 2013;2:71.
15. Mino Y, Shigemi J, Tsuda T et al. Perceived job stress and mental health in precision machine workers of Japan: a 2 year cohort study. *Occup Environ Med* 1999;56:41-45.
16. Stansfeld SA, Fuhrer R, Shipley MJ et al. Work characteristics predict psychiatric disorder: prospective results from the Whitehall II Study. *Occup Environ Med* 1999;56:302-307.
17. Bonde JP, Mikkelsen S, Andersen JH et al. Understanding work related musculoskeletal pain: does repetitive work cause stress symptoms? *Occup Environ Med* 2005;62:41-48.
18. Godin I, Kittel F, Coppieters Y et al. A prospective study of cumulative job stress in relation to mental health. *BMC Public Health* 2005;5:67.
19. Kivimaki M, Vahtera J, Elovainio M et al. Effort-reward imbalance, procedural injustice and relational injustice as psychosocial predictors of health: complementary or redundant models? *Occup Environ Med* 2007;64:659–665.

For peer review only - http://bmjopen.bmj.com/site/about/guidelines.xhtml

20. Devereux JJ, Rydstedt LW, Cropley M. Psychosocial work characteristics, need for recovery and musculoskeletal problems predict psychological distress in a sample of British Workers. *Ergonomics* 2011; 54(9),840-848.
21. Sundin L, Hochwälder J, Lisspers J. A longitudinal examination of generic and occupational specific job demands, and work-related social support associated with burnout among nurses in Sweden. *Work* 2011;38: 389-400.
22. Laine H, Saastamoinen P, Lahti J, Rahkonen O, Lahelma E. The associations between psychosocial working conditions and changes in common mental disorders: a follow-up study. *BMC Public Health* 2014;14-588.
23. Taniguchi T, Takaki J, Hirokawa K, Fujii Y, Harano K. Associations of workplace bullying and harassment with stress reactions: a two year follow-up study. *Ind Health* 2016,54,131-138.
24. Andersen D, Andersen L, Gadegaard C, Hogh A, Prieur A, Lund T. Burnout among Danish prison personnel: a Question of quantitative and emotional demands. *Scand J Public Health* 2017;45:824-830.
25. Oshio T, Inoue A, Tsutsumi A. Examining the mediating effect of work-to-family conflict on the associations between job stressors and employee psychological distress: a prospective cohort study. *BMJ Open* 2017;7:e015608.
26. Kind N, Eckert A, Steinlinc C, Fegert JM, Schmid M. Verbal and physical client aggression – A longitudinal analysis of professional caregivers' psychophysiological stress response and burnout. *Psychoneuroendocrinology* 2018;94:11-16.
27. Pihl-Thingvad J, Elklit A, Brandt LPA, Andersen LL. Workplace violence and development of burnout symptoms: a prospective cohort study on 1823 social educators. *Int Arch Occ Env Hea* 2019;92:843-853.
28. Maslach C, Leiter MP. Understanding the burnout experience: recent research and its implications for psychiatry. *World Psychiatry* 2016;15(2):103-111.
29. Siegrist J, Siegrist K, Weber I. Sociological concepts in the etiology of chronic disease: the case of ischemic heart disease. *Soc Sci Med* 1986;22:247-253.
30. Karasek RA, Jr. Job demands, job decision latitude, and mental strain: implications for job design. *Admin Sci Q* 1979;24:285-308.
31. Moorman RH. Relationship between organizational justice and organizational citizenship behaviors: do fairness perceptions influence employee citizenship? *J Appl Psychol* 1991;76:845-855.
32. Kakiashvili T, Leszek J, Rutkowski K The medical perspective on burnout. *Int J Occup Med Environ Health* 2013;26(3):401-12.
33. Göras C Olin K Unbeck M Pukk-Härenstam K. Tasks, multitasking and interruptions among the surgical team in an operating room: a prospective observational study. *BMJ Open* 2019;9(5):e026410.
34. West C, Dyrbye L, Erwin P, Shanafelt T. Interventions to prevent and reduce physician burnout: a systematic review and meta-analysis. *Lancet* 2016;388(10057):2272-2281.
35. Kalani S, Azadfallah P, Oreyzi H, Adibi P. Interventions for Physician Burnout: a systematic review of systematic reviews. *Int J Prev Med* 2018;9:81.
36. Fan JK, Mustard C, Smith PM. Psychosocial work conditions and mental health: examining differences across mental illness and well-being outcomes. *Annals of Work Exposures and Health* 2019;63 (5):546-559.

For peer review only - http://bmjopen.bmj.com/site/about/guidelines.xhtml

Figures, Tables & appendix

Figures

Figure 1 Prisma Flow Diagram

Figures 2.1 – 2.3 Forest Plots

Tables

Table 1. Associations between psychosocial risk factors at work and the occurrence of stress-related disorders (17 studies)

Table 2 Methodological quality scores of the studies including those concerning psychosocial risk factors

Table 3 Quality of the evidence of the relationship between psychosocial risk factors and stress-related disorders according to the GRADE framework

Appendix

Search of Medline, Embase and PsychInfo for studies between 2008 and 12 August 2019

For peer review only - http://bmjopen.bmj.com/site/about/guidelines.xhtml

Figure 1 PRISMA Flow Diagram

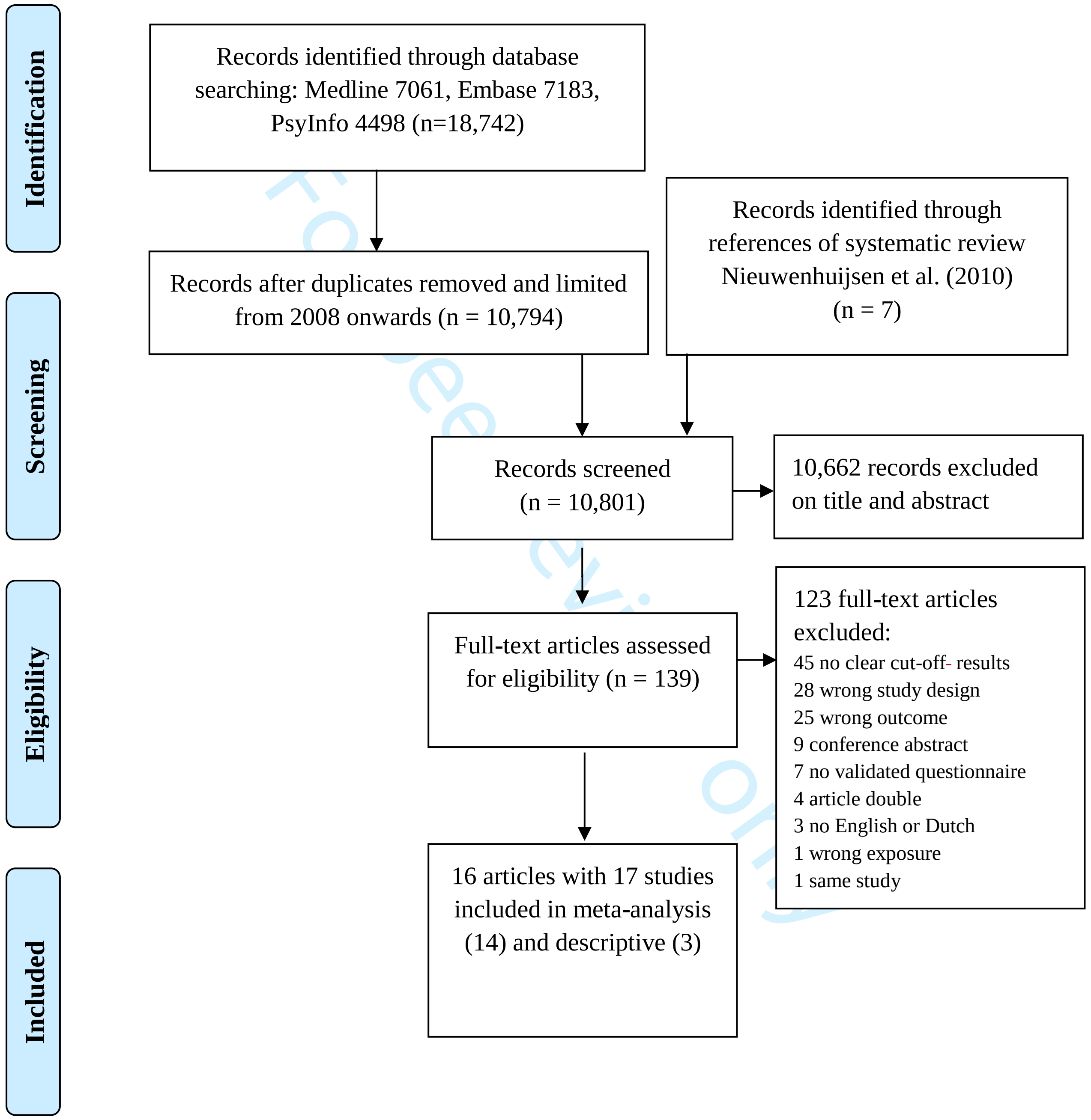

For peer review only - http://bmjopen.bmj.com/site/about/guidelines.xhtml

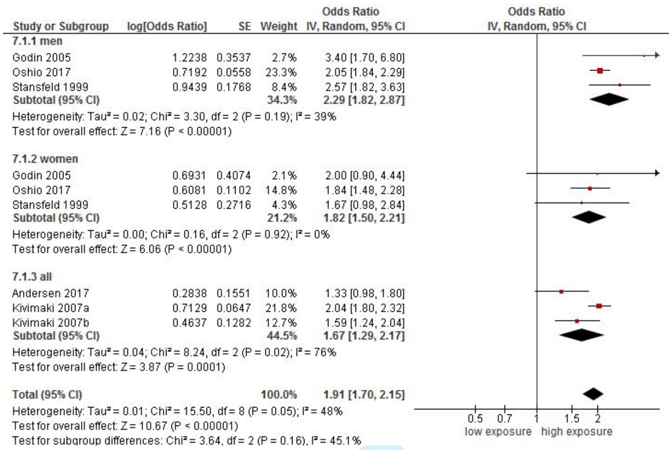

Fig 2.1 Forest plot of studies regarding effort-reward imbalance and SRD

For peer review only - http://bmjopen.bmj.com/site/about/guidelines.xhtml

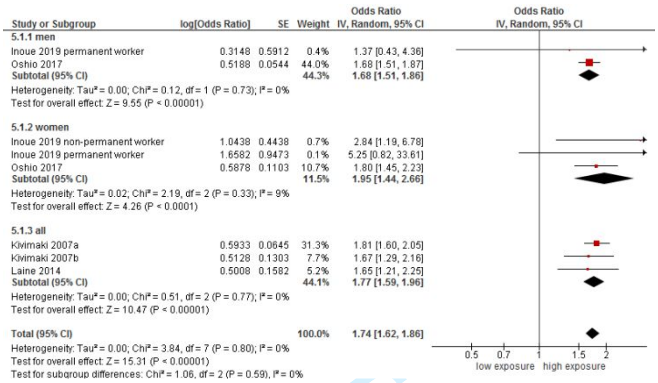

Fig 2.2 Forest plot of studies regarding procedural injustice and SRD

For peer review only - http://bmjopen.bmj.com/site/about/guidelines.xhtml

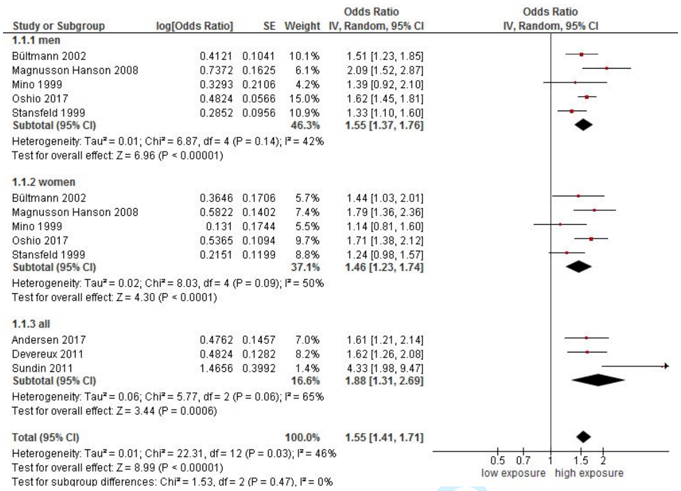

Fig 2.3 Forest plot of studies regarding job demands and SRD

For peer review only - http://bmjopen.bmj.com/site/about/guidelines.xhtml

PRISMA 2009 Checklist

Section/topic	#	Checklist item	Reported on page #
**TITLE**			
Title	1	Identify the report as a systematic review, meta-analysis, or both.	1
**ABSTRACT**			
Structured summary	2	Provide a structured summary including, as applicable: background; objectives; data sources; study eligibility criteria, participants, and interventions; study appraisal and synthesis methods; results; limitations; conclusions and implications of key findings; systematic review registration number.	2
**INTRODUCTION**			
Rationale	3	Describe the rationale for the review in the context of what is already known.	3,4
Objectives	4	Provide an explicit statement of questions being addressed with reference to participants, interventions, comparisons, outcomes, and study design (PICOS).	4
**METHODS**			
Protocol and registration	5	Indicate if a review protocol exists, if and where it can be accessed (e.g., Web address), and, if available, provide registration information including registration number.	n/a
Eligibility criteria	6	Specify study characteristics (e.g., PICOS, length of follow-up) and report characteristics (e.g., years considered, language, publication status) used as criteria for eligibility, giving rationale.	4,5
Information sources	7	Describe all information sources (e.g., databases with dates of coverage, contact with study authors to identify additional studies) in the search and date last searched.	5
Search	8	Present full electronic search strategy for at least one database, including any limits used, such that it could be repeated.	Appendix 1
Study selection	9	State the process for selecting studies (i.e., screening, eligibility, included in systematic review, and, if applicable, included in the meta-analysis).	5,6
Data collection process	10	Describe method of data extraction from reports (e.g., piloted forms, independently, in duplicate) and any processes for obtaining and confirming data from investigators.	6
Data items	11	List and define all variables for which data were sought (e.g., PICOS, funding sources) and any assumptions and simplifications made.	5,6
Risk of bias in individual studies	12	Describe methods used for assessing risk of bias of individual studies (including specification of whether this was done at the study or outcome level), and how this information is to be used in any data synthesis.	6
Summary measures	13	State the principal summary measures (e.g., risk ratio, difference in means).	7
Synthesis of results	14	Describe the methods of handling data and combining results of studies, if done, including measures of consistency (e.g., I^2) for each meta-analysis.	7,8

For peer review only

PRISMA 2009 Checklist

Section/topic	#	Checklist item	Reported on page #
Risk of bias across studies	15	Specify any assessment of risk of bias that may affect the cumulative evidence (e.g., publication bias, selective reporting within studies).	7,8
Additional analyses	16	Describe methods of additional analyses (e.g., sensitivity or subgroup analyses, meta-regression), if done, indicating which were pre-specified.	n/a
**RESULTS**			
Study selection	17	Give numbers of studies screened, assessed for eligibility, and included in the review, with reasons for exclusions at each stage, ideally with a flow diagram.	8<br>Figure 1
Study characteristics	18	For each study, present characteristics for which data were extracted (e.g., study size, PICOS, follow-up period) and provide the citations.	Table 1
Risk of bias within studies	19	Present data on risk of bias of each study and, if available, any outcome level assessment (see item 12).	Table 2
Results of individual studies	20	For all outcomes considered (benefits or harms), present, for each study: (a) simple summary data for each intervention group (b) effect estimates and confidence intervals, ideally with a forest plot.	Table 1
Synthesis of results	21	Present results of each meta-analysis done, including confidence intervals and measures of consistency.	16-18<br>Figure 2.1-2.3
Risk of bias across studies	22	Present results of any assessment of risk of bias across studies (see Item 15).	Table 3
Additional analysis	23	Give results of additional analyses, if done (e.g., sensitivity or subgroup analyses, meta-regression [see Item 16]).	n.a.
**DISCUSSION**			
Summary of evidence	24	Summarize the main findings including the strength of evidence for each main outcome; consider their relevance to key groups (e.g., healthcare providers, users, and policy makers).	19,20
Limitations	25	Discuss limitations at study and outcome level (e.g., risk of bias), and at review-level (e.g., incomplete retrieval of identified research, reporting bias).	20,21
Conclusions	26	Provide a general interpretation of the results in the context of other evidence, and implications for future research.	21
**FUNDING**			
Funding	27	Describe sources of funding for the systematic review and other support (e.g., supply of data); role of funders for the systematic review.	23

From: Moher D, Liberati A, Tetzlaff J, Altman DG, The PRISMA Group (2009). Preferred Reporting Items for Systematic Reviews and Meta-Analyses: The PRISMA Statement. PLoS Med 6(7): e1000097. doi:10.1371/journal.pmed1000097

For peer review only - http://bmjopen.bmj.com/site/about/guidelines.xhtml

BMJ Open

Work-related psychosocial risk factors for stress-related mental disorders – an updated systematic review and meta-analysis

Journal:	*BMJ Open*
Manuscript ID	bmjopen-2019-034849.R1
Article Type:	Original research
Date Submitted by the Author:	06-Mar-2020

Complete List of Authors:	van der Molen, Henk; Amsterdam UMC - Locatie AMC, Coronel Institute of Occupational Health<br>Nieuwenhuijsen, Karen; Amsterdam UMC - Locatie AMC, Coronel Institute of Occupational Health<br>Frings-Dresen, Monique; Amsterdam UMC - Locatie AMC, Coronel Institute of Occupational Health<br>de Groene, Gerda; Amsterdam UMC - Locatie AMC, Coronel Institute of Occupational Health

**Primary Subject Heading**:	Occupational and environmental medicine
Secondary Subject Heading:	Epidemiology
Keywords:	OCCUPATIONAL & INDUSTRIAL MEDICINE, EPIDEMIOLOGY, PREVENTIVE MEDICINE

SCHOLARONE™
Manuscripts

I, the Submitting Author has the right to grant and does grant on behalf of all authors of the Work (as defined in the below author licence), an exclusive licence and/or a non-exclusive licence for contributions from authors who are: i) UK Crown employees; ii) where BMJ has agreed a CC-BY licence shall apply, and/or iii) in accordance with the terms applicable for US Federal Government officers or employees acting as part of their official duties; on a worldwide, perpetual, irrevocable, royalty-free basis to BMJ Publishing Group Ltd ("BMJ") its licensees and where the relevant Journal is co-owned by BMJ to the co-owners of the Journal, to publish the Work in this journal and any other BMJ products and to exploit all rights, as set out in our licence.

The Submitting Author accepts and understands that any supply made under these terms is made by BMJ to the Submitting Author unless you are acting as an employee on behalf of your employer or a postgraduate student of an affiliated institution which is paying any applicable article publishing charge ("APC") for Open Access articles. Where the Submitting Author wishes to make the Work available on an Open Access basis (and intends to pay the relevant APC), the terms of reuse of such Open Access shall be governed by a Creative Commons licence – details of these licences and which Creative Commons licence will apply to this Work are set out in our licence referred to above.

Other than as permitted in any relevant BMJ Author's Self Archiving Policies, I confirm this Work has not been accepted for publication elsewhere, is not being considered for publication elsewhere and does not duplicate material already published. I confirm all authors consent to publication of this Work and authorise the granting of this licence.

For peer review only

Work-related psychosocial risk factors for stress-related mental disorders – an updated systematic review and meta-analysis

Henk F. van der Molen[1], Karen Nieuwenhuijsen[1], Monique H.W. Frings-Dresen[1], Gerda de Groene[1]

[1]Amsterdam UMC, University of Amsterdam, Department: Coronel Institute of Occupational Health, University of Amsterdam, Netherlands Center for Occupational Diseases, Amsterdam Public Health research institute, PO Box 22660, 1100 DD Amsterdam, the Netherlands

[2] Corresponding author
h.f.vandermolen@amsterdamumc.nl

For peer review only

For peer review only - http://bmjopen.bmj.com/site/about/guidelines.xhtml

Abstract

Objective The objective was to conduct an update of a previously published review and meta-analysis on the association between work-related psychosocial risk factors and stress-related mental disorders (SRD).

Design Systematic review and meta-analysis

Data sources Medline, Embase and PsycINFO were searched for articles published between 2008 and 12 August 2019 and references of a systematic review performed for the period before 2008 were included. Primary prospective studies were included when outcome data were described in terms of SRD assessment or a dichotomous outcome, based on a validated questionnaire, and at least two levels of work-related exposure were reported (exposed versus less or non-exposed). We used GRADE to assess the evidence for the associations between risk factors and the onset of SRD.

Results

Seventeen studies met the inclusion criteria. In total, a population of 73,874 workers from Belgium, Denmark, England, Finland, Japan, the Netherlands and Sweden were included in the meta-analysis of fourteen prospective cohort studies. This meta-analysis revealed moderate evidence for associations between SRD and effort reward imbalance (OR=1.9, 95% CI 1.70-2.15), high job demands (OR=1.6, 95% CI 1.41-1.72), organisational justice (OR's 1.6 to 1.7, CI's 1.44-1.86), social support (OR's 1.3 to 1.4, CI's 1.16-1.69), high emotional demands (OR=1.6, 95% CI 1.35-1.84), and decision authority (OR=1.3, CI 1.20-1.49). No significant or inconsistent associations were found for job insecurity, decision latitude, skill discretion and bullying.

Conclusion

Moderate evidence was found that work-related psychosocial risk factors are associated with a higher risk of SRD. Effort-reward imbalance, low organisational justice and high job demands exhibited the largest increased risk of SRD, varying from 60 to 90%.

Keywords

Mental, Stress Related Disorders, Work, Risk Factors, Meta-analysis, Aetiology

For peer review only

Strengths and limitations of this study

► Stress related mental disorders are frequently reported in the working population, however the work attribution is often not assessed.

► This systematic review, including a meta-analysis of prospective cohort studies, gives a valuable overview which work-related psychosocial risk factors increase the risk of stress related mental disorders.

► The identified psychosocial work-related risk factors are the starting point for evidence based universal and selective preventive measures to reduce work-related stress mental disorders.

► Limitations are the lack of harmonized assessments of stress related mental disorders and detailed exposure assessment in the identified observational cohort studies.

For peer review only

For peer review only - http://bmjopen.bmj.com/site/about/guidelines.xhtml

Introduction

Stress related mental disorders (SRD) are frequently reported in the working population, with varying incidence rates of 13% for psychological distress [1], 22% for emotional exhaustion [2], and a prevalence of distress symptoms of up to 50% in specific professions and countries [3]. SRD represent a significant part of work-related common mental disorders in both self-report surveys and notification systems for occupational diseases [4]. The World Health Organization has recently announced [5] that burn-out is included as 'an occupational phenomenon' in the 11th revision of the International Classification of Diseases (ICD-11).

In many countries, SRD are not notified as occupational diseases [6], mainly because of their multifactorial origin [7] or discussion about the medical status (as with burn-out) [5,8,9]. Evidence-based knowledge about work-related factors could be used for facilitating a decision on the 'work-relatedness' of an individual case of SRD in a reporting scheme or concerning the selection and implementation of preventive workplace interventions around factors with the strongest effect on SRD [7].

In the Netherlands, SRD cover diagnoses of adjustment disorders (ICD-10: F43.2) and burn-out as a state of exhaustion (ICD-10: Z730) [4]. These diagnoses differ from just stress symptoms since they are clinically assessed and have in common that they reflect a status where subjective and emotional disturbance interfere with social functioning and performance, arising from a period of adaptation to stressful conditions. Symptoms are what a worker experiences, and these symptoms might negatively affect normal or regular functioning of a person – a so called disorder. To distinguish between symptoms and disorders, we considered a SRD as diagnosis following a clinical anamnesis, stress complaints as assessed in a score above a cut-off point on a validated questionnaire or absenteeism from work due to stress problems. Work-related psychosocial risk factors can contribute to the onset of SRD [10].

For peer review only - http://bmjopen.bmj.com/site/about/guidelines.xhtml

The high and increasing burden of SRD strengthens the urgency of up-to-date insight into work-related psychosocial risk factors associated with SRD and based on longitudinal study designs in order to initiate relevant primary and secondary preventive interventions at worksites. Previous systematic reviews aiming to determine work-related risk factors for SRD have either not been updated since 2008 [10], combined various common mental disorders, such as anxiety, depression and stress-related disorders, as outcome measures [11] or used other outcomes, such as suicide [12]. Based on seven prospective studies, Nieuwenhuijsen et al [10] reported that high job demands, low job control, low co-worker support, low supervisor support, low procedural justice, low relational justice, high effort–reward imbalance and job insecurity (only for men) predicted the occurrence of SRDs. Our aim is to update this previously published review and meta-analysis from 2008 [10] to examine: (1) which work-related psychosocial risk factors contribute to the onset of SRD and (2) to what extent these risk factors are associated with SRD.

Methods

This review followed the PRISMA Statement (see appendix). This review was not registered in Prospero, but an update of a previous review by Nieuwenhuijsen et al. [10].

Patient and public involvement

We analysed published cohort studies and did not seek information from individuals. In other words, there was no patients or public involvement in the study.

Study selection

Eligibility criteria

For peer review only - http://bmjopen.bmj.com/site/about/guidelines.xhtml

Primary prospective cohort studies were included where outcome data were described in terms of clinically or questionnaire-assessed stress-related disorder (present or not) and at least two levels of work-related exposure (exposed versus less or non-exposed) among a working population were reported in order to be able to retrieve or calculate a risk estimate (OR, RR, or HR).

This updated systematic review [10] considered studies to have included SRD if the outcome was either i) a SRD diagnosis following a clinical anamnesis, ii) a high level of stress complaints as assessed in a score above a cut-off point on a validated questionnaire for fatigue, stress or non-specific mental ill-health, or iii) absenteeism from work due to stress problems. Based on findings of Nieuwenhuijsen et al [10] and identified factors stemming from theoretical stress models, we defined seven types of exposure (i) job demands, (ii) job control (decision latitude, decision authority, skill discretion), (iii) social support (co-worker, supervisor, both), (iv) emotional demands, v) organisational justice (procedural and relational), (vi) effort-reward imbalance and (vii) other psychosocial risk factors, e.g. bullying.

Studies that described work-related risk factors in terms of psychosocial risk factors were eligible for inclusion. The studies had to describe workers in a real workplace setting. All types of exposure assessment were eligible for inclusion: self-reports or researcher observations or direct measurements. No additional criteria were formulated regarding latency between exposure and the presence or onset of the disorder or adjustment for confounders.

Data sources and search terms

We searched the electronic databases of Medline, Embase and PsychInfo for studies between 2008 and 12 August 2019 as described in supplement Appendix 1. Our PICO can be stated as:

For peer review only - http://bmjopen.bmj.com/site/about/guidelines.xhtml

P = working population, I/C exposed / less or none exposed to a-priori defined exposure categories, O = stress related disorder. Eligible studies before 2008 were retrieved from the systematic review by Nieuwenhuijsen et al. [10].

Data collection and analyses

Study selection process

Titles and abstracts were independently screened by two reviewers to identify potentially relevant studies. We used a free online software tool to screen and assess references (https://rayyan.qcri.org/welcome). The full texts of potentially relevant articles were assessed for eligibility against the inclusion criteria. Disagreement between review authors on the selection of studies for inclusion occurred in less than 5% of the references screened and was resolved by discussion.

Data extraction and management

Data were extracted by one review author (GG) and checked by another review author (HM). Data on the following was extracted from each article: author; country of study; study design; case definition of stress-related disorder; sources and number of participants; exposure definition; exposure assessment; exposure categories; risk estimate; and adjustment for confounders.

Methodological quality assessment

For the assessment of the risk of bias within studies, the quality criteria from the systematic review by Nieuwenhuijsen et al. [10] were used for all study designs, with nine items covering

For peer review only - http://bmjopen.bmj.com/site/about/guidelines.xhtml

the selection of participants, measurement of variables and control for confounding. The quality of the studies was independently assessed and agreed by two review authors (GG, HM). In total, nine items across five categories were evaluated for quality assessment: (1) the main features of the study population were stated e.g. sex, age and work context; (2) the participation rate at baseline was at least 50%; (3) the response at follow-up was at least 70% or the non-response was not selective; (4) the data on psychosocial work factors was collected using standardised instruments; (5) the data on outcome were collected at least at three different time points; (6) the statistical model used was appropriate for the outcome studied; (7) the measures of association with the statistical model were presented; (8) there were study controls for confounding with rationale; (9) the number of cases was at least 10 times the number of independent variables in the analysis. The criteria for each item were scored with 'positive', 'negative' or 'not clear'. A higher quality of a study was defined as ≥5 items scored as 'positive' out of the nine quality criteria.

Data synthesis

A descriptive analysis of all studies was performed, summarised, classified in categories of psychosocial risk factors, and assessed for methodological quality. Risk estimates and the corresponding 95% confidence intervals (CI) of the association between work-related factors and SRD were extracted and summarised. After assessing probable heterogeneity of risk factor and outcome assessment, all authors discussed and decided on the risk estimates to be included in the meta-analysis or should be analysed separately. We used the rule at least two studies when no statistical heterogeneity ($I^2 < 50\%$) and no heterogeneity in measures of risk factors and stress related disorders (as discussed among the authors) was found.

Meta-analyses and quality of evidence

For peer review only - http://bmjopen.bmj.com/site/about/guidelines.xhtml

The selection of the work-related risk factors in the meta-analyses was based on: (1) sufficient contrast between reported exposure categories, i.e. low versus high exposure; (2) effect estimates controlled for other non-work-related factors, as reported in the primary studies; 3) homogeneity in definition or measurement of risk factors.

The meta-analysis was performed in line with the procedure described in Watenabe et al. [13]: by using a log-transformed OR and its SE in Review Manager (Cochrane Review Manager version 5.3). For the main analysis, the main ORs and the SEs from selected studies were subjected to a random-effects model meta-analysis to estimate a pooled OR and its 95% CI.

The quality of evidence - also taken into account the risk of bias across studies - was assessed using the GRADE (Grades of Recommendations, Assessment, Development and Evaluation) framework for prognostic studies developed by Huguet et al. [14]. Following Huguet et al. [14], our starting point for the quality of the evidence was 'moderate' for prospective cohort studies that aimed to identify associations between potential prognostic factors (in our review risk factors) and the outcome. The evidence could decrease on the basis of five factors: study limitations, inconsistency, indirectness, imprecision and publication bias. Moreover, two factors: (1) study findings with moderate or large effect sizes (i.e. lower limit of 95% CI OR≥2.0) or (2) an exposure-response gradient, could lead to an upgrade of the quality of evidence. Four levels of quality were used: high, moderate, low and very low.

Results

Selected studies

A PRISMA flow diagram of the study selection process is shown in Figure 1. After excluding duplicates, 10,801 references were retrieved from the databases and the systematic review by

For peer review only - http://bmjopen.bmj.com/site/about/guidelines.xhtml

Nieuwenhuijsen et al. [10], and assessed based on title and abstract. The full texts of 139 potentially eligible articles were then examined, of which 17 studies from 16 articles met the inclusion criteria.

Figure 1 about here

Psychosocial risk factors and SRD

In total, seventeen longitudinal studies in sixteen articles[1-3, 15-27] described the association between exposure to psychosocial risk factors and the occurrence of assessed mental SRD. The characteristics of the outcome definition (SRD), exposure definition and reported associations are presented in table 1.

For peer review only - http://bmjopen.bmj.com/site/about/guidelines.xhtml

Table 1. Associations between psychosocial risk factors at work and the occurrence of mental stress related disorders (17 studies)				
**Author, Country Population**	**Outcome**	**Exposure**		
	**Definition & assessment**	**Definition & assessment**	**OR**	**(95%CI)**
Mino 1999 [15]<br>Japan, machine production, N=310	General Health Questionnaire (GHQ-30)<br>GHQ-30 ≥ 8	Job demands: one item<br>*(always/sometimes present vs absent)*<br>Supervisor support: one item<br>*(absent vs always/sometimes present)*	♂ RR 1.39<br>♀ RR 1.14<br>♂ RR 1.10<br>♀ RR 2.21	0.92-2.10<br>0.81-1.59<br>0.69-1.74<br>1.25-3.89
Stansfeld 1999 [16]<br>England, civil servants, N=10,308	General Health Questionnaire (GHQ-30)<br>GHQ-30 > 4	Job demands: adapted JCQ<br>*(highest tertile vs lowest)*<br>Decision authority: adapted JCQ<br>(*lowest vs highest tertile*)<br>Co-worker support: adapted JCQ<br>(*lowest vs highest tertile*)<br>Supervisor support: adapted JCQ<br>(*lowest vs highest tertile*)<br>Effort-reward imbalance: indicator of high effort and low rewards<br>*(high efforts/low rewards vs no high efforts/low rewards)*<br>Skill discretion: adapted JCQ<br>(*lowest vs highest tertile*)	♂ 1.33<br>♀ 1.24<br>♂ 1.29<br>♀ 1.37<br>♂ 1.29<br>♀ 1.12<br>♂ 1.31<br>♀ 1.11<br>♂ 2.57<br>♀ 1.67<br>♂ 1.11<br>♀ 1.09	1.1-1.6<br>1.0-1.6<br>1.1-1.5<br>1.1-1.8<br>1.1-1.5<br>0.9-1.4<br>1.1-1.5<br>0.9-1.3<br>1.8-3.6<br>1.0-2.9<br>0.9-1.3<br>0.8-1.4
Bültmann 2002 [1]<br>Netherlands, 45 companies, N=8,833	General Health Questionnaire<br>GHQ-12 ≥ 4	Job demands Questionnaire (JCQ)<br>Job demands: JCQ<br>(*highest vs lowest tertile*)<br>Decision latitude: JCQ<br>(*lowest vs highest tertile*)<br>Co-worker support: JCQ<br>*(low vs high)*<br>Supervisor support: JCQ<br>*(low vs high)*<br>Emotional demands: Dutch Questionnaires; Perception and Judgement of Work (QPJW), Work and Health, self-formulated *(high (2-5) vs no (0,1)*<br>Job insecurity: QPJW, one-item<br>(*yes vs no*)	 ♂ 1.51<br>♀ 1.44<br>♂ 1.14<br>♀ 0.88<br>♂ 1.25<br>♀ 1.31<br>♂ 1.25<br>♀ 1.12<br>♂ 1.73<br>♀ 1.39<br>♂ 1.63<br>♀ 0.94	 1.23-1.85<br>1.03-2.01<br>0.9-1.43<br>0.62-1.24<br>1.04-1.49<br>0.97-1.78<br>1.05-1.49<br>0.85-1.47<br>1.40-2.14<br>1.01-1.91<br>1.18-2.27<br>0.56-1.59
Bonde 2005 [17]	Setterlind Stress Profile Inventory (SPI)	Skill discretion: Repetitive work	1.3	0.6-2.2

For peer review only - http://bmjopen.bmj.com/site/about/guidelines.xhtml

Denmark, various workplaces, N=2,846	SSPI ≥ 4	*(yes vs no)*		
Godin 2005 [18] Belgium, privat and public sector, N=1,521	Short Fatigue Inventory (SHI)<br>SHI: upper quartile	Effort-Reward Imbalance Questionnaire<br>*(highest quartile vs rest)*	♂ 3.4<br>♀ 2.0	1.7-6.7<br>0.9-4.1
Kivimaki 2007 [19] Finland, government N=21,271	General Health Questionnaire<br>GHQ-12 ≥ 4	Effort-Reward Imbalance Questionnaire<br>*(highest vs lowest quartile)*<br>Procedural & organisational injustice: Organizational Justice Scale<br>*(highest vs lowest quartile)*<br>Relational injustice: Organizational Justice Scale<br>*(highest vs lowest quartile)*	2.04<br><br>1.81<br><br>1.50	1.80-2.32<br><br>1.60-2.06<br><br>1.32-1.70
Kivimaki 2007 [19] Finland, hospital N=10,736	General Health Questionnaire<br>GHQ-12 ≥ 4	Effort-Reward Imbalance Questionnaire<br>*(highest vs lowest quartile)*<br>Procedural injustice: Organizational Justice Questionnaire (OJQ)<br>*(highest vs lowest quartile)*<br>Relational injustice: OJQ *(highest vs lowest quartile)*	1.59<br><br>1.67<br><br>1.56	1.24-2.05<br><br>1.29-2.15<br><br>1.21-2.02
M- Hanson 2008 [2] Sweden, various workers, N=3,004	Maslach Burnout Inventory-general Survey MBI-GS ≥ 75th percentile	Job demands: Swedish Work Environment Survey (SWES)<br>*(≥2 positive vs < 2 out of four items)*<br>Decision authority: SWES<br>*(≥2 positive vs < 2 out of four items)*<br>Co-worker support: SWES; one item<br>*(4 point Likert scale, dichotomized: low (1,2) vs high (3.4))*<br>Supervisor support: SWES; one item<br>*(4 point Likert scale dichotomized: low (1,2) vs high (3.4))*	♂ 2.09<br>♀ 1.79<br>♂ 1.36<br>♀ 1.41<br>♂ 1.45<br>♀ 1.92<br>♂ 1.65<br>♀ 1.22	1.52-2.88<br>1.36-2.35<br>0.98-1.88<br>1.07-1.86<br>0.97-2.17<br>1.25-2.93<br>1.19-2.31<br>0.91-1.65
Devereux 2011 [20] England, 11 industrial types, N=1,463	General Health Questionnaire (GHQ-12)<br>GHQ-12 ≥ 3	Job demands questionnaire: 4 items<br>(*highest vs lowest tertile*)<br>Decision latitude: 15 items<br>(*lowest vs highest tertile*)<br>Co-worker and supervisor support: 7 items<br>(*lowest vs highest tertile*)	RR 1.62<br><br>RR 1.11<br><br>RR 1.47	1.26-2.09<br><br>0.86-1.42<br><br>1.18-1.84
Sundin 2011 [21] Sweden, nurses, N=555	Maslach Burnout Inventory (MBI) Swedish version, Emotional Exhaustion (EE)	Job demands: Swedish Work Environmental Survey (SWES), one item, 5 point Likert scale *(at least once a week or more vs ≤1 day out of 10)*<br>Co-worker support: SWES; one item, 4 point Likert scale	4.33<br><br>2.21	1.98-9.45<br><br>0.88-5.56

For peer review only - http://bmjopen.bmj.com/site/about/guidelines.xhtml

	EE ≥ 27	*(less (1,2,3) vs always receiving support (4))*<br>Supervisor support: SWES; one item, 4 point Likert scale<br>*(less (1,2,3) vs always receiving support (4))*	 2.17	 0.65-7.26
Inoue 2013 [3]<br>Japan, manufacturing, N=569	Kessler Psychological Distress Scale (K6)<br>≥5 K6 scale	Procedural injustice: Organizational Justice Questionnaire (OJQ): 7 items, 5 point scale; Permanent (P) non-permanent worker (NP) *(highest vs lowest tertile)*<br>Relational injustice: OJQ interactional justice 6-items, 5 point scale *(highest vs lowest tertile)*	♂ 1.37 (P)<br>♀ 5.25 (P)<br>♀ 2.84 (NP)<br>♂ 2.39 (P)<br>♀ 2.35 (P)<br>♀ 1.61 (NP)	0.43-4.34<br>0.82-33.6<br>1.19-6.75<br>0.61-9.39<br>0.51-10.9<br>0.78-3.30
Laine 2014 [22]<br>Finland, public sector, N=3,298	General Health Questionnaire GHQ-12<br>≥3 GHQ-12	Procedural injustice: 4 items Moorman's inventory *(highest vs lowest quartile)*<br>Relational injustice: 4 items Moorman's inventory *(highest vs lowest quartile)*<br>Bullying: item about mental violence or workplace bullying *(yes vs no)*	1.65<br><br>1.29<br><br>1.57	1.21-2.24<br><br>0.97-1.72<br><br>0.97-2.53
Taniguchi 2015 [23]<br>Japan, elderly facilities, N=543	Brief Job Stress Questionnaire (BJSQ)<br>BJSQ > 13 (♂) or >12 (♀)	Bullying: Japanese Negative Acts Questionnaire (NAQ), person-related bullying (PR; 6 items), work-related bullying (WR; 3 items), sexual harassment (SH; 3 items), five point scale. *(≥1 item positive versus none)*	3.46 (PR)<br>2.85 (WR)<br>1.73 (SH)	1.49-8.05<br>0.61-13.26<br>0.98-3.08
Andersen 2017 [24]<br>Denmark, prison personnel, N=1741	Copenhagen Burnout Inventory (COPSOQ-CBI)<br>COPSOQ-CBI highest quartile	Copenhagen Psychosocial Questionnaire (COPSOQ), 5-point Likert-scale (*most exposed quartile vs least exposed three quartiles)*<br>Job demands: 4 items<br>Co-worker and supervisor support: 6 items<br>Emotional demands: 4 items<br>Effort-reward imbalance (low recognition): 3 items	 <br>1.61<br>1.31<br>1.46<br>1.33	 <br>1.21-2.16<br>0.98-1.76<br>1.06-2.01<br>0.98-1.80
Oshio 2017 [25]<br>Japan, 12 industrial types, N=7419	Kessler Psychological Distress Scale (K6)<br>K6 ≥ 5	Job demands: 5 items Japanese Job Content Questionnaire (JCQ), 4-point scale *(median-split method classifying high vs low)*<br>Procedural injustice: Japanese version of Organisational Justice Questionnaire (OJQ), 7 items, 5-point Likert scale<br>Relational injustice: Japanese version of Organisational Justice Questionnaire (OJQ), 6 items, 5-point Likert scale<br>Effort reward imbalance: Japanese version Effort-Reward Imbalance Questionnaire, effort (3 items), reward (7 items), 4-point scale	♂ 1.62<br>♀ 1.71<br>♂ 1.68<br>♀ 1.80<br>♂ 1.58<br>♀ 1.71<br>♂ 2.05<br>♀ 1.84	1.45-1.81<br>1.38-2.13<br>1.51-1.87<br>1.45-2.23<br>1.42-1.77<br>1.37-2.13<br>1.84-2.29<br>1.48-2.28
Kind 2018 [26]	Burnout screening scales (BOSS)	Workplace aggression: exposure to verbal and physical threats		

For peer review only - http://bmjopen.bmj.com/site/about/guidelines.xhtml

Switzerland, youth welfare, N=121	BOSS T-score ≥60	*Verbal aggression vs no aggression*	HR 1.67	1.09-2.58
P-Thingvad 2019 [27] Denmark, social educators, N=1823	Copenhagen Burnout Inventory (CBI) CBI ≥ 75th percentile	Workplace violence: Scandinavian checklist, 4 items, 4-point Likert-scale *(high exposed (≥1 incidents per month) vs non- exposed)*	1.4	0.9-2.3

For peer review only

For peer review only - http://bmjopen.bmj.com/site/about/guidelines.xhtml

Table 2. Methodological quality scores of 9 items for studies regarding risk factors*										
**Author**	**1**	**2**	**3**	**4**	**5**	**6**	**7**	**8**	**9**	**Summary score**
Mino 1999	-	+	-	-	+	+	+	-[a]	+	5
Stansfeld 1999	-	+	+	-	+	+	+	-[b]	+	6
Bϋltmann 2002	+	-	+	+	-	+	+	-[c]	+	6
Bonde 2005	+	+	+	-	+	+	+	+[d]	+	8
Godin 2005	+	-	+	+	-	+	+	-[e]	+	6
Kivimaki 10-town study 2007	+	+	+	-	-	+	+	-[f]	+	6
Kivimaki hospital study 2007	+	+	+	+	-	+	+	-[f]	+	7
Magnusson Hanson 2008	+	+	-	-	-	+	+	-[g]	+	5
Devereux 2011	+	?	-	+	-	+	+	-[h]	+	5
Sundin 2011	+	+	-	+	-	+	+	+[i]	+	7
Inoue 2013	+	+	+	+	-	+	+	+[j]	+	8
Laine 2014	-	+	+	+	-	+	+	+[k]	+	7
Taniguchi 2015	+	+	-	+	-	+	+	+[l]	+	7
Andersen 2017	+	+	-	+	-	+	+	+[m]	+	7
Oshio 2017	+	+	+	+	-	+	+	+[n]	+	8
Kind 2018	+	-	+	+	+	+	+	+[0]	+	8
Pihl-Thingvad 2019	+	+	+	+	-	+	+	+[p]	+	8
**Total item score**	14	13	11	12	4	17	17	9	17	

* Criteria and scoring options for quality score for these 9 items are reported in the methods section
[a]Adjusted for age, sex, family life satisfaction, perceived physical health
[b]Adjusted for age, sex, employment grade and baseline GHQ score
[c]Adjusted for age, sex, education, living alone, employment status, presence of disease, baseline fatigue score
[d]Adjusted for age, sex, body mass index, leisure time activity, pain threshold, marital status, psychiatric disorder
[e]Adjusted for age, sex, education, threat from global economy, job dissatisfaction, workplace instability
[f]Adjusted for age, sex, occupational status
[g]Adjusted for age, sex, marital status, country of birth, social class, physical exhaustion
[h]Adjusted for age, sex, shift work
[i]Adjusted for age, sex, marital status, years of (current) employment (only for job demands)
[j]Adjusted for age, sex, education, marital status, chronic physical diseases, occupation, life events, neuroticism
[k]Adjusted for age, sex, socioeconomic position, marital status, employment, health behaviour, limiting longstanding illness, physical work
[l]Adjusted for age, sex, job carrier, occupation, marital status, employment, work shift, smoking status
[m]Adjusted for age, sex, marital status, employment years, occupational characteristics and exposures
[n]Adjusted for age, sex, education, occupation, hours worked per week, household income, family member to share living expenses, firm codes
[o]Adjusted for age, sex, work experience, employment years, private stressors
[p]Adjusted for age, sex, somatic and mental health at baseline, lifestyle factors, work-related factors

For peer review only

Table 3. Quality of the evidence for the relationship between risk factors and stress related disorders according to the GRADE framework

	**Number of participants**	**Number of studies / cohorts**	**Study phase***	**Study limitations**<br><br>**Study quality majority of studies < 11/16: ↓**	**Inconsistency**<br><br>**$I^2 \geq 50\%$: ↓**	**Indirectness**<br><br>**Yes: ↓**	**Imprecision**<br><br>**Confidence interval (CI) effect size (<1 and >2) ~~range >2)~~ Yes: ↓**	**Publication Bias**<br><br>**Yes: ↓**	**Effect size OR (95% CI)**<br><br>**Lower Limit OR ≥ 2.0 : ↑**	**Exposure-response gradient (dose-effect)**<br><br>**Majority of studies: ↑**	**Overall Quality of Evidence**
Effort-reward imbalance	76,760	6/6	1	no	48%	no	no	no	1.91 (1.70-2.15)	2/6	Moderate
Low procedural justice	64,676	5/5	1	no	0%	no	no	no	1.74 (1.62-1.86)	2/5	Moderate
Low relational justice	64,676	5/5	1	no	0%	no	no	no	1.55 (1.44-1.67)	2/5	Moderate
High job demands	41,397	8/8	1	no	49%	no	no	no	1.56 (1.41-1.72)	0/8	Moderate
Low co-worker support	22,920	4/4	1	no	10%	no	no	no	1.29 (1.17-1.43)	0/4	Moderate
Low supervisor support	23,382	5/5	1	no	0%	no	no	no	1.27 (1.16-1.38)	0/5	Moderate
Low co-worker and supervisor support	7,262	2/2	1	no	0%	no	no	no	1.41 (1.18-1.69)	0/2	Moderate
High emotional demands	13,641	2/2	1	no	0%	no	no	undetected	1.58 (1.35-1.84)	0/2	Moderate
Low decision authority	13,312	2/2	1	no	0%	no	no	undetected	1.34 (1.20-1.49)	0/2	Moderate
Job insecurity	8,833	1/1	1	no	-	no	yes: ↓	undetected	Men 1.63 (1.18-2.27) Women 0.94 (0.56-1.59)	0/1	Low
Decision latitude	11,287	2/2	1	no	0%	no	no	undetected	1.07 (0.92-1.25)	0/2	Moderate
Low skill discretion	3,123	2/2	1	no	0%	no	no	undetected	1.11 (0.94-1.32)	0/2	Moderate
Bullying & violence	9,694	4/4	1	no	Heterogeneity in definitions	no	yes: ↓	no	1.42 – 3.64 (0.42-15.92)	0/4	Low

* All included studies are phase 1 explanatory prospective cohort studies with 'moderate' as starting point for the quality of the evidence

For peer review only

For peer review only - http://bmjopen.bmj.com/site/about/guidelines.xhtml

Methodological quality

The methodological quality of the studies of risk factors varied from scoring 5 out of 9 items to 8 out of 9 items (see table 2). The most frequently missing quality items were: response at follow-up less 70%, data on outcome were collected at least at three different time points, and study controls for confounding with rationale.

Meta-analyses and assessment of evidence

In total, twelve psychosocial risk factors from 14 studies [1-3, 15-22, 24,25] from a population of 73,874 workers from Belgium, Denmark, England, Finland, Japan, Netherlands and Sweden were used in the meta-analysis. Reported distress prevalence varied from 4.9% [17] to 45.8% [3]. The follow-up time varied from one to five years. Table 3 summarises the assessment of evidence concerning the psychosocial risk factors for stress-related disorders.

Effort-reward balance

Six cohort studies [16,18,19,24,25] demonstrate that there is moderate-quality evidence that effort-reward imbalance increases the incidence of SRD, with a pooled OR of 1.91 (95% CI 1.70-2.15) (see table 3 and figure 2). No statistically significant difference between subgroups of men and women were found [16,18,25].

Procedural and relational justice

Five cohort studies [3,19,22,25] demonstrate that there is moderate-quality evidence that low procedural justice with a pooled OR of 1.73 (95% CI 1.61-1.86) and low relational justice with a pooled OR of 1.55 (95%CI: 1.44-1.67) increase the incidence of SRD (see table 3 and figure 3). No statistically significant difference between subgroups of men and women were found [3,25].

For peer review only - http://bmjopen.bmj.com/site/about/guidelines.xhtml

Job demands

Eight cohort studies [1,2,15,16,20,21,24,25] demonstrate that there is moderate-quality evidence that high job demands increases the incidence of SRD, with a pooled OR of 1.55 (95% CI 1.41-1.71) (see table 3 and figure 4). No statistically significant difference between subgroups of men and women were found [1,2,15,16,25].

Co-worker and supervisor support

In total, five cohort studies demonstrate that there is moderate-quality evidence that low co-workers support [1,2,16,21] with a pooled OR 1.29 (95%CI: 1.17-1.43) and low supervisor support [1,2,15,16,21] with a pooled OR 1.27 (95%CI: 1.14-1.40) increase the incidence of SRD. No statistically significant difference between subgroups of men and women were found [1,215,16]. The combination of low co-worker and supervisor support [20,24] resulted in a pooled OR 1.41 (95%CI 1.18-1.69). (see table 3).

Emotional demands

Two cohort studies [1,24] demonstrate that there is moderate-quality evidence that high emotional demands increase the incidence of SRD, with a pooled OR of 1.58 (95% CI 1.35-1.84) (see table 3). No statistically significant difference between men and women were found in the study of Bültmann et al. 2002 [1].

Decision authority

Two cohort studies [2,16] demonstrate that there is moderate-quality evidence that low decision autonomy increases the incidence of SRD with a pooled OR of 1.34 (95% CI 1.20-

For peer review only - http://bmjopen.bmj.com/site/about/guidelines.xhtml

1.49) (see table 3). No statistically significant difference between subgroups of men and women were found.

Job security, decision latitude, skill discretion and bullying

One cohort study [1] demonstrates that there is low-quality evidence that job insecurity increases the incidence of SRD, with a pooled OR of 1.63 (95% CI 1.18-2.27) for male and no increase of the incidence for female with a pooled OR of 0.94 (95% CI 0.56-1.59). Two cohort studies [1,20] demonstrate that there is moderate-quality evidence for no increased incidence of SRD due to low decision latitude, with an OR of 1.07 (95% CI 0.92-1.25). Two cohort studies [16, 17] demonstrate that there is moderate-quality evidence for no increased incidence of SRD due to low skill discretion, with an OR of 1.11 (95% CI 0.94-1.32). Four cohort studies [22,23,26,27] demonstrate very low-quality evidence that there is inconsistent evidence for increased incidence of SRD with different types of bullying and workplace violence with ORs varying from 1.42 (95%CI 0.42-4.79) to 3.64 (95%CI 0.83-15.92). (see table 3). Laine et al. [22] studied workplace bullying or mental violence as the isolation of a member of the organisation, the underestimation of work performance, being threatened, being talked about behind one's back or other forms of pressure. Taniguchi et al. [23] studied person related bullying, workplace bullying and sexual harassment. Kind et al. [26] studied verbal and physical client aggression and Phil-Thingvad et al. [27] studied physical violence.

Figures 2 - 3 – 4 about here

For peer review only - http://bmjopen.bmj.com/site/about/guidelines.xhtml

DISCUSSION

Main findings

This systematic review, including a meta-analysis of prospective cohort studies revealed moderate-quality evidence that effort-reward imbalance, low procedural and relational justice, high job demands, low co-worker and supervisor support, high emotional demands, and low decision authority increase the incidence of stress-related disorders, varying from 20% to 90%. Low-quality evidence was found for an association between job insecurity and stress-related disorder among men. Moderate to low-quality evidence suggesting no associations were found for decision latitude, skill discretion and bullying.

Psychosocial risk factors and SRD

All prospective cohort studies were controlled for the personal factors of age and sex, and non-work-related factors such as education and marital status. Six studies also controlled for other work-related psychosocial risk factors in various models [3,19,20,22,27]. Regarding these studies, we decided not to take into account the fully adjusted model in the meta-analyses in order to avoid overcorrection, and strengthen transparency and comparability between studies. However, these models yielded small and non-statistical significant differences between the risk estimates.

All studies defined SRD based on self-report with validated and standardised questionnaires, i.e. (shortened) GHQ [1,15,16,19,20,22], Kessler 6 [3,25], MBI [2,21], COPSOQ-CBI [24,27], Setterlind Stress Inventory Profile [17], fatigue inventory [18], Brief job Stress Questionnaire (BJSQ) [27], burnout screening scale (BOSS) [26].

The pooled ORs of the meta-analyses of psychosocial risk factors were equal or slightly raised compared with the prior results of Nieuwenhuijsen et al. [10] and in line with the findings of a

For peer review only - http://bmjopen.bmj.com/site/about/guidelines.xhtml

recent meta-review of work-related risk factors for common mental-health problems including depression and anxiety [11].

Methodological considerations

The exposure categories for the risk factors and the case definitions of SRD varied in the included studies. We have explicitly described all definitions and assessments of self-reported exposures and outcomes (see table 1). In general, psychological distress can stem from private life as well as from work. Therefore we only included high quality prospective studies aimed at identifying work-related risk factors, also controlling for other factors outside the work context. Although there is debate about the definition of burnout [5,7, 28], we regarded distress and burnout as outcomes of the same continuum. We found that similar work-related psychosocial risk factors were associated with distress and the one-dimensional measures of burn-out ('exhaustion'). Should more studies using the multiple dimension definition of burnout become available, then subgroup analyses of these studies can be performed to assess whether the work-related risk factors are similar in this diagnostic subgroup.

We used the risk estimates - mostly ORs - as reported in the original prospective cohort studies (see also table 1). Meta-analyses were performed to estimate pooled associations, although the variety in measurement instruments and dichotomization rules introduced heterogeneity in the outcome measures (case definitions) and exposure categories of psychosocial risk factors. However, the exposure categories for the psychosocial risk factors included in the meta-analyses were already established in theoretical models of effort-reward imbalance [29], job demands-control [30] and organisational justice [31]. Furthermore, the case definitions of SRD used validated questionnaires and the methodological quality of the included studies was high and accompanied by a low to moderate statistical heterogeneity across the pooled studies (I^2 varying from 0% to 49%) which justify the meta-analyses. With the low number of studies for

For peer review only - http://bmjopen.bmj.com/site/about/guidelines.xhtml

each risk factor we feel that more subgroup analyses – besides the meta-analyses for separate risk factors and sexes – are not informative. Reporting bias is a concern because both psychosocial factors and SRD are self-reported in the included studies; it is conceivable that certain psychological states at baseline are associated both with the over-reporting of adverse working conditions at baseline and with the risk of onset of stress-disorders at follow-up [32]; however, in prospective studies this risk is less pronounced [33].

The GRADE framework made it possible to assess to the quality of evidence in reviews of prognostic factor research. However, the starting level for grading of the evidence for prospective etiologic studies is subject to scientific debate. For intervention studies, the grading of observational studies start with low evidence. For prospective observational studies, which can answer prognostic, or as in our systematic review, etiological research questions, Huguet et al. [14] suggest that higher levels of evidence are derived from cohort studies that seeks to confirm independent associations between the prognostic factor and the outcome (high level of evidence) or – as in our systematic review – aimed to identify associations between potential prognostic factors and the outcome (moderate level of evidence). Also recent GRADE guidelines state that best evidence regarding prognostic factors usually originates from observational studies (e.g. cohort studies or registries), while randomized control trials could include restrictions that exclude subjects relevant for the assessment of prognostic factors [34]. Based on Huguet et al. [14] we classified the risk of publication bias as 'no bias' when the value of the risk factor in predicting the outcome has been repetitively investigated in explanatory studies. We provided no funnel plots because the number of cohort studies for each psychosocial risk factor was substantially lower than 10. Visually checking the funnel plots, however, suggested no serious publication bias or could not assessed [undetected publication bias].

For peer review only - http://bmjopen.bmj.com/site/about/guidelines.xhtml

For the risk factor, bullying and violence, it was decided not to perform a meta-analysis because of the different concepts of the psychosocial risk factor, i.e. person-related bullying, work-related bullying, sexual harassment, workplace bullying, threatening or violence.

Eventually, further research in harmonizing the assessment of SRD in cohort studies [7] and the clinical assessment with signs and symptoms of SRD [35], combined with more detailed or objective task-based exposure assessment [36] will contribute to a better insight into the work-related psychosocial risk factors and their mediating or moderating factors.

Prevention

To manage and prevent stress disorders it is necessary to identify which psychosocial risk factors at work contribute to the onset of SRD. West et al. [37] describe the importance of organisational strategies in their review on interventions to prevent and reduce SRD among physicians. Kalani et al. [38] report in their meta-review that various studies of the effectiveness of individually and organisationally directed interventions have led to different results on reducing physician burnout. Ultimately, to develop individually and organisationally directed interventions it is necessary that the relevant psychosocial risk factors in organisations are known [37,38].

Recently, Fan et al. [39] has suggested that some psychosocial factors such as job control, job security and social support are also associated with a greater likelihood of workers' experiencing positive mental well-being in terms of satisfaction and purpose in life, personal growth, social contribution and integration. This study implicates the double value of workplace policies and practices that improve psychosocial working conditions, reduce work-related SRD and improve mental well-being in general, e.g. by giving workers greater job control or social support [39].

For peer review only - http://bmjopen.bmj.com/site/about/guidelines.xhtml

In conclusion, several psychosocial work-related risk factors for SRD were established, confirming the multifactorial aetiology of SRD. Effort-reward imbalance, low organisational justice and high job demands were associated with the largest increased risk of SRD, varying from 60 to 90%. Awareness of these risk factors could be the starting point for the selection of preventive interventions to reduce work-related SRD.

Acknowledgement

We are very grateful to Joost Daams for his contribution to the literature search and Nicolaas Kylstra and Kim van der Molen for their help with screening activities.

Competing interests

The authors declare that they have no competing interests.

Contributors

HM and GG initiated the study, performed data extraction and statistical analyses, and drafted the manuscript. All authors performed the study selection and interpretation of the data. All authors made substantial contributions to the conception of the study and manuscript. HM and GG equally contributed.

Funding

The work was sponsored by the Ministry of Social Affairs and Employment, the Netherlands. Grant number: 5100-24108

Competing interests None declared.

For peer review only

For peer review only - http://bmjopen.bmj.com/site/about/guidelines.xhtml

Patient consent for publication Not required.

Provenance and peer review Not commissioned; externally peer reviewed.

Data sharing statement No additional data available.

For peer review only

For peer review only - http://bmjopen.bmj.com/site/about/guidelines.xhtml

References

1. Bultmann U, Kant IJ, van den Brandt PA et al. Psychosocial work characteristics as risk factors for the onset of fatigue and psychological distress: prospective results from the Maastricht Cohort Study. *Psychol Med* 2002;32:333-345.
2. Magnusson Hanson LL, Theorell T, Oxenstierna G, Hyde M, Westerlund G. Demand, control and social climate as predictors of emotional exhaustion symptoms in working Swedish men and women. *Scand J Public Health* 2008; 36: 737-743.
3. Inoue A, Kawakami N, Tsuno K, Tomioka K, Nakanishi M. Organizational justice and psychological distress among permanent and non-permanent employees in Japan: a prospective cohort study. *Int J Behav Med* 2013; 20: 265-276.
4. van der Molen HF, de Vries S, Sluiter JK. Occupational diseases among workers in lower and higher socioeconomic positions. *Int J Environ Res Public Health* 2018;15(12):2849.
5. WHO. Burn-out an "occupational phenomenon": International Classification of DiseasesWhat are social determinants of health? 2019 https://www.who.int/mental_health/evidence/burn-out/en/?mc_cid=c8878361fa&mc_eid=8d4606394a (accessed 6 Aug 2019).
6. Lastovkova A, Cardner M, Rasmussen HM et al. Burnout syndrome as an occupational disease in the European Union: an exploratory study. *Ind Health* 2018;56(2):160-165.
7. Canu I, Bianchi R, Bubas M et al. Which factors contribute to the onset of burnout among workers? A systematic review. PROSPERO 2018 CRD42018105901 Available from: http://www.crd.york.ac.uk/PROSPERO/display_record.php?ID=CRD42018105901
8. Chirico F. Is burnout a syndrome or an occupational disease? Instructions for occupational physicians *Epidemiol Prev* 2017;41 (5-6):294-298.
9. Chirico F. The forgotten realm of the new and emerging psychosocial risk factors. *J Occup Health* 2017; 59(5):433-435.
10. Nieuwenhuijsen K, Bruinvels D, Frings-Dresen M. Psychosocial work environment and stress-related disorders, a systematic review. *Occup Med* (Lond) 2010;60:277-286
11. Harvey SB, Modini M, Joyce S et al. Can work make you mentally ill? A systematic meta-review of work-related risk factor for common mental health problems. *Occup Environ Med* 2017;74:301-310.
12. Milner A, Witt K, LaMontagne AD et al. Psychosocial job stressors and suicidality: a meta-analysis and systematic review. *Occup Environ Med* 2018;75:245-253.
13. Watanabe K, Imamura K, Kawakami N. Working hours and the onset of depressive disorder: a systematic review and meta-analysis. *Occup Environ Med* 2016;73(12):877-884.
14. Huguet A, Hayden J, Stinson J, McGrath P, Chambers C, Tougas M, Wozney L. Judging the quality of evidence in reviews of prognostic factor research: adapting the GRADE framework. *Syst Rev* 2013;2:71.
15. Mino Y, Shigemi J, Tsuda T et al. Perceived job stress and mental health in precision machine workers of Japan: a 2 year cohort study. *Occup Environ Med* 1999;56:41-45.
16. Stansfeld SA, Fuhrer R, Shipley MJ et al. Work characteristics predict psychiatric disorder: prospective results from the Whitehall II Study. *Occup Environ Med* 1999;56:302-307.
17. Bonde JP, Mikkelsen S, Andersen JH et al. Understanding work related musculoskeletal pain: does repetitive work cause stress symptoms? *Occup Environ Med* 2005;62:41-48.
18. Godin I, Kittel F, Coppieters Y et al. A prospective study of cumulative job stress in relation to mental health. *BMC Public Health* 2005;5:67.
19. Kivimaki M, Vahtera J, Elovainio M et al. Effort-reward imbalance, procedural injustice and relational injustice as psychosocial predictors of health: complementary or redundant models? *Occup Environ Med* 2007;64:659–665.

For peer review only - http://bmjopen.bmj.com/site/about/guidelines.xhtml

20. Devereux JJ, Rydstedt LW, Cropley M. Psychosocial work characteristics, need for recovery and musculoskeletal problems predict psychological distress in a sample of British Workers. *Ergonomics* 2011; 54(9),840-848.

21. Sundin L, Hochwälder J, Lisspers J. A longitudinal examination of generic and occupational specific job demands, and work-related social support associated with burnout among nurses in Sweden. *Work* 2011;38: 389-400.

22. Laine H, Saastamoinen P, Lahti J, Rahkonen O, Lahelma E. The associations between psychosocial working conditions and changes in common mental disorders: a follow-up study. *BMC Public Health* 2014;14-588.

23. Taniguchi T, Takaki J, Hirokawa K, Fujii Y, Harano K. Associations of workplace bullying and harassment with stress reactions: a two year follow-up study. *Ind Health* 2016,54,131-138.

24. Andersen D, Andersen L, Gadegaard C, Hogh A, Prieur A, Lund T. Burnout among Danish prison personnel: a Question of quantitative and emotional demands. *Scand J Public Health* 2017;45:824-830.

25. Oshio T, Inoue A, Tsutsumi A. Examining the mediating effect of work-to-family conflict on the associations between job stressors and employee psychological distress: a prospective cohort study. *BMJ Open* 2017;7:e015608.

26. Kind N, Eckert A, Steinlinc C, Fegert JM, Schmid M. Verbal and physical client aggression – A longitudinal analysis of professional caregivers' psychophysiological stress response and burnout. *Psychoneuroendocrinology* 2018;94:11-16.

27. Pihl‐Thingvad J, Elklit A, Brandt LPA, Andersen LL. Workplace violence and development of burnout symptoms: a prospective cohort study on 1823 social educators. *Int Arch Occ Env Hea* 2019;92:843-853.

28. Maslach C, Leiter MP. Understanding the burnout experience: recent research and its implications for psychiatry. *World Psychiatry* 2016;15(2):103-111.

29. Siegrist J, Siegrist K, Weber I. Sociological concepts in the etiology of chronic disease: the case of ischemic heart disease. *Soc Sci Med* 1986;22:247-253.

30. Karasek RA, Jr. Job demands, job decision latitude, and mental strain: implications for job design. *Admin Sci Q* 1979;24:285-308.

31. Moorman RH. Relationship between organizational justice and organizational citizenship behaviors: do fairness perceptions influence employee citizenship? *J Appl Psychol* 1991;76:845-855.

32. Rugulies R, Aust B, Madsen IEH. Effort–reward imbalance at work and risk of depressive disorders. A systematic review and meta-analysis of prospective cohort studies. *Scand J Work Environ Health* 2017;43(4):294-306.

33. Theorell T, Hammarström A, Aronsson G. A systematic review including meta-analysis of work environment and depressive symptoms. *BMC Public Health* 2015;15:738.

34. Foroutana F, Guyatt G, Zuka V et al. GRADE Guidelines 28: Use of GRADE for the assessment of evidence about prognostic factors: rating certainty in identification of groups of patients with different absolute risks. *Journal of Clinical Epidemiology* 2020;121:62-70.

35. Kakiashvili T, Leszek J, Rutkowski K The medical perspective on burnout. *Int J Occup Med Environ Health* 2013;26(3):401-12.

36. Göras C Olin K Unbeck M Pukk-Härenstam K. Tasks, multitasking and interruptions among the surgical team in an operating room: a prospective observational study. *BMJ Open* 2019;9(5):e026410.

37. West C, Dyrbye L, Erwin P, Shanafelt T. Interventions to prevent and reduce physician burnout: a systematic review and meta-analysis. *Lancet* 2016;388(10057):2272-2281.

38. Kalani S, Azadfallah P, Oreyzi H, Adibi P. Interventions for Physician Burnout: a systematic review of systematic reviews. *Int J Prev Med* 2018;9:81.

39. Fan JK, Mustard C, Smith PM. Psychosocial work conditions and mental health: examining differences across mental illness and well-being outcomes. *Annals of Work Exposures and Health* 2019;63 (5):546-559.

For peer review only

For peer review only - http://bmjopen.bmj.com/site/about/guidelines.xhtml

Figures, Tables & appendix

<u>Figures</u>

Figure 1 Prisma Flow Diagram

Figures 2 – 4 Forest Plots

<u>Tables</u>

Table 1. Associations between psychosocial risk factors at work and the occurrence of stress-related disorders (17 studies)

Table 2 Methodological quality scores of the studies including those concerning psychosocial risk factors

Table 3 Quality of the evidence of the relationship between psychosocial risk factors and stress-related disorders according to the GRADE framework

<u>Appendix</u>

Search of Medline, Embase and PsychInfo for studies between 2008 and 12 August 2019

For peer review only - http://bmjopen.bmj.com/site/about/guidelines.xhtml

Figure 1 PRISMA Flow Diagram

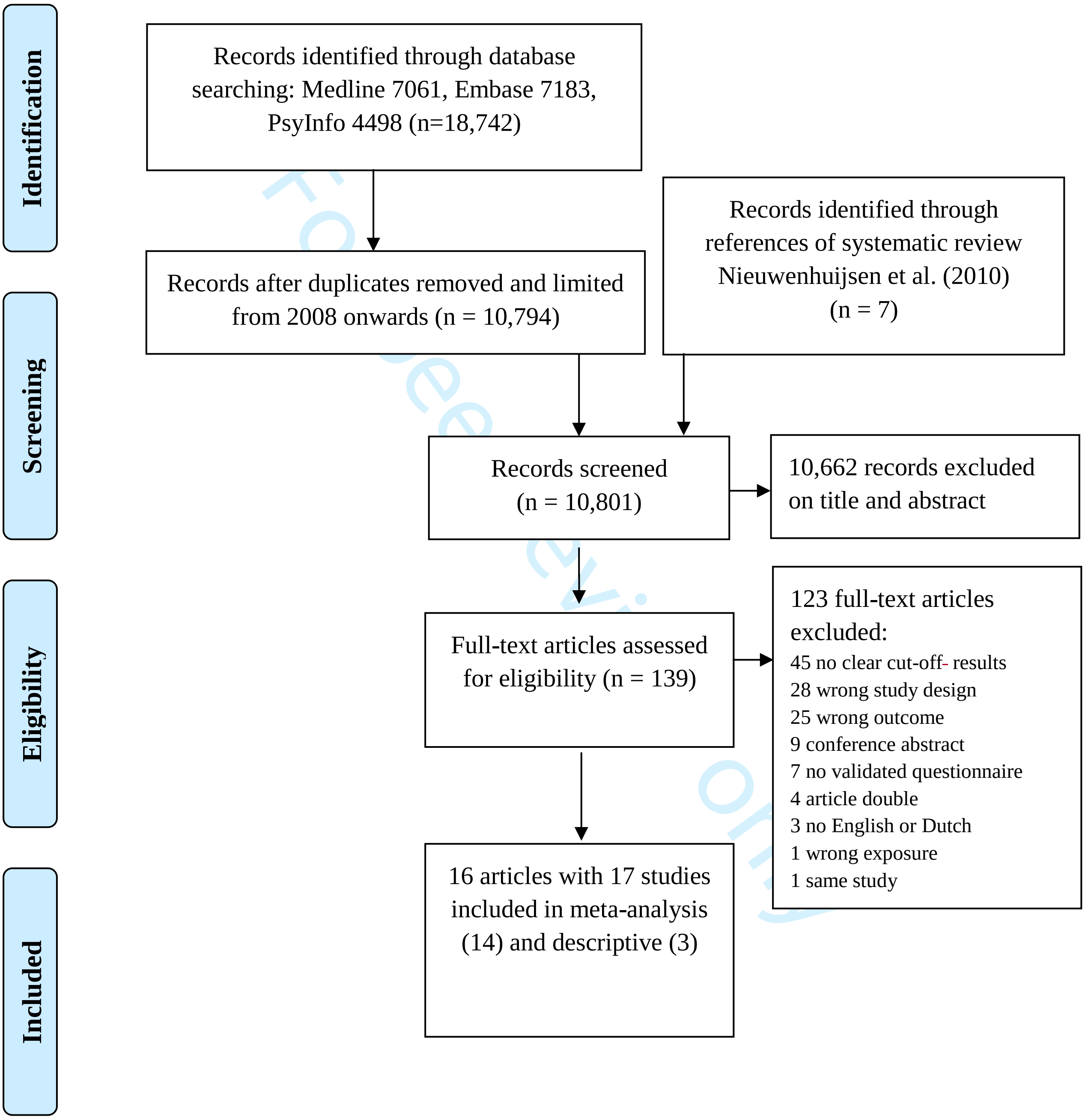

For peer review only - http://bmjopen.bmj.com/site/about/guidelines.xhtml

Study or Subgroup	log[Odds Ratio]	SE	Weight	Odds Ratio IV, Random, 95% CI
**7.1.1 men**				
Godin	1.2238	0.3537	2.7%	3.40 [1.70, 6.80]
Oshio 2017 man	0.7192	0.0558	23.3%	2.05 [1.84, 2.29]
Stansfeld	0.9439	0.1768	8.4%	2.57 [1.82, 3.63]
**Subtotal (95% CI)**			**34.3%**	**2.29 [1.82, 2.87]**
Heterogeneity: Tau² = 0.02; Chi² = 3.30, df = 2 (P = 0.19); I² = 39%				
Test for overall effect: Z = 7.16 (P < 0.00001)				
**7.1.2 women**				
Godin	0.6931	0.4074	2.1%	2.00 [0.90, 4.44]
Oshio 2017 vrouw	0.6081	0.1102	14.8%	1.84 [1.48, 2.28]
Stansfeld	0.5128	0.2716	4.3%	1.67 [0.98, 2.84]
**Subtotal (95% CI)**			**21.2%**	**1.82 [1.50, 2.21]**
Heterogeneity: Tau² = 0.00; Chi² = 0.16, df = 2 (P = 0.92); I² = 0%				
Test for overall effect: Z = 6.06 (P < 0.00001)				
**7.1.3 all**				
Andersen 2017	0.2838	0.1551	10.0%	1.33 [0.98, 1.80]
Kivimaki 2007a	0.7129	0.0647	21.8%	2.04 [1.80, 2.32]
Kivimaki 2007b	0.4637	0.1282	12.7%	1.59 [1.24, 2.04]
**Subtotal (95% CI)**			**44.5%**	**1.67 [1.29, 2.17]**
Heterogeneity: Tau² = 0.04; Chi² = 8.24, df = 2 (P = 0.02); I² = 76%				
Test for overall effect: Z = 3.87 (P = 0.0001)				
**Total (95% CI)**			**100.0%**	**1.91 [1.70, 2.15]**
Heterogeneity: Tau² = 0.01; Chi² = 15.50, df = 8 (P = 0.05); I² = 48%				
Test for overall effect: Z = 10.67 (P < 0.00001)				
Test for subgroup differences: Chi² = 3.64, df = 2 (P = 0.16), I² = 45.1%				

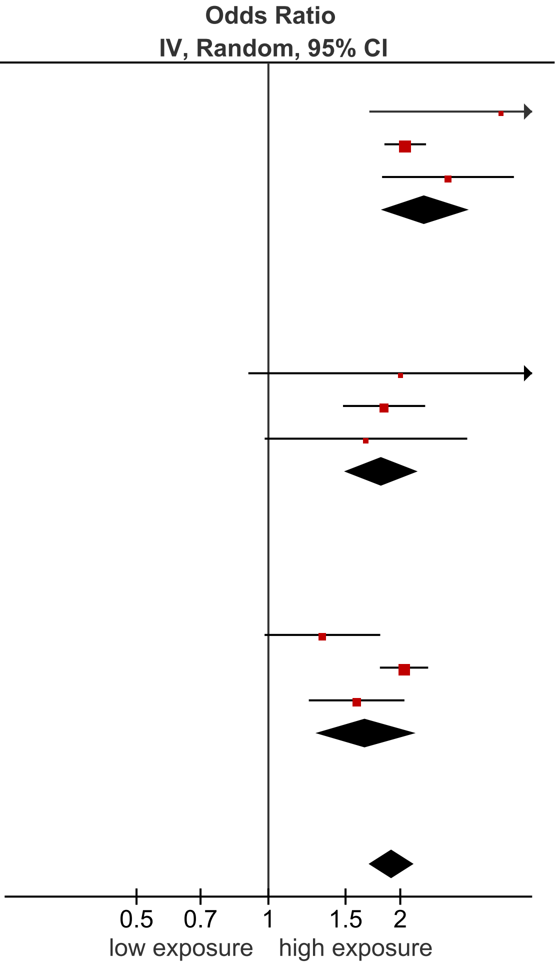

Study or Subgroup	log[Odds Ratio]	SE	Weight	Odds Ratio IV, Random, 95% CI
**5.1.1 All**				
Kivimaki 2007a	0.5933	0.0645	31.3%	1.81 [1.60, 2.05]
Kivimaki 2007b	0.5128	0.1303	7.7%	1.67 [1.29, 2.16]
Laine	0.5008	0.1582	5.2%	1.65 [1.21, 2.25]
Oshio 2017 man	0.5188	0.0544	44.0%	1.68 [1.51, 1.87]
Oshio 2017 vrouw	0.5878	0.1103	10.7%	1.80 [1.45, 2.23]
**Subtotal (95% CI)**			**98.8%**	**1.73 [1.61, 1.86]**
Heterogeneity: Tau² = 0.00; Chi² = 1.07, df = 4 (P = 0.90); I² = 0%				
Test for overall effect: Z = 15.11 (P < 0.00001)				
**5.1.2 P male, P female, NP female**				
Inoue	0.3148	0.5912	0.4%	1.37 [0.43, 4.36]
Inoue 2	1.6582	0.9473	0.1%	5.25 [0.82, 33.61]
Inoue 3	1.0438	0.4438	0.7%	2.84 [1.19, 6.78]
**Subtotal (95% CI)**			**1.2%**	**2.43 [1.27, 4.67]**
Heterogeneity: Tau² = 0.00; Chi² = 1.72, df = 2 (P = 0.42); I² = 0%				
Test for overall effect: Z = 2.67 (P = 0.007)				
**Total (95% CI)**			**100.0%**	**1.74 [1.62, 1.86]**
Heterogeneity: Tau² = 0.00; Chi² = 3.84, df = 7 (P = 0.80); I² = 0%				
Test for overall effect: Z = 15.31 (P < 0.00001)				
Test for subgroup differences: Chi² = 1.04, df = 1 (P = 0.31), I² = 3.6%				

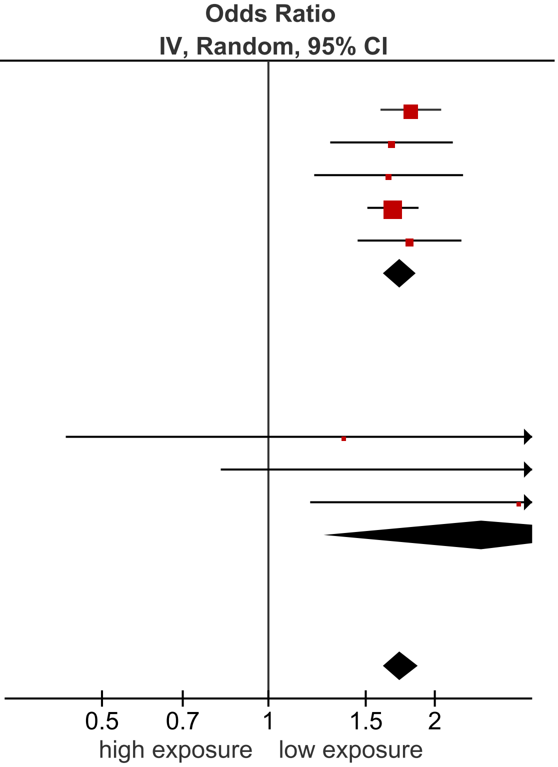

Study or Subgroup	log[Odds Ratio]	SE	Weight	Odds Ratio IV, Random, 95% CI
**1.1.1 Men**				
Bültmann	0.4121	0.1041	10.3%	1.51 [1.23, 1.85]
Magnusson Hanson	0.7372	0.1625	6.2%	2.09 [1.52, 2.87]
Oshio 2017 man	0.4824	0.0566	15.1%	1.62 [1.45, 1.81]
Stansfeld	0.2852	0.0956	11.1%	1.33 [1.10, 1.60]
**Subtotal (95% CI)**			**42.8%**	**1.57 [1.36, 1.80]**
Heterogeneity: Tau² = 0.01; Chi² = 6.56, df = 3 (P = 0.09); I² = 54%				
Test for overall effect: Z = 6.31 (P < 0.00001)				
**1.1.2 Women**				
Bültmann	0.3646	0.1706	5.8%	1.44 [1.03, 2.01]
Magnusson Hanson	0.5822	0.1402	7.5%	1.79 [1.36, 2.36]
Oshio 2017 vrouw	0.5365	0.1094	9.8%	1.71 [1.38, 2.12]
Stansfeld	0.2151	0.1199	9.0%	1.24 [0.98, 1.57]
**Subtotal (95% CI)**			**32.2%**	**1.53 [1.29, 1.82]**
Heterogeneity: Tau² = 0.01; Chi² = 5.48, df = 3 (P = 0.14); I² = 45%				
Test for overall effect: Z = 4.77 (P < 0.00001)				
**1.1.3 All**				
Andersen 2017	0.4762	0.1457	7.2%	1.61 [1.21, 2.14]
Devereux	0.4824	0.1282	8.3%	1.62 [1.26, 2.08]
Mino	0.2231	0.1319	8.1%	1.25 [0.97, 1.62]
Sundin	1.4656	0.3992	1.4%	4.33 [1.98, 9.47]
**Subtotal (95% CI)**			**25.0%**	**1.65 [1.24, 2.21]**
Heterogeneity: Tau² = 0.05; Chi² = 9.46, df = 3 (P = 0.02); I² = 68%				
Test for overall effect: Z = 3.40 (P = 0.0007)				
**Total (95% CI)**			**100.0%**	**1.56 [1.41, 1.72]**
Heterogeneity: Tau² = 0.01; Chi² = 21.60, df = 11 (P = 0.03); I² = 49%				
Test for overall effect: Z = 8.94 (P < 0.00001)				
Test for subgroup differences: Chi² = 0.20, df = 2 (P = 0.91), I² = 0%				

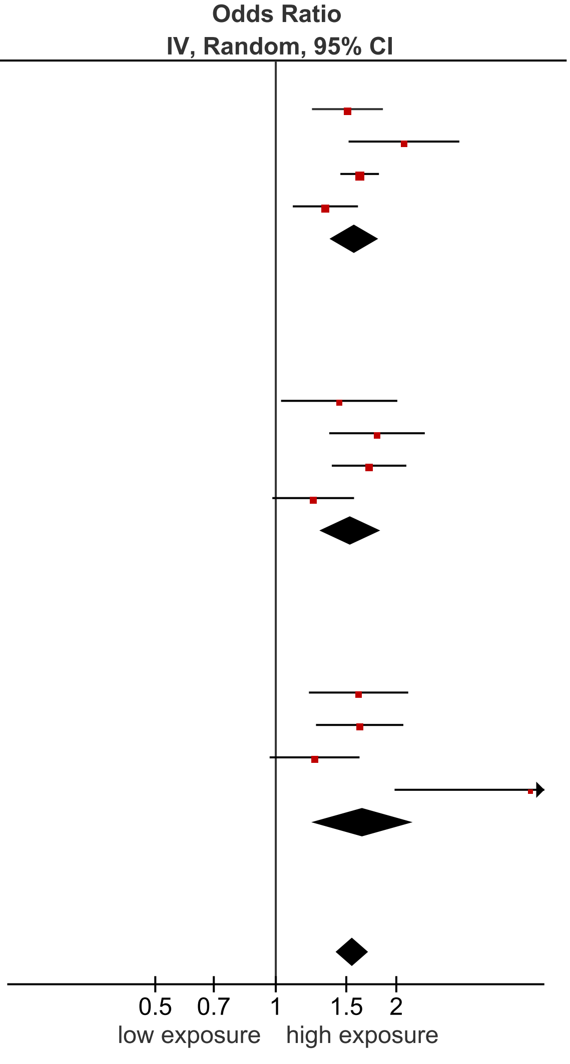

Appendix Search strategy Psychosocial risk factors and SRDs 12-08-2019
==
Medline
==
1. exp Occupations/ or Workload/ or exp Work/ or Workplace/ or exp Occupational Diseases/ or Rehabilitation, Vocational/ or Occupational Health/ or Sick Leave/ or Absenteeism/ or Retirement/ or workers' compensation/ or exp Employment/ or exp Occupational Exposure/ or Volunteers/
2. (worka* or worke* or workg* or worki* or workl* or workp* or work capacity or work disabilit* or work abilit* or at work or work exposure or work related or workers or job* or employee or staff or personnel or occupation or occupations or occupational or outdoor work* or day shift* or night shift* or shift work* or vocational rehabilitation or sick leave or absenteeism or sickness absen* or absente* or presente* or return to work or vocational reintegration or retirement or pension or employment or unemployed or unemployment or work status or industries or volunteer* or voluntary worker*).ab,kw,ti.
3. or/1-2 [working population]
4. risk/ or risk assessment/ or risk factors/ or cohort studies/ or Follow-Up Studies/
5. (risk* or Case control or cohort stud* or Cohort analy* or follow up stud* or observational stud* or Longitudinal).ab,kw,ti.
6. or/4-5 [aetiology]
7. Burnout, Professional/ or neurasthenia/ or adjustment disorders/
8. (burnout or burn out or neurasthenia or adjustment disorder? or emotional exhaustion or job stress or work stress or occupational stress or job strain or work strain).ab,kw,ti.
9. (occupational health/ or occupational disease/) and (psychiatric disorder? or psychiatric morbidity or psychosocial or psycho social or occupational stress or distress).ab,kw,ti.
10. or/7-9 [burnout]
11. (stress symptom* or psychologic* stress or psychologic* distress or psychosocial risk* or psycho social risk* or psychosocial factor* or psycho social factor* or psychiatric morbidit* or psychiatric disorder*).ab,kw,ti. [stress related symptoms and disorders]
12. 3 and 6 and 10
13. ((worka* or worke* or workg* or worki* or workl* or workp* or work capacity or work disabilit* or work abilit* or at work or work exposure or work related or workers or job* or employee or staff or personnel or occupation or occupations or occupational or outdoor work* or day shift* or night shift* or shift work* or vocational rehabilitation or sick leave or absenteeism or sickness absen* or absente* or presente* or return to work or vocational reintegration or retirement or pension or employment or unemployed or unemployment or work status or industries or volunteer* or voluntary worker*) adj1 (stress symptom* or psychologic* stress or psychologic* distress or psychosocial risk* or psycho social risk* or psychosocial factor* or psycho social factor* or psychiatric morbidit* or psychiatric disorder*)).ab,kw,ti.
14. 6 and 13
15. ((risk* or Case control or cohort stud* or Cohort analy* or follow up stud* or observational stud* or Longitudinal) adj1 (stress symptom* or psychologic* stress or psychologic* distress or psychosocial risk* or psycho social risk* or psychosocial factor* or psycho social factor* or psychiatric morbidit* or psychiatric disorder*)).ab,kw,ti.
16. 3 and 15
17. 12 or 14 or 16
18. limit 17 to (dutch or english or german)

===
PsycINFO
===
1. (occupational employment testing or occupational interests guidance or occupational vocational rehabilitation or working conditions industrial safety).cc.
2. exp occupations/ or career change/ or career development/ or exp "division of labor"/ or employment history/ or exp job characteristics/ or occupational adjustment/ or occupational aspirations/ or occupational attitudes/ or occupational choice/ or occupational exposure/ or occupational guidance/ or occupational interests/ or occupational mobility/ or occupational preference/ or occupational safety/ or occupational status/ or occupational success/ or exp occupational tenure/ or exp personnel/ or exp professional personnel/ or working women/ or "work (attitudes toward)"/ or Workplace Violence/ or Diversity in the Workplace/ or exp working conditions/ or exp vocational rehabilitation/ or Occupational Health/ or employee absenteeism/ or Retirement/ or exp employment status/ or reemployment/ or Volunteers/
3. (worka* or worke* or workg* or worki* or workl* or workp* or work capacity or work disabilit* or work abilit* or at work or work exposure or work related or workers or job* or employee or staff or personnel or occupation or occupations or occupational or outdoor work* or day shift* or night shift* or shift work* or vocational rehabilitation or sick leave or absenteeism or sickness absen* or absente* or presente* or return to work or vocational reintegration or retirement or pension or employment or unemployed or unemployment or work status or industries or volunteer* or voluntary worker*).ab,id,ti.
4. or/1-3 [working population]

For peer review only - http://bmjopen.bmj.com/site/about/guidelines.xhtml

5. Risk Assessment/ or At Risk Populations/ or Risk Factors/ or clinical trials/ or cohort analysis/ or followup studies/ or exp longitudinal studies/
6. (risk* or Case control or cohort stud* or Cohort analy* or follow up stud* or observational stud* or Longitudinal).ab,id,ti.
7. or/5-6 [aetiology]
8. occupational stress/ or work related illnesses/ or neurastenia/ or adjustment disorders/
9. (neurasthenia or burnout or burn out or emotional exhaustion or job stress or work stress or occupational stress or job strain or work strain or adjustment disorder?).ab,id,ti.
10. 8 or 9 [burnout]
11. (stress or distress).ab,id,ti.
12. 4 and 7 and 10
13. ((risk* or Case control or cohort stud* or Cohort analy* or follow up stud* or observational stud* or Longitudinal) adj3 (stress or distress)).ab,id,ti.
14. 4 and 13
15. 12 or 14
16. limit 15 to ("0100 journal" or "0110 peer-reviewed journal" or "0120 non-peer-reviewed journal" or "0130 peer-reviewed status unknown" or "0400 dissertation abstract")
17. limit 16 to (dutch or english or german)

==
Embase
==
1. exp *occupation/ or exp *occupational health/ or *work/ or *workplace/ or *"employment of women"/ or exp *income/ or *workman compensation/ or exp *employment/ or exp *"named groups by occupation"/
2. (worka* or worke* or workg* or worki* or workl* or workp* or work capacity or work disabilit* or work abilit* or at work or work exposure or work related or workers or job* or employee or staff or personnel or occupation or occupations or occupational or outdoor work* or day shift* or night shift* or shift work* or vocational rehabilitation or sick leave or absenteeism or sickness absen* or absente* or presente* or return to work or vocational reintegration or retirement or pension or employment or unemployed or unemployment or work status or industries or volunteer* or voluntary worker*).ab,kw,ti.
3. or/1-2 [working population]
4. *risk/ or *risk assessment/ or *risk factor/ or *cohort analysis/ or *Follow-Up/
5. (risk* or Case control or cohort stud* or Cohort analy* or follow up stud* or observational stud* or Longitudinal).ab,kw,ti.
6. or/4-5 [etiology]
7. *Burnout/ or *neurasthenia/ or *adjustment disorder/ or *emotional stress/ or *job stress/
8. (burnout or burn out or neurasthenia or adjustment disorder? or emotional exhaustion or job stress or work stress or occupational stress or job strain or work strain).ab,kw,ti.
9. (occupational health/ or occupational disease/) and (psychiatric disorder? or psychiatric morbidity or psychosocial or psycho social or occupational stress or distress).ab,kw,ti.
10. or/7-9 [burnout]
11. (stress symptom* or psychologic* stress or psychologic* distress or psychosocial risk* or psycho social risk* or psychosocial factor* or psycho social factor* or psychiatric morbidit* or psychiatric disorder*).ab,kw,ti. [stress related symptoms and disorders]
12. 3 and 6 and 10
13. ((worka* or worke* or workg* or worki* or workl* or workp* or work capacity or work disabilit* or work abilit* or at work or work exposure or work related or workers or job* or employee or staff or personnel or occupation or occupations or occupational or outdoor work* or day shift* or night shift* or shift work* or vocational rehabilitation or sick leave or absenteeism or sickness absen* or absente* or presente* or return to work or vocational reintegration or retirement or pension or employment or unemployed or unemployment or work status or industries or volunteer* or voluntary worker*) adj1 (stress symptom* or psychologic* stress or psychologic* distress or psychosocial risk* or psycho social risk* or psychosocial factor* or psycho social factor* or psychiatric morbidit* or psychiatric disorder*)).ab,kw,ti.
14. 6 and 11 and 13
15. ((risk* or Case control or cohort stud* or Cohort analy* or follow up stud* or observational stud* or Longitudinal) adj1 (stress symptom* or psychologic* stress or psychologic* distress or psychosocial risk* or psycho social risk* or psychosocial factor* or psycho social factor* or psychiatric morbidit* or psychiatric disorder*)).ab,kw,ti.
16. 3 and 15
17. 12 or 14 or 16
18. limit 17 to (dutch or english or german)

For peer review only - http://bmjopen.bmj.com/site/about/guidelines.xhtml

PRISMA 2009 Checklist

Section/topic	#	Checklist item	Reported on page #
**TITLE**			
Title	1	Identify the report as a systematic review, meta-analysis, or both.	1
**ABSTRACT**			
Structured summary	2	Provide a structured summary including, as applicable: background; objectives; data sources; study eligibility criteria, participants, and interventions; study appraisal and synthesis methods; results; limitations; conclusions and implications of key findings; systematic review registration number.	2
**INTRODUCTION**			
Rationale	3	Describe the rationale for the review in the context of what is already known.	5
Objectives	4	Provide an explicit statement of questions being addressed with reference to participants, interventions, comparisons, outcomes, and study design (PICOS).	5 and 8
**METHODS**			
Protocol and registration	5	Indicate if a review protocol exists, if and where it can be accessed (e.g., Web address), and, if available, provide registration information including registration number.	n/a
Eligibility criteria	6	Specify study characteristics (e.g., PICOS, length of follow-up) and report characteristics (e.g., years considered, language, publication status) used as criteria for eligibility, giving rationale.	6,7
Information sources	7	Describe all information sources (e.g., databases with dates of coverage, contact with study authors to identify additional studies) in the search and date last searched.	7,8
Search	8	Present full electronic search strategy for at least one database, including any limits used, such that it could be repeated.	Appendix 1
Study selection	9	State the process for selecting studies (i.e., screening, eligibility, included in systematic review, and, if applicable, included in the meta-analysis).	7,8,9,10
Data collection process	10	Describe method of data extraction from reports (e.g., piloted forms, independently, in duplicate) and any processes for obtaining and confirming data from investigators.	8
Data items	11	List and define all variables for which data were sought (e.g., PICOS, funding sources) and any assumptions and simplifications made.	8,9
Risk of bias in individual studies	12	Describe methods used for assessing risk of bias of individual studies (including specification of whether this was done at the study or outcome level), and how this information is to be used in any data synthesis.	8,9
Summary measures	13	State the principal summary measures (e.g., risk ratio, difference in means).	7
Synthesis of results	14	Describe the methods of handling data and combining results of studies, if done, including measures of consistency (e.g., I^2) for each meta-analysis.	9

For peer review only - http://bmjopen.bmj.com/site/about/guidelines.xhtml

PRISMA 2009 Checklist

Section/topic	#	Checklist item	Reported on page #
Risk of bias across studies	15	Specify any assessment of risk of bias that may affect the cumulative evidence (e.g., publication bias, selective reporting within studies).	9,10, table 3
Additional analyses	16	Describe methods of additional analyses (e.g., sensitivity or subgroup analyses, meta-regression), if done, indicating which were pre-specified.	n/a
**RESULTS**			
Study selection	17	Give numbers of studies screened, assessed for eligibility, and included in the review, with reasons for exclusions at each stage, ideally with a flow diagram.	11<br>Figure 1
Study characteristics	18	For each study, present characteristics for which data were extracted (e.g., study size, PICOS, follow-up period) and provide the citations.	Table 1
Risk of bias within studies	19	Present data on risk of bias of each study and, if available, any outcome level assessment (see item 12).	Table 2
Results of individual studies	20	For all outcomes considered (benefits or harms), present, for each study: (a) simple summary data for each intervention group (b) effect estimates and confidence intervals, ideally with a forest plot.	Table 1
Synthesis of results	21	Present results of each meta-analysis done, including confidence intervals and measures of consistency.	18-20<br>Figure 2.1-2.3
Risk of bias across studies	22	Present results of any assessment of risk of bias across studies (see Item 15).	Table 3
Additional analysis	23	Give results of additional analyses, if done (e.g., sensitivity or subgroup analyses, meta-regression [see Item 16]).	n.a.
**DISCUSSION**			
Summary of evidence	24	Summarize the main findings including the strength of evidence for each main outcome; consider their relevance to key groups (e.g., healthcare providers, users, and policy makers).	21,22
Limitations	25	Discuss limitations at study and outcome level (e.g., risk of bias), and at review-level (e.g., incomplete retrieval of identified research, reporting bias).	22,23
Conclusions	26	Provide a general interpretation of the results in the context of other evidence, and implications for future research.	24,25
**FUNDING**			
Funding	27	Describe sources of funding for the systematic review and other support (e.g., supply of data); role of funders for the systematic review.	25

From: Moher D, Liberati A, Tetzlaff J, Altman DG, The PRISMA Group (2009). Preferred Reporting Items for Systematic Reviews and Meta-Analyses: The PRISMA Statement. PLoS Med 6(7): e1000097. doi:10.1371/journal.pmed1000097

For peer review only - http://bmjopen.bmj.com/site/about/guidelines.xhtml

BMJ Open

Work-related psychosocial risk factors for stress-related mental disorders – an updated systematic review and meta-analysis

Journal:	*BMJ Open*
Manuscript ID	bmjopen-2019-034849.R2
Article Type:	Original research
Date Submitted by the Author:	11-May-2020
Complete List of Authors:	van der Molen, Henk; Amsterdam UMC - Locatie AMC, Coronel Institute of Occupational Health<br>Nieuwenhuijsen, Karen; Amsterdam UMC - Locatie AMC, Coronel Institute of Occupational Health<br>Frings-Dresen, Monique; Amsterdam UMC - Locatie AMC, Coronel Institute of Occupational Health<br>de Groene, Gerda; Amsterdam UMC - Locatie AMC, Coronel Institute of Occupational Health
**Primary Subject Heading**:	Occupational and environmental medicine
Secondary Subject Heading:	Epidemiology
Keywords:	OCCUPATIONAL & INDUSTRIAL MEDICINE, EPIDEMIOLOGY, PREVENTIVE MEDICINE

SCHOLARONE™ Manuscripts

I, the Submitting Author has the right to grant and does grant on behalf of all authors of the Work (as defined in the below author licence), an exclusive licence and/or a non-exclusive licence for contributions from authors who are: i) UK Crown employees; ii) where BMJ has agreed a CC-BY licence shall apply, and/or iii) in accordance with the terms applicable for US Federal Government officers or employees acting as part of their official duties; on a worldwide, perpetual, irrevocable, royalty-free basis to BMJ Publishing Group Ltd ("BMJ") its licensees and where the relevant Journal is co-owned by BMJ to the co-owners of the Journal, to publish the Work in this journal and any other BMJ products and to exploit all rights, as set out in our licence.

The Submitting Author accepts and understands that any supply made under these terms is made by BMJ to the Submitting Author unless you are acting as an employee on behalf of your employer or a postgraduate student of an affiliated institution which is paying any applicable article publishing charge ("APC") for Open Access articles. Where the Submitting Author wishes to make the Work available on an Open Access basis (and intends to pay the relevant APC), the terms of reuse of such Open Access shall be governed by a Creative Commons licence – details of these licences and which Creative Commons licence will apply to this Work are set out in our licence referred to above.

Other than as permitted in any relevant BMJ Author's Self Archiving Policies, I confirm this Work has not been accepted for publication elsewhere, is not being considered for publication elsewhere and does not duplicate material already published. I confirm all authors consent to publication of this Work and authorise the granting of this licence.

For peer review only

Work-related psychosocial risk factors for stress-related mental disorders – an updated systematic review and meta-analysis

Henk F. van der Molen[1], Karen Nieuwenhuijsen[1], Monique H.W. Frings-Dresen[1], Gerda de Groene[1]

[1]Amsterdam UMC, University of Amsterdam, Department: Coronel Institute of Occupational Health, University of Amsterdam, Netherlands Center for Occupational Diseases, Amsterdam Public Health research institute, PO Box 22660, 1100 DD Amsterdam, the Netherlands

[2] Corresponding author

h.f.vandermolen@amsterdamumc.nl

For peer review only - http://bmjopen.bmj.com/site/about/guidelines.xhtml

Abstract

Objective The objective was to conduct an update of a previously published review and meta-analysis on the association between work-related psychosocial risk factors and stress-related mental disorders (SRD).

Design Systematic review and meta-analysis

Data sources Medline, Embase and PsycINFO were searched for articles published between 2008 and 12 August 2019 and references of a systematic review performed for the period before 2008 were included. Primary prospective studies were included when outcome data were described in terms of SRD assessment or a dichotomous outcome, based on a validated questionnaire, and at least two levels of work-related exposure were reported (exposed versus less or non-exposed). We used GRADE to assess the evidence for the associations between risk factors and the onset of SRD.

Results

Seventeen studies met the inclusion criteria. In total, a population of 73,874 workers from Belgium, Denmark, England, Finland, Japan, the Netherlands and Sweden were included in the meta-analysis of fourteen prospective cohort studies. This meta-analysis revealed moderate evidence for associations between SRD and effort reward imbalance (OR=1.9, 95% CI 1.70-2.15), high job demands (OR=1.6, 95% CI 1.41-1.72), organisational justice (OR's 1.6 to 1.7, CI's 1.44-1.86), social support (OR's 1.3 to 1.4, CI's 1.16-1.69), high emotional demands (OR=1.6, 95% CI 1.35-1.84), and decision authority (OR=1.3, CI 1.20-1.49). No significant or inconsistent associations were found for job insecurity, decision latitude, skill discretion and bullying.

Conclusion

Moderate evidence was found that work-related psychosocial risk factors are associated with a higher risk of SRD. Effort-reward imbalance, low organisational justice and high job demands exhibited the largest increased risk of SRD, varying from 60 to 90%.

Keywords

Mental, Stress Related Disorders, Work, Risk Factors, Meta-analysis, Aetiology

For peer review only - http://bmjopen.bmj.com/site/about/guidelines.xhtml

Strengths and limitations of this study

► This systematic review, including a meta-analysis of prospective cohort studies, gives a valuable overview which work-related psychosocial risk factors increase the risk of stress related mental disorders.

► The case definitions of stress related mental disorders used validated questionnaires and the methodological quality of the included studies was high.

► The GRADE framework made it possible to assess to the quality of evidence, however, the starting level for grading this evidence for prospective etiologic studies is subject to scientific debate.

► Limitations are the lack of harmonized assessments of stress related mental disorders and detailed exposure assessment in the identified observational cohort studies.

For peer review only

For peer review only - http://bmjopen.bmj.com/site/about/guidelines.xhtml

Introduction

Stress related mental disorders (SRD) are frequently reported in the working population, with varying incidence rates of 13% for psychological distress [1], 22% for emotional exhaustion [2], and a prevalence of distress symptoms of up to 50% in specific professions and countries [3]. SRD represent a significant part of work-related common mental disorders in both self-report surveys and notification systems for occupational diseases [4]. The World Health Organization has recently announced [5] that burn-out is included as 'an occupational phenomenon' in the 11th revision of the International Classification of Diseases (ICD-11).

In many countries, SRD are not notified as occupational diseases [6], mainly because of their multifactorial origin [7] or discussion about the medical status (as with burn-out) [5,8,9]. Evidence-based knowledge about work-related factors could be used for facilitating a decision on the 'work-relatedness' of an individual case of SRD in a reporting scheme or concerning the selection and implementation of preventive workplace interventions around factors with the strongest effect on SRD [7].

In the Netherlands, SRD cover diagnoses of adjustment disorders (ICD-10: F43.2) and burn-out as a state of exhaustion (ICD-10: Z730) [4]. These diagnoses differ from just stress symptoms since they are clinically assessed and have in common that they reflect a status where subjective and emotional disturbance interfere with social functioning and performance, arising from a period of adaptation to stressful conditions. Symptoms are what a worker experiences, and these symptoms might negatively affect normal or regular functioning of a person – a so called disorder. To distinguish between symptoms and disorders, we considered a SRD as diagnosis following a clinical anamnesis, stress complaints as assessed in a score above a cut-off point on a validated questionnaire or absenteeism from work due to stress problems. Work-related psychosocial risk factors can contribute to the onset of SRD [10].

For peer review only - http://bmjopen.bmj.com/site/about/guidelines.xhtml

The high and increasing burden of SRD strengthens the urgency of up-to-date insight into work-related psychosocial risk factors associated with SRD and based on longitudinal study designs in order to initiate relevant primary and secondary preventive interventions at worksites. Previous systematic reviews aiming to determine work-related risk factors for SRD have either not been updated since 2008 [10], combined various common mental disorders, such as anxiety, depression and stress-related disorders, as outcome measures [11] or used other outcomes, such as suicide [12]. Based on seven prospective studies, Nieuwenhuijsen et al [10] reported that high job demands, low job control, low co-worker support, low supervisor support, low procedural justice, low relational justice, high effort–reward imbalance and job insecurity (only for men) predicted the occurrence of SRDs. Our aim is to update this previously published review and meta-analysis from 2008 [10] to examine: (1) which work-related psychosocial risk factors contribute to the onset of SRD and (2) to what extent these risk factors are associated with SRD.

Methods

This review followed the PRISMA Statement (see appendix). This review was not registered in Prospero, but an update of a previous review by Nieuwenhuijsen et al. [10].

Patient and public involvement

There was no patient or public involvement in designing the study, but the systematic review is a response to priority setting in collaboration with policy-makers of the Dutch Ministry of Employment and Social Affairs.

Study selection

Eligibility criteria

For peer review only - http://bmjopen.bmj.com/site/about/guidelines.xhtml

Primary prospective cohort studies were included where outcome data were described in terms of clinically or questionnaire-assessed stress-related disorder (present or not) and at least two levels of work-related exposure (exposed versus less or non-exposed) among a working population were reported in order to be able to retrieve or calculate a risk estimate (OR, RR, or HR).

This updated systematic review [10] considered studies to have included SRD if the outcome was either i) a SRD diagnosis following a clinical anamnesis, ii) a high level of stress complaints as assessed in a score above a cut-off point on a validated questionnaire for fatigue, stress or non-specific mental ill-health, or iii) absenteeism from work due to stress problems. Based on findings of Nieuwenhuijsen et al [10] and identified factors stemming from theoretical stress models, we defined seven types of exposure (i) job demands, (ii) job control (decision latitude, decision authority, skill discretion), (iii) social support (co-worker, supervisor, both), (iv) emotional demands, v) organisational justice (procedural and relational), (vi) effort-reward imbalance and (vii) other psychosocial risk factors, e.g. bullying.

Studies that described work-related risk factors in terms of psychosocial risk factors were eligible for inclusion. The studies had to describe workers in a real workplace setting. All types of exposure assessment were eligible for inclusion: self-reports or researcher observations or direct measurements. No additional criteria were formulated regarding latency between exposure and the presence or onset of the disorder or adjustment for confounders.

Data sources and search terms

We searched the electronic databases of Medline, Embase and PsychInfo for studies between 2008 and 12 August 2019 as described in supplement Appendix 1. Our PICO can be stated as:

For peer review only - http://bmjopen.bmj.com/site/about/guidelines.xhtml

P = working population, I/C exposed / less or none exposed to a-priori defined exposure categories, O = stress related disorder. Eligible studies before 2008 were retrieved from the systematic review by Nieuwenhuijsen et al. [10].

Data collection and analyses

Study selection process

Titles and abstracts were independently screened by two reviewers to identify potentially relevant studies. We used a free online software tool to screen and assess references (https://rayyan.qcri.org/welcome). The full texts of potentially relevant articles were assessed for eligibility against the inclusion criteria. Disagreement between review authors on the selection of studies for inclusion occurred in less than 5% of the references screened and was resolved by discussion.

Data extraction and management

Data were extracted by one review author (GG) and checked by another review author (HM). Data on the following was extracted from each article: author; country of study; study design; case definition of stress-related disorder; sources and number of participants; exposure definition; exposure assessment; exposure categories; risk estimate; and adjustment for confounders.

Methodological quality assessment

For the assessment of the risk of bias within studies, the quality criteria from the systematic review by Nieuwenhuijsen et al. [10] were used for all study designs, with nine items covering the selection of participants, measurement of variables and control for confounding. The quality of the studies was independently assessed and agreed by two review authors (GG, HM). In total,

For peer review only - http://bmjopen.bmj.com/site/about/guidelines.xhtml

nine items across five categories were evaluated for quality assessment: (1) the main features of the study population were stated e.g. sex, age and work context; (2) the participation rate at baseline was at least 50%; (3) the response at follow-up was at least 70% or the non-response was not selective; (4) the data on psychosocial work factors was collected using standardised instruments; (5) the data on outcome were collected at least at three different time points; (6) the statistical model used was appropriate for the outcome studied; (7) the measures of association with the statistical model were presented; (8) there were study controls for confounding with rationale; (9) the number of cases was at least 10 times the number of independent variables in the analysis. The criteria for each item were scored with 'positive', 'negative' or 'not clear'. A higher quality of a study was defined as ≥5 items scored as 'positive' out of the nine quality criteria.

Data synthesis

A descriptive analysis of all studies was performed, summarised, classified in categories of psychosocial risk factors, and assessed for methodological quality. Risk estimates and the corresponding 95% confidence intervals (CI) of the association between work-related factors and SRD were extracted and summarised. After assessing probable heterogeneity of risk factor and outcome assessment, all authors discussed and decided on the risk estimates to be included in the meta-analysis or should be analysed separately. We used the rule at least two studies when no statistical heterogeneity ($I^2 < 50\%$) and no heterogeneity in measures of risk factors and stress related disorders (as discussed among the authors) was found.

Meta-analyses and quality of evidence

The selection of the work-related risk factors in the meta-analyses was based on: (1) sufficient contrast between reported exposure categories, i.e. low versus high exposure; (2) effect

For peer review only - http://bmjopen.bmj.com/site/about/guidelines.xhtml

For peer review only

estimates controlled for other non-work-related factors, as reported in the primary studies; 3) homogeneity in definition or measurement of risk factors.

The meta-analysis was performed in line with the procedure described in Watenabe et al. [13]: by using a log-transformed OR and its SE in Review Manager (Cochrane Review Manager version 5.3). For the main analysis, the main ORs and the SEs from selected studies were subjected to a random-effects model meta-analysis to estimate a pooled OR and its 95% CI.

The quality of evidence - also taken into account the risk of bias across studies - was assessed using the GRADE (Grades of Recommendations, Assessment, Development and Evaluation) framework for prognostic studies developed by Huguet et al. [14]. Following Huguet et al. [14], our starting point for the quality of the evidence was 'moderate' for prospective cohort studies that aimed to identify associations between potential prognostic factors (in our review risk factors) and the outcome. The evidence could decrease on the basis of five factors: study limitations, inconsistency, indirectness, imprecision and publication bias. Moreover, two factors: (1) study findings with moderate or large effect sizes (i.e. lower limit of 95% CI OR≥2.0) or (2) an exposure-response gradient, could lead to an upgrade of the quality of evidence. Four levels of quality were used: high, moderate, low and very low.

Results

Selected studies

A PRISMA flow diagram of the study selection process is shown in Figure 1. After excluding duplicates, 10,801 references were retrieved from the databases and the systematic review by Nieuwenhuijsen et al. [10], and assessed based on title and abstract. The full texts of 139

For peer review only - http://bmjopen.bmj.com/site/about/guidelines.xhtml

potentially eligible articles were then examined, of which 17 studies from 16 articles met the inclusion criteria.

Figure 1 about here

Psychosocial risk factors and SRD

In total, seventeen longitudinal studies in sixteen articles [1-3, 15-27] described the association between exposure to psychosocial risk factors and the occurrence of assessed mental SRD. The characteristics of the outcome definition (SRD), exposure definition and reported associations are presented in table 1.

For peer review only

For peer review only - http://bmjopen.bmj.com/site/about/guidelines.xhtml

Table 1. Associations between psychosocial risk factors at work and the occurrence of mental stress related disorders (17 studies)

Author, Country	Outcome	Exposure		
**Population**	**Definition & assessment**	**Definition & assessment**	**OR**	**(95%CI)**
Mino 1999 [15]	General Health Questionnaire (GHQ-30)	Job demands: one item	♂ RR 1.39	0.92-2.10
Japan, machine	GHQ-30 ≥ 8	*(always/sometimes present vs absent)*	♀ RR 1.14	0.81-1.59
production, N=310		Supervisor support: one item	♂ RR 1.10	0.69-1.74
		(absent vs always/sometimes present)	♀ RR 2.21	1.25-3.89
Stansfeld 1999 [16]	General Health Questionnaire (GHQ-30)	Job demands: adapted JCQ	♂ 1.33	1.1-1.6
England, civil servants,	GHQ-30 > 4	*(highest tertile vs lowest)*	♀ 1.24	1.0-1.6
N=10,308		Decision authority: adapted JCQ	♂ 1.29	1.1-1.5
		(*lowest vs highest tertile*)	♀ 1.37	1.1-1.8
		Co-worker support: adapted JCQ	♂ 1.29	1.1-1.5
		(*lowest vs highest tertile*)	♀ 1.12	0.9-1.4
		Supervisor support: adapted JCQ	♂ 1.31	1.1-1.5
		(*lowest vs highest tertile*)	♀ 1.11	0.9-1.3
		Effort-reward imbalance: indicator of high effort and low rewards	♂ 2.57	1.8-3.6
		(high efforts/low rewards vs no high efforts/low rewards)	♀ 1.67	1.0-2.9
		Skill discretion: adapted JCQ	♂ 1.11	0.9-1.3
		(*lowest vs highest tertile*)	♀ 1.09	0.8-1.4
Bültmann 2002 [1]	General Health Questionnaire	Job demands Questionnaire (JCQ)		
Netherlands, 45	GHQ-12 ≥ 4	Job demands: JCQ	♂ 1.51	1.23-1.85
companies, N=8,833		(*highest vs lowest tertile*)	♀ 1.44	1.03-2.01
		Decision latitude: JCQ	♂ 1.14	0.9-1.43
		(*lowest vs highest tertile*)	♀ 0.88	0.62-1.24
		Co-worker support: JCQ	♂ 1.25	1.04-1.49
		(low vs high)	♀ 1.31	0.97-1.78
		Supervisor support: JCQ	♂ 1.25	1.05-1.49
		(low vs high)	♀ 1.12	0.85-1.47
		Emotional demands: Dutch Questionnaires; Perception and Judgement of	♂ 1.73	1.40-2.14
		Work (QPJW), Work and Health, self-formulated *(high (2-5) vs no (0,1)*	♀ 1.39	1.01-1.91
		Job insecurity: QPJW, one-item	♂ 1.63	1.18-2.27
		(*yes vs no*)	♀ 0.94	0.56-1.59
Bonde 2005 [17]	Setterlind Stress Profile Inventory (SPI)	Skill discretion: Repetitive work	1.3	0.6-2.2

For peer review only

Denmark, various workplaces, N=2,846	SSPI ≥ 4	*(yes vs no)*		
Godin 2005 [18]<br>Belgium, privat and public sector, N=1,521	Short Fatigue Inventory (SHI)<br>SHI: upper quartile	Effort-Reward Imbalance Questionnaire<br>*(highest quartile vs rest)*	♂ 3.4<br>♀ 2.0	1.7-6.7<br>0.9-4.1
Kivimaki 2007 [19]<br>Finland, government<br>N=21,271	General Health Questionnaire<br>GHQ-12 ≥ 4	Effort-Reward Imbalance Questionnaire<br>*(highest vs lowest quartile)*<br>Procedural & organisational injustice: Organizational Justice Scale<br>*(highest vs lowest quartile)*<br>Relational injustice: Organizational Justice Scale<br>*(highest vs lowest quartile)*	2.04<br><br>1.81<br><br>1.50	1.80-2.32<br><br>1.60-2.06<br><br>1.32-1.70
Kivimaki 2007 [19]<br>Finland, hospital<br>N=10,736	General Health Questionnaire<br>GHQ-12 ≥ 4	Effort-Reward Imbalance Questionnaire<br>*(highest vs lowest quartile)*<br>Procedural injustice: Organizational Justice Questionnaire (OJQ)<br>*(highest vs lowest quartile)*<br>Relational injustice: OJQ *(highest vs lowest quartile)*	1.59<br><br>1.67<br><br>1.56	1.24-2.05<br><br>1.29-2.15<br><br>1.21-2.02
M- Hanson 2008 [2]<br>Sweden, various workers, N=3,004	Maslach Burnout Inventory-general Survey MBI-GS ≥ 75th percentile	Job demands: Swedish Work Environment Survey (SWES)<br>*(≥2 positive vs < 2 out of four items)*<br>Decision authority: SWES<br>*(≥2 positive vs < 2 out of four items)*<br>Co-worker support: SWES; one item<br>*(4-point Likert scale, dichotomized: low (1,2) vs high (3.4))*<br>Supervisor support: SWES; one item<br>*(4-point Likert scale dichotomized: low (1,2) vs high (3.4))*	♂ 2.09<br>♀ 1.79<br>♂ 1.36<br>♀ 1.41<br>♂ 1.45<br>♀ 1.92<br>♂ 1.65<br>♀ 1.22	1.52-2.88<br>1.36-2.35<br>0.98-1.88<br>1.07-1.86<br>0.97-2.17<br>1.25-2.93<br>1.19-2.31<br>0.91-1.65
Devereux 2011 [20]<br>England, 11 industrial types, N=1,463	General Health Questionnaire (GHQ-12)<br>GHQ-12 ≥ 3	Job demands questionnaire: 4 items<br>(*highest vs lowest tertile*)<br>Decision latitude: 15 items<br>(*lowest vs highest tertile*)<br>Co-worker and supervisor support: 7 items<br>(*lowest vs highest tertile*)	RR 1.62<br><br>RR 1.11<br><br>RR 1.47	1.26-2.09<br><br>0.86-1.42<br><br>1.18-1.84
Sundin 2011 [21]<br>Sweden, nurses, N=555	Maslach Burnout Inventory (MBI)<br>Swedish version, Emotional Exhaustion (EE)	Job demands: Swedish Work Environmental Survey (SWES), one item, 5-point Likert scale *(at least once a week or more vs ≤1 day out of 10)*<br>Co-worker support: SWES; one item, 4-point Likert scale	4.33<br><br>2.21	1.98-9.45<br><br>0.88-5.56

For peer review only

	EE ≥ 27	*(less (1,2,3) vs always receiving support (4))*<br>Supervisor support: SWES; one item, 4-point Likert scale<br>*(less (1,2,3) vs always receiving support (4))*	 2.17	 0.65-7.26
Inoue 2013 [3]<br>Japan, manufacturing, N=569	Kessler Psychological Distress Scale (K6)<br>≥5 K6 scale	Procedural injustice: Organizational Justice Questionnaire (OJQ): 7 items, 5 point scale; Permanent (P) non-permanent worker (NP)<br>*(highest vs lowest tertile)*<br>Relational injustice: OJQ interactional justice 6-items, 5 point scale<br>*(highest vs lowest tertile)*	♂ 1.37 (P)<br>♀ 5.25 (P)<br>♀ 2.84 (NP)<br>♂ 2.39 (P)<br>♀ 2.35 (P)<br>♀ 1.61 (NP)	0.43-4.34<br>0.82-33.6<br>1.19-6.75<br>0.61-9.39<br>0.51-10.9<br>0.78-3.30
Laine 2014 [22]<br>Finland, public sector, N=3,298	General Health Questionnaire GHQ-12<br>≥3 GHQ-12	Procedural injustice: 4 items Moorman's inventory<br>*(highest vs lowest quartile)*<br>Relational injustice: 4 items Moorman's inventory<br>*(highest vs lowest quartile)*<br>Bullying: item about mental violence or workplace bullying *(yes vs no)*	1.65<br><br>1.29<br><br>1.57	1.21-2.24<br><br>0.97-1.72<br><br>0.97-2.53
Taniguchi 2015 [23]<br>Japan, elderly facilities, N=543	Brief Job Stress Questionnaire (BJSQ)<br>BJSQ > 13 (♂) or >12 (♀)	Bullying: Japanese Negative Acts Questionnaire (NAQ), person-related bullying (PR; 6 items), work-related bullying (WR; 3 items), sexual harassment (SH; 3 items), five-point scale. *(≥1 item positive versus none)*	3.46 (PR)<br>2.85 (WR)<br>1.73 (SH)	1.49-8.05<br>0.61-13.26<br>0.98-3.08
Andersen 2017 [24]<br>Denmark, prison personnel, N=1741	Copenhagen Burnout Inventory (COPSOQ-CBI)<br>COPSOQ-CBI highest quartile	Copenhagen Psychosocial Questionnaire (COPSOQ), 5-point Likert-scale (*most exposed quartile vs least exposed three quartiles)*<br>Job demands: 4 items<br>Co-worker and supervisor support: 6 items<br>Emotional demands: 4 items<br>Effort-reward imbalance (low recognition): 3 items	 <br>1.61<br>1.31<br>1.46<br>1.33	 <br>1.21-2.16<br>0.98-1.76<br>1.06-2.01<br>0.98-1.80
Oshio 2017 [25]<br>Japan, 12 industrial types, N=7419	Kessler Psychological Distress Scale (K6)<br>K6 ≥ 5	Job demands: 5 items Japanese Job Content Questionnaire (JCQ), 4-point scale *(median-split method classifying high vs low)*<br>Procedural injustice: Japanese version of Organisational Justice Questionnaire (OJQ), 7 items, 5-point Likert scale<br>Relational injustice: Japanese version of Organisational Justice Questionnaire (OJQ), 6 items, 5-point Likert scale<br>Effort reward imbalance: Japanese version Effort-Reward Imbalance Questionnaire, effort (3 items), reward (7 items), 4-point scale	♂ 1.62<br>♀ 1.71<br>♂ 1.68<br>♀ 1.80<br>♂ 1.58<br>♀ 1.71<br>♂ 2.05<br>♀ 1.84	1.45-1.81<br>1.38-2.13<br>1.51-1.87<br>1.45-2.23<br>1.42-1.77<br>1.37-2.13<br>1.84-2.29<br>1.48-2.28
Kind 2018 [26]	Burnout screening scales (BOSS)	Workplace aggression: exposure to verbal and physical threats		

For peer review only

Switzerland, youth welfare, N=121	BOSS T-score ≥60	*Verbal aggression vs no aggression*	HR 1.67	1.09-2.58
P-Thingvad 2019 [27] Denmark, social educators, N=1823	Copenhagen Burnout Inventory (CBI) CBI ≥ 75th percentile	Workplace violence: Scandinavian checklist, 4 items, 4-point Likert-scale *(high exposed (≥1 incident per month) vs non- exposed)*	1.4	0.9-2.3

For peer review only

For peer review only - http://bmjopen.bmj.com/site/about/guidelines.xhtml

Table 2. Methodological quality scores of 9 items for studies regarding risk factors*										
**Author**	**1**	**2**	**3**	**4**	**5**	**6**	**7**	**8**	**9**	**Summary score**
Mino 1999	-	+	-	-	+	+	+	- [a]	+	5
Stansfeld 1999	-	+	+	-	+	+	+	- [b]	+	6
Bültmann 2002	+	-	+	+	-	+	+	- [c]	+	6
Bonde 2005	+	+	+	-	+	+	+	+[d]	+	8
Godin 2005	+	-	+	+	-	+	+	- [e]	+	6
Kivimaki 10-town study 2007	+	+	+	-	-	+	+	- [f]	+	6
Kivimaki hospital study 2007	+	+	+	+	-	+	+	- [f]	+	7
Magnusson Hanson 2008	+	+	-	-	-	+	+	- [g]	+	5
Devereux 2011	+	?	-	+	-	+	+	- [h]	+	5
Sundin 2011	+	+	-	+	-	+	+	+[i]	+	7
Inoue 2013	+	+	+	+	-	+	+	+[j]	+	8
Laine 2014	-	+	+	+	-	+	+	+[k]	+	7
Taniguchi 2015	+	+	-	+	-	+	+	+[l]	+	7
Andersen 2017	+	+	-	+	-	+	+	+[m]	+	7
Oshio 2017	+	+	+	+	-	+	+	+[n]	+	8
Kind 2018	+	-	+	+	+	+	+	+[0]	+	8
Pihl-Thingvad 2019	+	+	+	+	-	+	+	+[p]	+	8
**Total item score**	14	13	11	12	4	17	17	9	17	

* Criteria and scoring options for quality score for these 9 items are reported in the methods section

[a]Adjusted for age, sex, family life satisfaction, perceived physical health

[b]Adjusted for age, sex, employment grade and baseline GHQ score

[c]Adjusted for age, sex, education, living alone, employment status, presence of disease, baseline fatigue score

[d]Adjusted for age, sex, body mass index, leisure time activity, pain threshold, marital status, psychiatric disorder

[e]Adjusted for age, sex, education, threat from global economy, job dissatisfaction, workplace instability

[f]Adjusted for age, sex, occupational status

[g]Adjusted for age, sex, marital status, country of birth, social class, physical exhaustion

[h]Adjusted for age, sex, shift work

[i]Adjusted for age, sex, marital status, years of (current) employment (only for job demands)

[j]Adjusted for age, sex, education, marital status, chronic physical diseases, occupation, life events, neuroticism

[k]Adjusted for age, sex, socioeconomic position, marital status, employment, health behaviour, limiting longstanding illness, physical work

[l]Adjusted for age, sex, job carrier, occupation, marital status, employment, work shift, smoking status

[m]Adjusted for age, sex, marital status, employment years, occupational characteristics and exposures

[n]Adjusted for age, sex, education, occupation, hours worked per week, household income, family member to share living expenses, firm codes

[o]Adjusted for age, sex, work experience, employment years, private stressors

[p]Adjusted for age, sex, somatic and mental health at baseline, lifestyle factors, work-related factors

For peer review only

Table 3. Quality of the evidence for the relationship between risk factors and stress related disorders according to the GRADE framework

	**Number of participants**	**Number of studies / cohorts**	**Study phase***	**Study limitations**<br><br>**Study quality majority of studies < 11/16: ↓**	**Inconsistency**<br><br>**$I^2 \geq 50\%$: ↓**	**Indirectness**<br><br>**Yes: ↓**	**Imprecision**<br><br>**Confidence interval (CI) effect size (<1 and >2) Yes: ↓**	**Publication Bias**<br><br>**Yes: ↓**	**Effect size OR (95% CI)**<br><br>**Lower Limit OR ≥ 2.0 : ↑**	**Exposure-response gradient (dose-effect)**<br><br>**Majority of studies: ↑**	**Overall Quality of Evidence**
Effort-reward imbalance	76,760	6/6	1	no	48%	no	no	no	1.91 (1.70-2.15)	2/6	Moderate
Low procedural justice	64,676	5/5	1	no	0%	no	no	no	1.74 (1.62-1.86)	2/5	Moderate
Low relational justice	64,676	5/5	1	no	0%	no	no	no	1.55 (1.44-1.67)	2/5	Moderate
High job demands	41,397	8/8	1	no	49%	no	no	no	1.56 (1.41-1.72)	0/8	Moderate
Low co-worker support	22,920	4/4	1	no	10%	no	no	no	1.29 (1.17-1.43)	0/4	Moderate
Low supervisor support	23,382	5/5	1	no	0%	no	no	no	1.27 (1.16-1.38)	0/5	Moderate
Low co-worker and supervisor support	7,262	2/2	1	no	0%	no	no	no	1.41 (1.18-1.69)	0/2	Moderate
High emotional demands	13,641	2/2	1	no	0%	no	no	undetected	1.58 (1.35-1.84)	0/2	Moderate
Low decision authority	13,312	2/2	1	no	0%	no	no	undetected	1.34 (1.20-1.49)	0/2	Moderate
Job insecurity	8,833	1/1	1	no	-	no	yes: ↓	undetected	Men 1.63 (1.18-2.27) Women 0.94 (0.56-1.59)	0/1	Low
Decision latitude	11,287	2/2	1	no	0%	no	no	undetected	1.07 (0.92-1.25)	0/2	Moderate
Low skill discretion	3,123	2/2	1	no	0%	no	no	undetected	1.11 (0.94-1.32)	0/2	Moderate
Bullying & violence	9,694	4/4	1	no	Heterogeneity in definitions	no	yes: ↓	no	1.42 – 3.64 (0.42-15.92)	0/4	Low

* All included studies are phase 1 explanatory prospective cohort studies with 'moderate' as starting point for the quality of the evidence

For peer review only

Methodological quality

The methodological quality of the studies of risk factors varied from scoring 5 out of 9 items to 8 out of 9 items (see table 2). The most frequently missing quality items were: response at follow-up less 70%, data on outcome were collected at least at three different time points, and study controls for confounding with rationale.

Meta-analyses and assessment of evidence

In total, twelve psychosocial risk factors from 14 studies [1-3, 15-22, 24,25] from a population of 73,874 workers from Belgium, Denmark, England, Finland, Japan, Netherlands and Sweden were used in the meta-analysis. Reported distress prevalence varied from 4.9% [17] to 45.8% [3]. The follow-up time varied from one to five years. Table 3 summarises the assessment of evidence concerning the psychosocial risk factors for stress-related disorders.

Effort-reward balance

Six cohort studies [16,18,19,24,25] demonstrate that there is moderate-quality evidence that effort-reward imbalance increases the incidence of SRD, with a pooled OR of 1.91 (95% CI 1.70-2.15) (see table 3 and figure 2). No statistically significant difference between subgroups of men and women were found [16,18,25].

Procedural and relational justice

Five cohort studies [3,19,22,25] demonstrate that there is moderate-quality evidence that low procedural justice with a pooled OR of 1.73 (95% CI 1.61-1.86) and low relational justice with a pooled OR of 1.55 (95%CI: 1.44-1.67) increase the incidence of SRD (see table 3 and figure 3). No statistically significant difference between subgroups of men and women were found [3,25].

For peer review only - http://bmjopen.bmj.com/site/about/guidelines.xhtml

Job demands

Eight cohort studies [1,2,15,16,20,21,24,25] demonstrate that there is moderate-quality evidence that high job demands increases the incidence of SRD, with a pooled OR of 1.55 (95% CI 1.41-1.71) (see table 3 and figure 4). No statistically significant difference between subgroups of men and women were found [1,2,15,16,25].

Co-worker and supervisor support

In total, five cohort studies demonstrate that there is moderate-quality evidence that low co-workers support [1,2,16,21] with a pooled OR 1.29 (95%CI: 1.17-1.43) and low supervisor support [1,2,15,16,21] with a pooled OR 1.27 (95%CI: 1.14-1.40) increase the incidence of SRD. No statistically significant difference between subgroups of men and women were found [1,215,16]. The combination of low co-worker and supervisor support [20,24] resulted in a pooled OR 1.41 (95%CI 1.18-1.69). (see table 3).

Emotional demands

Two cohort studies [1,24] demonstrate that there is moderate-quality evidence that high emotional demands increase the incidence of SRD, with a pooled OR of 1.58 (95% CI 1.35-1.84) (see table 3). No statistically significant difference between men and women were found in the study of Bültmann et al. 2002 [1].

Decision authority

Two cohort studies [2,16] demonstrate that there is moderate-quality evidence that low decision autonomy increases the incidence of SRD with a pooled OR of 1.34 (95% CI 1.20-

For peer review only - http://bmjopen.bmj.com/site/about/guidelines.xhtml

1.49) (see table 3). No statistically significant difference between subgroups of men and women were found.

Job security, decision latitude, skill discretion and bullying

One cohort study [1] demonstrates that there is low-quality evidence that job insecurity increases the incidence of SRD, with a pooled OR of 1.63 (95% CI 1.18-2.27) for male and no increase of the incidence for female with a pooled OR of 0.94 (95% CI 0.56-1.59). Two cohort studies [1,20] demonstrate that there is moderate-quality evidence for no increased incidence of SRD due to low decision latitude, with an OR of 1.07 (95% CI 0.92-1.25). Two cohort studies [16, 17] demonstrate that there is moderate-quality evidence for no increased incidence of SRD due to low skill discretion, with an OR of 1.11 (95% CI 0.94-1.32). Four cohort studies [22,23,26,27] demonstrate very low-quality evidence that there is inconsistent evidence for increased incidence of SRD with different types of bullying and workplace violence with ORs varying from 1.42 (95%CI 0.42-4.79) to 3.64 (95%CI 0.83-15.92). (see table 3). Laine et al. [22] studied workplace bullying or mental violence as the isolation of a member of the organisation, the underestimation of work performance, being threatened, being talked about behind one's back or other forms of pressure. Taniguchi et al. [23] studied person related bullying, workplace bullying and sexual harassment. Kind et al. [26] studied verbal and physical client aggression and Phil-Thingvad et al. [27] studied physical violence.

Figures 2 - 3 – 4 about here

For peer review only - http://bmjopen.bmj.com/site/about/guidelines.xhtml

DISCUSSION

Main findings

This systematic review, including a meta-analysis of prospective cohort studies revealed moderate-quality evidence that effort-reward imbalance, low procedural and relational justice, high job demands, low co-worker and supervisor support, high emotional demands, and low decision authority increase the incidence of stress-related disorders, varying from 20% to 90%. Low-quality evidence was found for an association between job insecurity and stress-related disorder among men. Moderate to low-quality evidence suggesting no associations were found for decision latitude, skill discretion and bullying.

Psychosocial risk factors and SRD

All prospective cohort studies were controlled for the personal factors of age and sex, and non-work-related factors such as education and marital status. Six studies also controlled for other work-related psychosocial risk factors in various models [3,19,20,22,27]. Regarding these studies, we decided not to take into account the fully adjusted model in the meta-analyses in order to avoid overcorrection, and strengthen transparency and comparability between studies. However, these models yielded small and non-statistically significant differences between the risk estimates.

All studies defined SRD based on self-report with validated and standardised questionnaires, i.e. (shortened) GHQ [1,15,16,19,20,22], Kessler 6 [3,25], MBI [2,21], COPSOQ-CBI [24,27], Setterlind Stress Inventory Profile [17], fatigue inventory [18], Brief job Stress Questionnaire (BJSQ) [27], burnout screening scale (BOSS) [26].

The pooled ORs of the meta-analyses of psychosocial risk factors were equal or slightly raised compared with the prior results of Nieuwenhuijsen et al. [10] and in line with the findings of a

For peer review only - http://bmjopen.bmj.com/site/about/guidelines.xhtml

recent meta-review of work-related risk factors for common mental-health problems including depression and anxiety [11].

Methodological considerations

The exposure categories for the risk factors and the case definitions of SRD varied in the included studies. We have explicitly described all definitions and assessments of self-reported exposures and outcomes (see table 1). In general, psychological distress can stem from private life as well as from work. Therefore, we only included high quality prospective studies aimed at identifying work-related risk factors, also controlling for other factors outside the work context. Although there is debate about the definition of burnout [5,7,28], we regarded distress and burnout as outcomes of the same continuum. We found that similar work-related psychosocial risk factors were associated with distress and the one-dimensional measures of burn-out ('exhaustion'). Should more studies using the multiple dimension definition of burnout become available, then subgroup analyses of these studies can be performed to assess whether the work-related risk factors are similar in this diagnostic subgroup.

We used the risk estimates - mostly ORs - as reported in the original prospective cohort studies (see also table 1). Meta-analyses were performed to estimate pooled associations, although the variety in measurement instruments and dichotomization rules introduced heterogeneity in the outcome measures (case definitions) and exposure categories of psychosocial risk factors. However, the exposure categories for the psychosocial risk factors included in the meta-analyses were already established in theoretical models of effort-reward imbalance [29], job demands-control [30] and organisational justice [31]. Furthermore, the case definitions of SRD used validated questionnaires and the methodological quality of the included studies was high and accompanied by a low to moderate statistical heterogeneity across the pooled studies (I^2 varying from 0% to 49%) which justify the meta-analyses. With the low number of studies for

For peer review only - http://bmjopen.bmj.com/site/about/guidelines.xhtml

each risk factor we feel that more subgroup analyses – besides the meta-analyses for separate risk factors and sexes – are not informative. Reporting bias is a concern because both psychosocial factors and SRD are self-reported in the included studies; it is conceivable that certain psychological states at baseline are associated both with the over-reporting of adverse working conditions at baseline and with the risk of onset of stress related disorders at follow-up [32]. In prospective studies this risk is less pronounced [33], however, in only four out 17 prospective studies in this systematic review data on outcome were collected at least at three different time points.

The GRADE framework made it possible to assess to the quality of evidence in reviews of prognostic factor research. However, the starting level for grading of the evidence for prospective etiologic studies is subject to scientific debate. For intervention studies, the grading of observational studies starts with low evidence. For prospective observational studies, which can answer prognostic, or as in our systematic review, etiological research questions, Huguet et al. [14] suggest that higher levels of evidence are derived from cohort studies that seeks to confirm independent associations between the prognostic factor and the outcome (high level of evidence) or – as in our systematic review – aimed to identify associations between potential prognostic factors and the outcome (moderate level of evidence). Also, recent GRADE guidelines state that best evidence regarding prognostic factors usually originates from observational studies (e.g. cohort studies or registries), while randomized control trials could include restrictions that exclude subjects relevant for the assessment of prognostic factors [34]. Based on Huguet et al. [14] we classified the risk of publication bias as 'no bias' when the value of the risk factor in predicting the outcome has been repetitively investigated in explanatory studies. We provided no funnel plots because the number of cohort studies for each psychosocial risk factor was substantially lower than 10. Visually checking the funnel plots,

For peer review only

however, suggested no serious publication bias or could not assessed [undetected publication bias].

For the risk factor, bullying and violence, it was decided not to perform a meta-analysis because of the different concepts of the psychosocial risk factor, i.e. person-related bullying, work-related bullying, sexual harassment, workplace bullying, threatening or violence.

Eventually, further research in harmonizing the assessment of SRD in cohort studies [7] and the clinical assessment with signs and symptoms of SRD [35], combined with more detailed or objective task-based exposure assessment [36] will contribute to a better insight into the work-related psychosocial risk factors and their mediating or moderating factors.

Prevention

To manage and prevent stress disorders it is necessary to identify which psychosocial risk factors at work contribute to the onset of SRD. West et al. [37] describe the importance of organisational strategies in their review on interventions to prevent and reduce SRD among physicians. Kalani et al. [38] report in their meta-review that various studies of the effectiveness of individually and organisationally directed interventions have led to different results on reducing physician burnout. Ultimately, to develop individually and organisationally directed interventions it is necessary that the relevant psychosocial risk factors in organisations are known [37,38].

Recently, Fan et al. [39] has suggested that some psychosocial factors such as job control, job security and social support are also associated with a greater likelihood of workers' experiencing positive mental well-being in terms of satisfaction and purpose in life, personal growth, social contribution and integration. This study implicates the double value of workplace policies and practices that improve psychosocial working conditions, reduce work-related SRD

For peer review only - http://bmjopen.bmj.com/site/about/guidelines.xhtml

and improve mental well-being in general, e.g. by giving workers greater job control or social support [39].

In conclusion, several psychosocial work-related risk factors for SRD were established, confirming the multifactorial aetiology of SRD. Effort-reward imbalance, low organisational justice and high job demands were associated with the largest increased risk of SRD, varying from 60 to 90%. Awareness of these risk factors could be the starting point for the selection of preventive interventions to reduce work-related SRD.

Acknowledgement

We are very grateful to Joost Daams for his contribution to the literature search and Nicolaas Kylstra and Kim van der Molen for their help with screening activities.

Competing interests

The authors declare that they have no competing interests.

Contributors

HM and GG initiated the study, performed data extraction and statistical analyses, and drafted the manuscript. All authors (HM, KN, MFD, GG) performed the study selection and interpretation of the data. All authors (HM, KN, MFD, GG) made substantial contributions to the conception of the study and manuscript. HM and GG equally contributed.

Funding

The work was sponsored by the Ministry of Social Affairs and Employment, the Netherlands. Grant number: 5100-24108

For peer review only - http://bmjopen.bmj.com/site/about/guidelines.xhtml

Competing interests None declared.

Patient consent for publication Not required.

Provenance and peer review Not commissioned; externally peer reviewed.

Data sharing statement No additional data available.

For peer review only

For peer review only - http://bmjopen.bmj.com/site/about/guidelines.xhtml

References

1. Bultmann U, Kant IJ, van den Brandt PA et al. Psychosocial work characteristics as risk factors for the onset of fatigue and psychological distress: prospective results from the Maastricht Cohort Study. *Psychol Med* 2002;32:333-345.
2. Magnusson Hanson LL, Theorell T, Oxenstierna G, Hyde M, Westerlund G. Demand, control and social climate as predictors of emotional exhaustion symptoms in working Swedish men and women. *Scand J Public Health* 2008; 36: 737-743.
3. Inoue A, Kawakami N, Tsuno K, Tomioka K, Nakanishi M. Organizational justice and psychological distress among permanent and non-permanent employees in Japan: a prospective cohort study. *Int J Behav Med* 2013; 20: 265-276.
4. van der Molen HF, de Vries S, Sluiter JK. Occupational diseases among workers in lower and higher socioeconomic positions. *Int J Environ Res Public Health* 2018;15(12):2849.
5. WHO. Burn-out an "occupational phenomenon": International Classification of DiseasesWhat are social determinants of health? 2019 https://www.who.int/mental_health/evidence/burn-out/en/?mc_cid=c8878361fa&mc_eid=8d4606394a (accessed 6 Aug 2019).
6. Lastovkova A, Cardner M, Rasmussen HM et al. Burnout syndrome as an occupational disease in the European Union: an exploratory study. *Ind Health* 2018;56(2):160-165.
7. Canu I, Bianchi R, Bubas M et al. Which factors contribute to the onset of burnout among workers? A systematic review. PROSPERO 2018 CRD42018105901 Available from: http://www.crd.york.ac.uk/PROSPERO/display_record.php?ID=CRD42018105901
8. Chirico F. Is burnout a syndrome or an occupational disease? Instructions for occupational physicians *Epidemiol Prev* 2017;41 (5-6):294-298.
9. Chirico F. The forgotten realm of the new and emerging psychosocial risk factors. *J Occup Health* 2017; 59(5):433-435.
10. Nieuwenhuijsen K, Bruinvels D, Frings-Dresen M. Psychosocial work environment and stress-related disorders, a systematic review. *Occup Med* (Lond) 2010;60:277-286
11. Harvey SB, Modini M, Joyce S et al. Can work make you mentally ill? A systematic meta-review of work-related risk factor for common mental health problems. *Occup Environ Med* 2017;74:301-310.
12. Milner A, Witt K, LaMontagne AD et al. Psychosocial job stressors and suicidality: a meta-analysis and systematic review. *Occup Environ Med* 2018;75:245-253.
13. Watanabe K, Imamura K, Kawakami N. Working hours and the onset of depressive disorder: a systematic review and meta-analysis. *Occup Environ Med* 2016;73(12):877-884.
14. Huguet A, Hayden J, Stinson J, McGrath P, Chambers C, Tougas M, Wozney L. Judging the quality of evidence in reviews of prognostic factor research: adapting the GRADE framework. *Syst Rev* 2013;2:71.
15. Mino Y, Shigemi J, Tsuda T et al. Perceived job stress and mental health in precision machine workers of Japan: a 2 year cohort study. *Occup Environ Med* 1999;56:41-45.
16. Stansfeld SA, Fuhrer R, Shipley MJ et al. Work characteristics predict psychiatric disorder: prospective results from the Whitehall II Study. *Occup Environ Med* 1999;56:302-307.
17. Bonde JP, Mikkelsen S, Andersen JH et al. Understanding work related musculoskeletal pain: does repetitive work cause stress symptoms? *Occup Environ Med* 2005;62:41-48.
18. Godin I, Kittel F, Coppieters Y et al. A prospective study of cumulative job stress in relation to mental health. *BMC Public Health* 2005;5:67.
19. Kivimaki M, Vahtera J, Elovainio M et al. Effort-reward imbalance, procedural injustice and relational injustice as psychosocial predictors of health: complementary or redundant models? *Occup Environ Med* 2007;64:659–665.

For peer review only

For peer review only - http://bmjopen.bmj.com/site/about/guidelines.xhtml

20. Devereux JJ, Rydstedt LW, Cropley M. Psychosocial work characteristics, need for recovery and musculoskeletal problems predict psychological distress in a sample of British Workers. *Ergonomics* 2011; 54(9),840-848.
21. Sundin L, Hochwälder J, Lisspers J. A longitudinal examination of generic and occupational specific job demands, and work-related social support associated with burnout among nurses in Sweden. *Work* 2011;38: 389-400.
22. Laine H, Saastamoinen P, Lahti J, Rahkonen O, Lahelma E. The associations between psychosocial working conditions and changes in common mental disorders: a follow-up study. *BMC Public Health* 2014;14-588.
23. Taniguchi T, Takaki J, Hirokawa K, Fujii Y, Harano K. Associations of workplace bullying and harassment with stress reactions: a two year follow-up study. *Ind Health* 2016,54,131-138.
24. Andersen D, Andersen L, Gadegaard C, Hogh A, Prieur A, Lund T. Burnout among Danish prison personnel: a Question of quantitative and emotional demands. *Scand J Public Health* 2017;45:824-830.
25. Oshio T, Inoue A, Tsutsumi A. Examining the mediating effect of work-to-family conflict on the associations between job stressors and employee psychological distress: a prospective cohort study. *BMJ Open* 2017;7:e015608.
26. Kind N, Eckert A, Steinlinc C, Fegert JM, Schmid M. Verbal and physical client aggression – A longitudinal analysis of professional caregivers' psychophysiological stress response and burnout. *Psychoneuroendocrinology* 2018;94:11-16.
27. Pihl‑Thingvad J, Elklit A, Brandt LPA, Andersen LL. Workplace violence and development of burnout symptoms: a prospective cohort study on 1823 social educators. *Int Arch Occ Env Hea* 2019;92:843-853.
28. Maslach C, Leiter MP. Understanding the burnout experience: recent research and its implications for psychiatry. *World Psychiatry* 2016;15(2):103-111.
29. Siegrist J, Siegrist K, Weber I. Sociological concepts in the etiology of chronic disease: the case of ischemic heart disease. *Soc Sci Med* 1986;22:247-253.
30. Karasek RA, Jr. Job demands, job decision latitude, and mental strain: implications for job design. *Admin Sci Q* 1979;24:285-308.
31. Moorman RH. Relationship between organizational justice and organizational citizenship behaviors: do fairness perceptions influence employee citizenship? *J Appl Psychol* 1991;76:845-855.
32. Rugulies R, Aust B, Madsen IEH. Effort–reward imbalance at work and risk of depressive disorders. A systematic review and meta-analysis of prospective cohort studies. *Scand J Work Environ Health* 2017;43(4):294-306.
33. Theorell T, Hammarström A, Aronsson G. A systematic review including meta-analysis of work environment and depressive symptoms. *BMC Public Health* 2015;15:738.
34. Foroutana F, Guyatt G, Zuka V et al. GRADE Guidelines 28: Use of GRADE for the assessment of evidence about prognostic factors: rating certainty in identification of groups of patients with different absolute risks. *Journal of Clinical Epidemiology* 2020;121:62-70.
35. Kakiashvili T, Leszek J, Rutkowski K The medical perspective on burnout. *Int J Occup Med Environ Health* 2013;26(3):401-12.
36. Göras C Olin K Unbeck M Pukk-Härenstam K. Tasks, multitasking and interruptions among the surgical team in an operating room: a prospective observational study. *BMJ Open* 2019;9(5):e026410.
37. West C, Dyrbye L, Erwin P, Shanafelt T. Interventions to prevent and reduce physician burnout: a systematic review and meta-analysis. *Lancet* 2016;388(10057):2272-2281.
38. Kalani S, Azadfallah P, Oreyzi H, Adibi P. Interventions for Physician Burnout: a systematic review of systematic reviews. *Int J Prev Med* 2018;9:81.
39. Fan JK, Mustard C, Smith PM. Psychosocial work conditions and mental health: examining differences across mental illness and well-being outcomes. *Annals of Work Exposures and Health* 2019;63 (5):546-559.

For peer review only - http://bmjopen.bmj.com/site/about/guidelines.xhtml

Figures, Tables & appendix

Figures

Figure 1 Prisma Flow Diagram

Figures 2 – 4 Forest Plots

Tables

Table 1. Associations between psychosocial risk factors at work and the occurrence of stress-related disorders (17 studies)

Table 2 Methodological quality scores of the studies including those concerning psychosocial risk factors

Table 3 Quality of the evidence of the relationship between psychosocial risk factors and stress-related disorders according to the GRADE framework

Appendix

Search of Medline, Embase and PsychInfo for studies between 2008 and 12 August 2019

For peer review only - http://bmjopen.bmj.com/site/about/guidelines.xhtml

Figure 1 PRISMA Flow Diagram

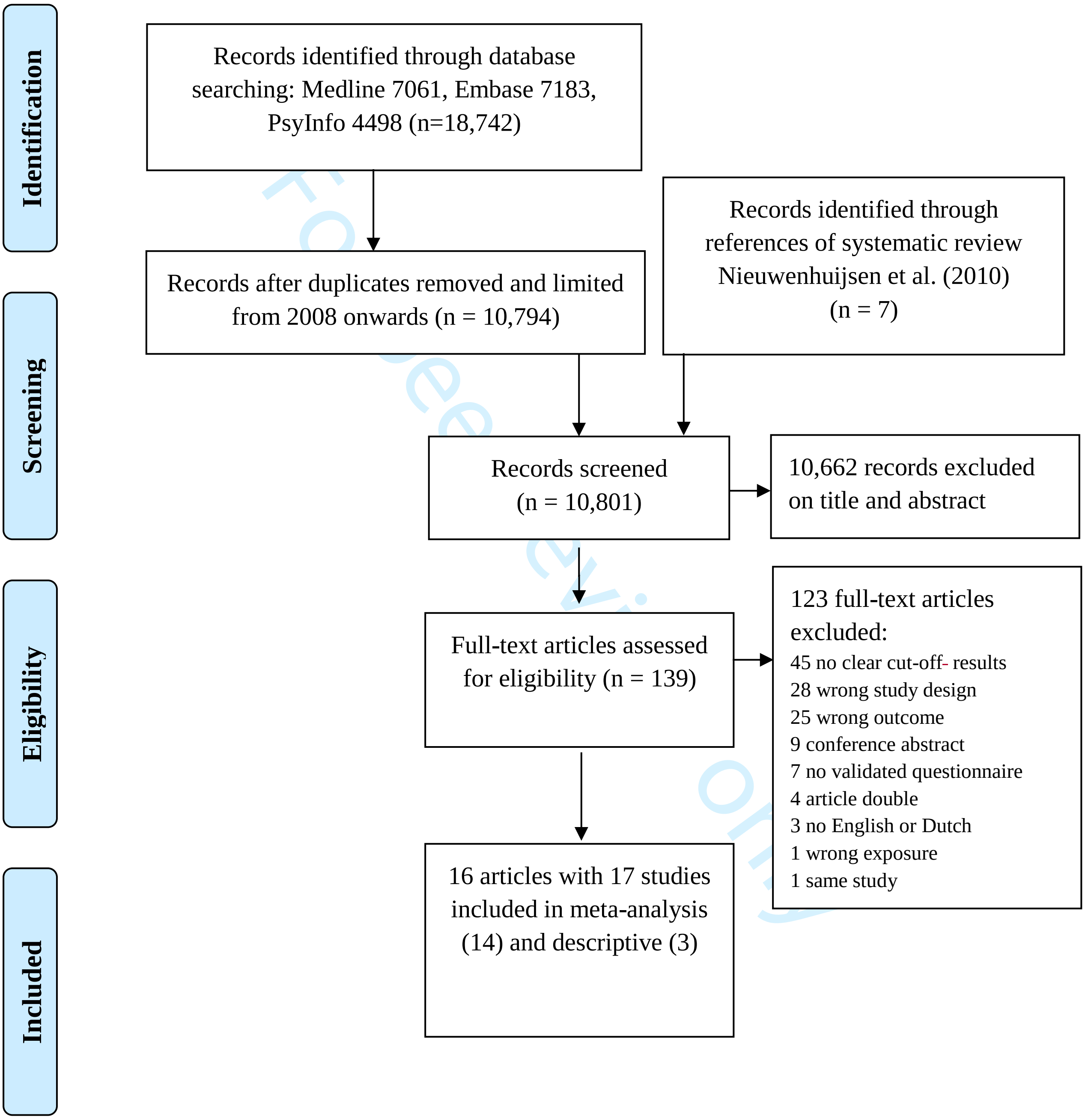

For peer review only - http://bmjopen.bmj.com/site/about/guidelines.xhtml

Study or Subgroup	log[Odds Ratio]	SE	Weight	Odds Ratio IV, Random, 95% CI
**7.1.1 men**				
Godin	1.2238	0.3537	2.7%	3.40 [1.70, 6.80]
Oshio 2017 man	0.7192	0.0558	23.3%	2.05 [1.84, 2.29]
Stansfeld	0.9439	0.1768	8.4%	2.57 [1.82, 3.63]
**Subtotal (95% CI)**			**34.3%**	**2.29 [1.82, 2.87]**
Heterogeneity: Tau² = 0.02; Chi² = 3.30, df = 2 (P = 0.19); I² = 39%				
Test for overall effect: Z = 7.16 (P < 0.00001)				
**7.1.2 women**				
Godin	0.6931	0.4074	2.1%	2.00 [0.90, 4.44]
Oshio 2017 vrouw	0.6081	0.1102	14.8%	1.84 [1.48, 2.28]
Stansfeld	0.5128	0.2716	4.3%	1.67 [0.98, 2.84]
**Subtotal (95% CI)**			**21.2%**	**1.82 [1.50, 2.21]**
Heterogeneity: Tau² = 0.00; Chi² = 0.16, df = 2 (P = 0.92); I² = 0%				
Test for overall effect: Z = 6.06 (P < 0.00001)				
**7.1.3 all**				
Andersen 2017	0.2838	0.1551	10.0%	1.33 [0.98, 1.80]
Kivimaki 2007a	0.7129	0.0647	21.8%	2.04 [1.80, 2.32]
Kivimaki 2007b	0.4637	0.1282	12.7%	1.59 [1.24, 2.04]
**Subtotal (95% CI)**			**44.5%**	**1.67 [1.29, 2.17]**
Heterogeneity: Tau² = 0.04; Chi² = 8.24, df = 2 (P = 0.02); I² = 76%				
Test for overall effect: Z = 3.87 (P = 0.0001)				
**Total (95% CI)**			**100.0%**	**1.91 [1.70, 2.15]**
Heterogeneity: Tau² = 0.01; Chi² = 15.50, df = 8 (P = 0.05); I² = 48%				
Test for overall effect: Z = 10.67 (P < 0.00001)				
Test for subgroup differences: Chi² = 3.64, df = 2 (P = 0.16), I² = 45.1%				

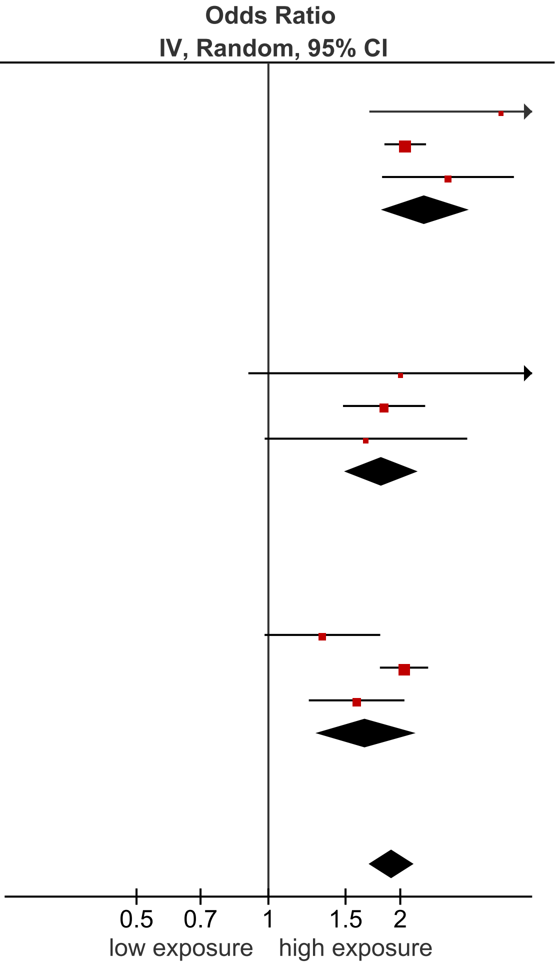

Study or Subgroup	log[Odds Ratio]	SE	Weight	Odds Ratio IV, Random, 95% CI
**5.1.1 All**				
Kivimaki 2007a	0.5933	0.0645	31.3%	1.81 [1.60, 2.05]
Kivimaki 2007b	0.5128	0.1303	7.7%	1.67 [1.29, 2.16]
Laine	0.5008	0.1582	5.2%	1.65 [1.21, 2.25]
Oshio 2017 man	0.5188	0.0544	44.0%	1.68 [1.51, 1.87]
Oshio 2017 vrouw	0.5878	0.1103	10.7%	1.80 [1.45, 2.23]
**Subtotal (95% CI)**			**98.8%**	**1.73 [1.61, 1.86]**
Heterogeneity: Tau² = 0.00; Chi² = 1.07, df = 4 (P = 0.90); I² = 0%				
Test for overall effect: Z = 15.11 (P < 0.00001)				
**5.1.2 P male, P female, NP female**				
Inoue	0.3148	0.5912	0.4%	1.37 [0.43, 4.36]
Inoue 2	1.6582	0.9473	0.1%	5.25 [0.82, 33.61]
Inoue 3	1.0438	0.4438	0.7%	2.84 [1.19, 6.78]
**Subtotal (95% CI)**			**1.2%**	**2.43 [1.27, 4.67]**
Heterogeneity: Tau² = 0.00; Chi² = 1.72, df = 2 (P = 0.42); I² = 0%				
Test for overall effect: Z = 2.67 (P = 0.007)				
**Total (95% CI)**			**100.0%**	**1.74 [1.62, 1.86]**
Heterogeneity: Tau² = 0.00; Chi² = 3.84, df = 7 (P = 0.80); I² = 0%				
Test for overall effect: Z = 15.31 (P < 0.00001)				
Test for subgroup differences: Chi² = 1.04, df = 1 (P = 0.31), I² = 3.6%				

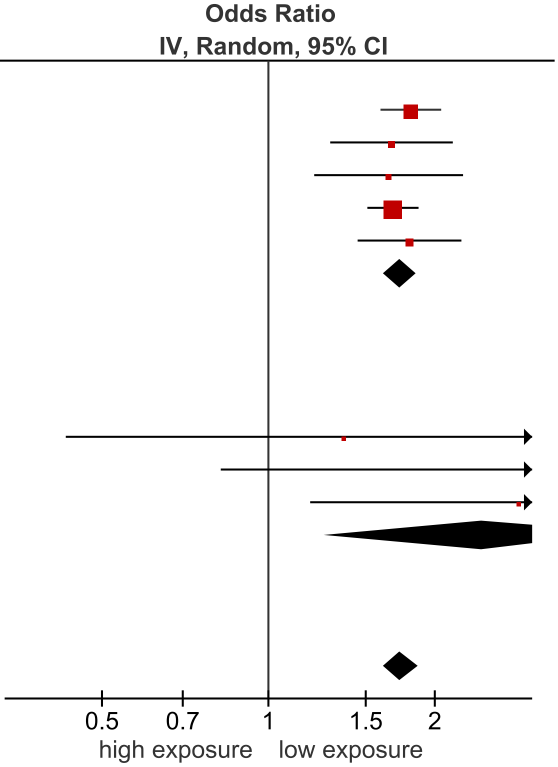

Study or Subgroup	log[Odds Ratio]	SE	Weight	Odds Ratio IV, Random, 95% CI
**1.1.1 Men**				
Bültmann	0.4121	0.1041	10.3%	1.51 [1.23, 1.85]
Magnusson Hanson	0.7372	0.1625	6.2%	2.09 [1.52, 2.87]
Oshio 2017 man	0.4824	0.0566	15.1%	1.62 [1.45, 1.81]
Stansfeld	0.2852	0.0956	11.1%	1.33 [1.10, 1.60]
**Subtotal (95% CI)**			**42.8%**	**1.57 [1.36, 1.80]**
Heterogeneity: Tau² = 0.01; Chi² = 6.56, df = 3 (P = 0.09); I² = 54%				
Test for overall effect: Z = 6.31 (P < 0.00001)				
**1.1.2 Women**				
Bültmann	0.3646	0.1706	5.8%	1.44 [1.03, 2.01]
Magnusson Hanson	0.5822	0.1402	7.5%	1.79 [1.36, 2.36]
Oshio 2017 vrouw	0.5365	0.1094	9.8%	1.71 [1.38, 2.12]
Stansfeld	0.2151	0.1199	9.0%	1.24 [0.98, 1.57]
**Subtotal (95% CI)**			**32.2%**	**1.53 [1.29, 1.82]**
Heterogeneity: Tau² = 0.01; Chi² = 5.48, df = 3 (P = 0.14); I² = 45%				
Test for overall effect: Z = 4.77 (P < 0.00001)				
**1.1.3 All**				
Andersen 2017	0.4762	0.1457	7.2%	1.61 [1.21, 2.14]
Devereux	0.4824	0.1282	8.3%	1.62 [1.26, 2.08]
Mino	0.2231	0.1319	8.1%	1.25 [0.97, 1.62]
Sundin	1.4656	0.3992	1.4%	4.33 [1.98, 9.47]
**Subtotal (95% CI)**			**25.0%**	**1.65 [1.24, 2.21]**
Heterogeneity: Tau² = 0.05; Chi² = 9.46, df = 3 (P = 0.02); I² = 68%				
Test for overall effect: Z = 3.40 (P = 0.0007)				
**Total (95% CI)**			**100.0%**	**1.56 [1.41, 1.72]**
Heterogeneity: Tau² = 0.01; Chi² = 21.60, df = 11 (P = 0.03); I² = 49%				
Test for overall effect: Z = 8.94 (P < 0.00001)				
Test for subgroup differences: Chi² = 0.20, df = 2 (P = 0.91), I² = 0%				

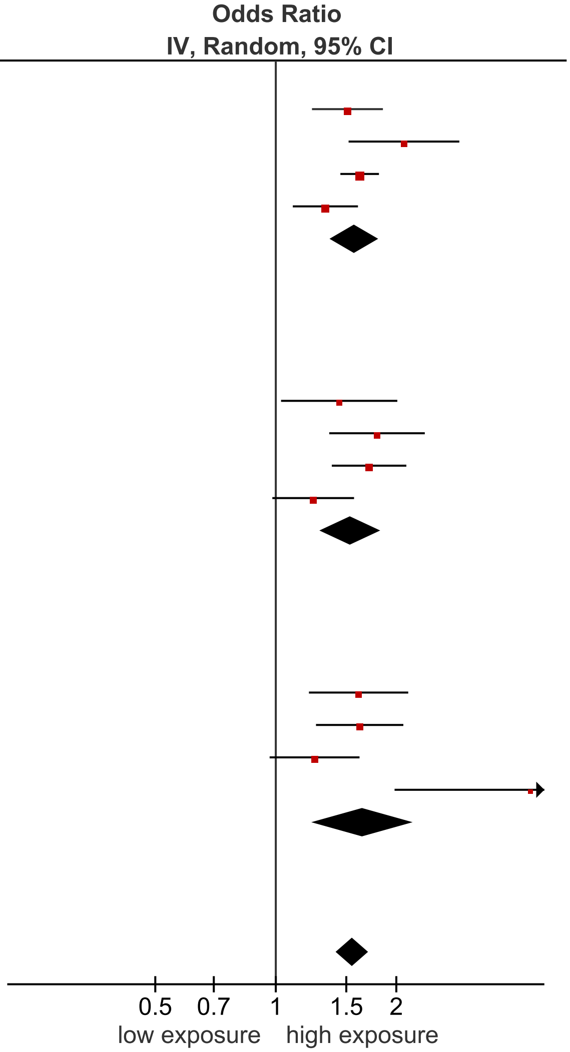

Appendix Search strategy Psychosocial risk factors and SRDs 12-08-2019
===
Medline
==

1. exp Occupations/ or Workload/ or exp Work/ or Workplace/ or exp Occupational Diseases/ or Rehabilitation, Vocational/ or Occupational Health/ or Sick Leave/ or Absenteeism/ or Retirement/ or workers' compensation/ or exp Employment/ or exp Occupational Exposure/ or Volunteers/
2. (worka* or worke* or workg* or worki* or workl* or workp* or work capacity or work disabilit* or work abilit* or at work or work exposure or work related or workers or job* or employee or staff or personnel or occupation or occupations or occupational or outdoor work* or day shift* or night shift* or shift work* or vocational rehabilitation or sick leave or absenteeism or sickness absen* or absente* or presente* or return to work or vocational reintegration or retirement or pension or employment or unemployed or unemployment or work status or industries or volunteer* or voluntary worker*).ab,kw,ti.
3. or/1-2 [working population]
4. risk/ or risk assessment/ or risk factors/ or cohort studies/ or Follow-Up Studies/
5. (risk* or Case control or cohort stud* or Cohort analy* or follow up stud* or observational stud* or Longitudinal).ab,kw,ti.
6. or/4-5 [aetiology]
7. Burnout, Professional/ or neurasthenia/ or adjustment disorders/
8. (burnout or burn out or neurasthenia or adjustment disorder? or emotional exhaustion or job stress or work stress or occupational stress or job strain or work strain).ab,kw,ti.
9. (occupational health/ or occupational disease/) and (psychiatric disorder? or psychiatric morbidity or psychosocial or psycho social or occupational stress or distress).ab,kw,ti.
10. or/7-9 [burnout]
11. (stress symptom* or psychologic* stress or psychologic* distress or psychosocial risk* or psycho social risk* or psychosocial factor* or psycho social factor* or psychiatric morbidit* or psychiatric disorder*).ab,kw,ti. [stress related symptoms and disorders]
12. 3 and 6 and 10
13. ((worka* or worke* or workg* or worki* or workl* or workp* or work capacity or work disabilit* or work abilit* or at work or work exposure or work related or workers or job* or employee or staff or personnel or occupation or occupations or occupational or outdoor work* or day shift* or night shift* or shift work* or vocational rehabilitation or sick leave or absenteeism or sickness absen* or absente* or presente* or return to work or vocational reintegration or retirement or pension or employment or unemployed or unemployment or work status or industries or volunteer* or voluntary worker*) adj1 (stress symptom* or psychologic* stress or psychologic* distress or psychosocial risk* or psycho social risk* or psychosocial factor* or psycho social factor* or psychiatric morbidit* or psychiatric disorder*)).ab,kw,ti.
14. 6 and 13
15. ((risk* or Case control or cohort stud* or Cohort analy* or follow up stud* or observational stud* or Longitudinal) adj1 (stress symptom* or psychologic* stress or psychologic* distress or psychosocial risk* or psycho social risk* or psychosocial factor* or psycho social factor* or psychiatric morbidit* or psychiatric disorder*)).ab,kw,ti.
16. 3 and 15
17. 12 or 14 or 16
18. limit 17 to (dutch or english or german)

==
PsycINFO
==

1. (occupational employment testing or occupational interests guidance or occupational vocational rehabilitation or working conditions industrial safety).cc.
2. exp occupations/ or career change/ or career development/ or exp "division of labor"/ or employment history/ or exp job characteristics/ or occupational adjustment/ or occupational aspirations/ or occupational attitudes/ or occupational choice/ or occupational exposure/ or occupational guidance/ or occupational interests/ or occupational mobility/ or occupational preference/ or occupational safety/ or occupational status/ or occupational success/ or exp occupational tenure/ or exp personnel/ or exp professional personnel/ or working women/ or "work (attitudes toward)"/ or Workplace Violence/ or Diversity in the Workplace/ or exp working conditions/ or exp vocational rehabilitation/ or Occupational Health/ or employee absenteeism/ or Retirement/ or exp employment status/ or reemployment/ or Volunteers/
3. (worka* or worke* or workg* or worki* or workl* or workp* or work capacity or work disabilit* or work abilit* or at work or work exposure or work related or workers or job* or employee or staff or personnel or occupation or occupations or occupational or outdoor work* or day shift* or night shift* or shift work* or vocational rehabilitation or sick leave or absenteeism or sickness absen* or absente* or presente* or return to work or vocational reintegration or retirement or pension or employment or unemployed or unemployment or work status or industries or volunteer* or voluntary worker*).ab,id,ti.
4. or/1-3 [working population]

For peer review only

5. Risk Assessment/ or At Risk Populations/ or Risk Factors/ or clinical trials/ or cohort analysis/ or followup studies/ or exp longitudinal studies/
6. (risk* or Case control or cohort stud* or Cohort analy* or follow up stud* or observational stud* or Longitudinal).ab,id,ti.
7. or/5-6 [aetiology]
8. occupational stress/ or work related illnesses/ or neurastenia/ or adjustment disorders/
9. (neurasthenia or burnout or burn out or emotional exhaustion or job stress or work stress or occupational stress or job strain or work strain or adjustment disorder?).ab,id,ti.
10. 8 or 9 [burnout]
11. (stress or distress).ab,id,ti.
12. 4 and 7 and 10
13. ((risk* or Case control or cohort stud* or Cohort analy* or follow up stud* or observational stud* or Longitudinal) adj3 (stress or distress)).ab,id,ti.
14. 4 and 13
15. 12 or 14
16. limit 15 to ("0100 journal" or "0110 peer-reviewed journal" or "0120 non-peer-reviewed journal" or "0130 peer-reviewed status unknown" or "0400 dissertation abstract")
17. limit 16 to (dutch or english or german)

==

Embase

==

1. exp *occupation/ or exp *occupational health/ or *work/ or *workplace/ or *"employment of women"/ or exp *income/ or *workman compensation/ or exp *employment/ or exp *"named groups by occupation"/
2. (worka* or worke* or workg* or worki* or workl* or workp* or work capacity or work disabilit* or work abilit* or at work or work exposure or work related or workers or job* or employee or staff or personnel or occupation or occupations or occupational or outdoor work* or day shift* or night shift* or shift work* or vocational rehabilitation or sick leave or absenteeism or sickness absen* or absente* or presente* or return to work or vocational reintegration or retirement or pension or employment or unemployed or unemployment or work status or industries or volunteer* or voluntary worker*).ab,kw,ti.
3. or/1-2 [working population]
4. *risk/ or *risk assessment/ or *risk factor/ or *cohort analysis/ or *Follow-Up/
5. (risk* or Case control or cohort stud* or Cohort analy* or follow up stud* or observational stud* or Longitudinal).ab,kw,ti.
6. or/4-5 [etiology]
7. *Burnout/ or *neurasthenia/ or *adjustment disorder/ or *emotional stress/ or *job stress/
8. (burnout or burn out or neurasthenia or adjustment disorder? or emotional exhaustion or job stress or work stress or occupational stress or job strain or work strain).ab,kw,ti.
9. (occupational health/ or occupational disease/) and (psychiatric disorder? or psychiatric morbidity or psychosocial or psycho social or occupational stress or distress).ab,kw,ti.
10. or/7-9 [burnout]
11. (stress symptom* or psychologic* stress or psychologic* distress or psychosocial risk* or psycho social risk* or psychosocial factor* or psycho social factor* or psychiatric morbidit* or psychiatric disorder*).ab,kw,ti. [stress related symptoms and disorders]
12. 3 and 6 and 10
13. ((worka* or worke* or workg* or worki* or workl* or workp* or work capacity or work disabilit* or work abilit* or at work or work exposure or work related or workers or job* or employee or staff or personnel or occupation or occupations or occupational or outdoor work* or day shift* or night shift* or shift work* or vocational rehabilitation or sick leave or absenteeism or sickness absen* or absente* or presente* or return to work or vocational reintegration or retirement or pension or employment or unemployed or unemployment or work status or industries or volunteer* or voluntary worker*) adj1 (stress symptom* or psychologic* stress or psychologic* distress or psychosocial risk* or psycho social risk* or psychosocial factor* or psycho social factor* or psychiatric morbidit* or psychiatric disorder*)).ab,kw,ti.
14. 6 and 11 and 13
15. ((risk* or Case control or cohort stud* or Cohort analy* or follow up stud* or observational stud* or Longitudinal) adj1 (stress symptom* or psychologic* stress or psychologic* distress or psychosocial risk* or psycho social risk* or psychosocial factor* or psycho social factor* or psychiatric morbidit* or psychiatric disorder*)).ab,kw,ti.
16. 3 and 15
17. 12 or 14 or 16
18. limit 17 to (dutch or english or german)

For peer review only - http://bmjopen.bmj.com/site/about/guidelines.xhtml

PRISMA 2009 Checklist

Section/topic	#	Checklist item	Reported on page #
**TITLE**			
Title	1	Identify the report as a systematic review, meta-analysis, or both.	1
**ABSTRACT**			
Structured summary	2	Provide a structured summary including, as applicable: background; objectives; data sources; study eligibility criteria, participants, and interventions; study appraisal and synthesis methods; results; limitations; conclusions and implications of key findings; systematic review registration number.	2
**INTRODUCTION**			
Rationale	3	Describe the rationale for the review in the context of what is already known.	5
Objectives	4	Provide an explicit statement of questions being addressed with reference to participants, interventions, comparisons, outcomes, and study design (PICOS).	5 and 8
**METHODS**			
Protocol and registration	5	Indicate if a review protocol exists, if and where it can be accessed (e.g., Web address), and, if available, provide registration information including registration number.	n/a
Eligibility criteria	6	Specify study characteristics (e.g., PICOS, length of follow-up) and report characteristics (e.g., years considered, language, publication status) used as criteria for eligibility, giving rationale.	6,7
Information sources	7	Describe all information sources (e.g., databases with dates of coverage, contact with study authors to identify additional studies) in the search and date last searched.	7,8
Search	8	Present full electronic search strategy for at least one database, including any limits used, such that it could be repeated.	Appendix 1
Study selection	9	State the process for selecting studies (i.e., screening, eligibility, included in systematic review, and, if applicable, included in the meta-analysis).	7,8,9,10
Data collection process	10	Describe method of data extraction from reports (e.g., piloted forms, independently, in duplicate) and any processes for obtaining and confirming data from investigators.	8
Data items	11	List and define all variables for which data were sought (e.g., PICOS, funding sources) and any assumptions and simplifications made.	8,9
Risk of bias in individual studies	12	Describe methods used for assessing risk of bias of individual studies (including specification of whether this was done at the study or outcome level), and how this information is to be used in any data synthesis.	8,9
Summary measures	13	State the principal summary measures (e.g., risk ratio, difference in means).	7
Synthesis of results	14	Describe the methods of handling data and combining results of studies, if done, including measures of consistency (e.g., I^2) for each meta-analysis.	9

For peer review only - http://bmjopen.bmj.com/site/about/guidelines.xhtml

PRISMA 2009 Checklist

Section/topic	#	Checklist item	Reported on page #
Risk of bias across studies	15	Specify any assessment of risk of bias that may affect the cumulative evidence (e.g., publication bias, selective reporting within studies).	9,10, table 3
Additional analyses	16	Describe methods of additional analyses (e.g., sensitivity or subgroup analyses, meta-regression), if done, indicating which were pre-specified.	n/a
**RESULTS**			
Study selection	17	Give numbers of studies screened, assessed for eligibility, and included in the review, with reasons for exclusions at each stage, ideally with a flow diagram.	11<br>Figure 1
Study characteristics	18	For each study, present characteristics for which data were extracted (e.g., study size, PICOS, follow-up period) and provide the citations.	Table 1
Risk of bias within studies	19	Present data on risk of bias of each study and, if available, any outcome level assessment (see item 12).	Table 2
Results of individual studies	20	For all outcomes considered (benefits or harms), present, for each study: (a) simple summary data for each intervention group (b) effect estimates and confidence intervals, ideally with a forest plot.	Table 1
Synthesis of results	21	Present results of each meta-analysis done, including confidence intervals and measures of consistency.	18-20<br>Figure 2.1-2.3
Risk of bias across studies	22	Present results of any assessment of risk of bias across studies (see Item 15).	Table 3
Additional analysis	23	Give results of additional analyses, if done (e.g., sensitivity or subgroup analyses, meta-regression [see Item 16]).	n.a.
**DISCUSSION**			
Summary of evidence	24	Summarize the main findings including the strength of evidence for each main outcome; consider their relevance to key groups (e.g., healthcare providers, users, and policy makers).	21,22
Limitations	25	Discuss limitations at study and outcome level (e.g., risk of bias), and at review-level (e.g., incomplete retrieval of identified research, reporting bias).	22,23
Conclusions	26	Provide a general interpretation of the results in the context of other evidence, and implications for future research.	24,25
**FUNDING**			
Funding	27	Describe sources of funding for the systematic review and other support (e.g., supply of data); role of funders for the systematic review.	25

From: Moher D, Liberati A, Tetzlaff J, Altman DG, The PRISMA Group (2009). Preferred Reporting Items for Systematic Reviews and Meta-Analyses: The PRISMA Statement. PLoS Med 6(7): e1000097. doi:10.1371/journal.pmed1000097

For more information, visit: www.prisma-statement.org.

For peer review only - http://bmjopen.bmj.com/site/about/guidelines.xhtml
